# Causality between gut microbiota, immune cells, and breast cancer: Mendelian randomization analysis

**DOI:** 10.1097/MD.0000000000040815

**Published:** 2024-12-06

**Authors:** Rui Lv, Danyan Wang, Tengyue Wang, Rongqun Li, Aiwen Zhuang

**Affiliations:** a School of Basic Medical Sciences, Zhejiang Chinese Medical University, Hangzhou, China; b Zhuji Second People’s Hospital, Zhuji, China; c Institute of TCM Literature and Information, Zhejiang Academy of Traditional Chinese Medicine, Hangzhou, China.

**Keywords:** breast cancer, causality, gut microbiota, immune cells, Mendelian randomization

## Abstract

The association between gut microbiota (GM) and breast cancer (BC) has been studied. Nevertheless, the causal relationship between them and the potential mediating factors have not been clearly defined. Therefore, in this study, Mendelian randomization analysis (MR) was employed to explore the causal relationship between 473 GM and BC, as well as the mediating effect of potential immune cells. In this investigation, we availed ourselves of the publicly accessible summary statistics from the genome-wide association study to undertake two-sample and reverse Mendelian randomization analyses on GM and BC, with the intention of clarifying the causal association between GM and BC. Subsequently, through the application of the two-step Mendelian randomization analysis, it was revealed that the relationship between GM and BC was mediated by immune cells. The stability of the research outcomes was verified via sensitivity analysis. Mendelian randomization analysis elucidated the protective impacts of 8 genera on BC (such as Phylum Actinobacteriota, Species *Bacteroides* A *plebeius* A, Species *Bifidobacterium adolescentis*, Species CAG-841 sp002479075, Family Fibrobacteraceae, Order Fibrobacterales, Class Fibrobacteria, and Species Phascolarctobacterium sp003150755). Additionally, there are 23 immune cell traits related to BC. Our research findings showed that the species *Megamonas funiformis* was associated with an increased risk of BC, and 11.20% of this effect was mediated by CD38 on IgD+ CD24‐. Likewise, HLA DR on CD33br HLA DR+ CD14‐ mediated the causal relationship between Species *Prevotellamassilia* and BC, having a mediating ratio of 7.89%. This study clarifies a potential causal relationship between GM, immune cells, and BC and provides genetic evidence for this causal connection. It offers research directions for the subsequent prevention and treatment of BC through the interaction between GM and immune cells, and provides a reference for future mechanistic and clinical studies in this field.

## 1. Introduction

Breast cancer is the most prevalent malignant tumor afflicting women globally. Women are severely impacted by it, and the incidence rate ascends with age: over 80% of breast cancer (BC) cases are identified in women above 50 years old.^[1]^ Clinically, specific subtypes of BC are defined based on their histopathological manifestations and the expression of hormone receptors and growth factors (such as the estrogen receptor, the progesterone receptor, and the human epidermal growth factor receptor 2^[2,3]^). Although there are distinct genetic risk factors for BC (such as BRCA1/2 mutations) and environmental risk factors (such as a sedentary lifestyle, obesity, alcohol intake, and hormone replacement therapy), yet the majority of sporadic cases arise in women of average risk. This suggests that there might be other unidentified risk factors.^[4]^ According to the retrospective trend analysis of the World Health Organization, BC constitutes 23% of all cancers and is the second major factor contributing to cancer deaths, having a mortality rate of 14%. Hence, in an effort to develop novel, safe and efficacious BC treatment approaches to lower the mortality rate, it is essential to acquire a more profound comprehension of the intricate causes of BC. In recent years, the gut microbiota (GM) has been considered as the second genome of human beings, garnering significant attention from researchers and exerting a crucial role in the domain of human health.^[5,6]^ Epidemiological studies have demonstrated that the GM gives rise to 16% to 18% or even a greater proportion of malignant tumors globally.^[7]^ The freshly emerging evidence is increasingly emphasizing the connection between the composition of the GM and the development as well as the aggressiveness of cancer.^[8]^ Such relationships like the role of *Helicobacter pylori* in gastric cancer and that of Fusobacterium in colorectal cancer have been reported.^[9,10]^ However, the understanding of the association between GM and BC is rather limited. The link between GM and BC initially stemmed from epidemiological studies, which initiated inquiries into the influence of antibiotic use on BC.^[11]^ By reducing estrogen metabolism via the GM, it is possible to lower circulating estrogen and thereby decrease the risk of developing estrogen receptor-positive BC.^[12,13]^ However, epidemiological studies might encounter certain limitations, for instance, measurement errors, uncontrolled confounding factors, and reverse causality. Eventually, their outcomes might be influenced by diverse biases. Hence, a design is requisite to further evade or diminish some biases in order to affirm the causal relationship between the GM and BC. Furthermore, the potential pathways associated with the GM and BC have not been explored. Previous studies have furnished evidence suggesting that immune cells possess causal ties with both the GM and BC.^[14,15]^ Hence, immune cells may act as the mediator between the GM and BC. Studies have demonstrated that the immune system surveils newly transformed cells and plays a crucial role in cancer prevention and tumor immune editing.^[16,17]^ Nevertheless, GM can facilitate the development of malignant tumors by stimulating unregulated inflammatory immune responses. Given the function of GM imbalance in chronic inflammation, inflammation-mediated carcinogenesis processes, and immune escape, it is not astonishing that specific GM are correlated with the development of specific cancers.^[18,19]^

Mendelian randomization (MR) constitutes a potential causal inference modality, employing genetic variations as instrumental variables to acquire the effect of exposure factors on the outcome from observational data.^[20]^ This approach utilizes genotypes that determine intermediate phenotypes like exposure characteristics to forge associations with disease outcomes.^[21,22]^ MR is capable of diminishing the influence of non-measurement errors or confounding factors in accordance with Mendel laws of inheritance, and concurrently evade reverse causality.^[20]^ The objective of this study is to investigate the causal effect of the GM on breast cancer and whether it can be mediated by immune cells. Initially, we gathered single nucleotide polymorphism (SNP) data as the instrumental variables (IVs) of exposure. Subsequently, a comprehensive two-sample MR analysis was conducted to assess the causal effect of the GM and immune cell characteristics on breast cancer. Eventually, we explored the influence of the GM on immune cell characteristics and computed the proportion of the impact of the GM on breast cancer via immune cell characteristics to evaluate whether the GM can impact the progression of breast cancer by regulating the immune system.

## 2. Methods

### 2.1. Study design

**Figure 1.**
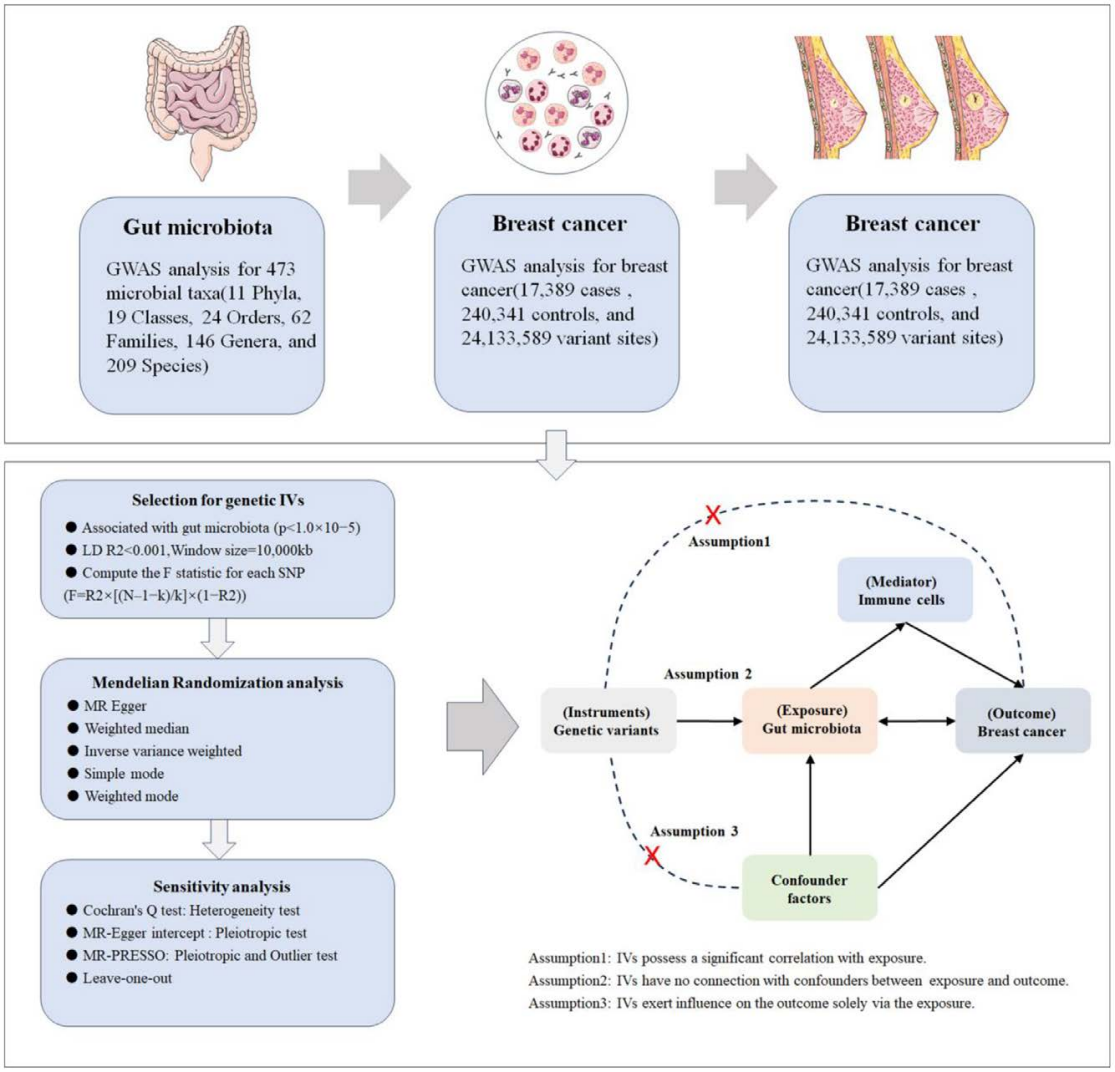
Study design and workflow of MR analysis. AC = absolute cell number, LD = linkage disequilibrium, MFI = median fluorescence intensity, MP = morphological parameter, MR = Mendelian randomization, MR-PRESSO = MR pleiotropy residual sum and outlier, RC = relative cell number.

In this study, we employed two-sample Mendelian randomization to delve into the causal relationship between the GM and breast cancer. We conducted a two-step Mendelian randomization to ascertain the association between the GM and the risk of breast cancer, and whether immune cell traits could act as mediators for this association. The research design comprises 3 parts: (1) to assess the causal relationship between the GM and breast cancer and screen out the GM highly correlated with the risk of breast cancer. (2) To evaluate the causal effect of immune cell characteristics on breast cancer and screen out the immune cell characteristics highly related to the risk of breast cancer. (3) To combine the causal effect of the screened GM on the screened immune cell characteristics and calculate the mediation proportion of the mediator for the effect of the GM on breast cancer. The study design is illustrated in Figure 1.

### 2.2. Source and object of study

For the data of microbial taxa, a genome-wide association test was conducted, which included 2801 microbial taxa from 5959 individuals in the FINRISK 2002 cohort and 7,967,866 human genetic variants. The GM was determined by matching the human genome and performing shotgun metagenomic sequencing on fecal samples (N = 5959; FINRISK 2002). Using a genome-wide significance threshold (*P* < 5.0 × 10^−8^), 471 different Genome Taxonomy Database taxa were included (covering 11 phyla, 19 classes, 24 orders, 62 families, 146 genera, and 209 species). In this study, 473 genera were included (from GCST90032172 to GCST90032644).^[23]^

For the data of mediator immune cell traits relied on the summary statistics of the extensive genome-wide association study (GWAS) that was recently carried out by the Blood Cell Consortium on blood cell traits. This GWAS encompassed a vast cohort composed of 563,085 individuals of European descent.^[24]^ The 731 immunophenotypes comprised median fluorescence intensity (n = 389), absolute cell number (AC) (n = 118), relative cell number (n = 192), and morphological parameter (n = 32). The first 3 types encompassed myeloid cells, B cells, T cell maturation stage, monocytes, T cells, B cells, natural killer cells, CDCs, and Treg panels, while the latter incorporated CDCs and T cells, B cells, natural killer cells panels.^[25]^

For the data of breast cancer-related genetic variation stemmed from the MRC – IEU Alliance (IEU OpenGWAS) database. Sakaue S et al conducted 220 deep phenotypic genome-wide association studies (diseases, biomarkers, and drug use) in BioBank Japan (n = 179,000), providing genetic association data for 257,730 Europeans. This included 17,389 patients and 240,341 controls, with 24,133,589 variant sites.^[26]^ To ensure the consistency of the data, all the participants hail from Europe. The exhaustive information is available in Table S1, Supplemental Digital Content, http://links.lww.com/MD/O102.

### 2.3. Selection for genetic variation

To acquire reliable outcomes, two-sample MR is required to fulfill 3 fundamental assumptions (Fig. 1): (1) IVs must possess a significant correlation with the GM. (2) IVs have no connection with other factors that might confound the relationship between exposure variables and outcome variables. (3) IVs should exert influence on the outcome solely via the GM. Initially, we employed R software to extract sSNPs from the GWAS summary data associated with exposures. Only those exposures that demonstrated a genome-wide significant association (*P* < 5 × 10^−8^) with the traits were determined as IVs. In the case where no genome-wide significant SNPs were available as IVs, we adjusted the genome-wide significance level to 5 × 10^−5^.^[27]^ After that, we grouped all these genetic variants into a linkage disequilibrium threshold of *r*^2^ < 0.001 and a genetic distance of 10,000 kb.^[28]^ Eventually, we computed the *F* statistic for each SNP to detect any weak IV deviation in our analysis.^[29]^ We computed the *F* statistic in accordance with the given formula: *F* = *R*^2^×[(*N*–1 − *k*)/*k*] × (1 − *R*^2^), where *K* represents the number of genetic variations, N indicates the size of the sample, and *R*^2^ stands for the total variance accounted for by the selected SNPs. SNPs having an *F* statistic lower than 10 suggest that there might exist a weak IV bias. To guarantee the accuracy of the results, these were excluded from the study.^[30]^ The chosen SNPs are elaborated in Table S2, Supplemental Digital Content, http://links.lww.com/MD/O102.

### 2.4. Statistical analysis

#### 2.4.1. Primary analysis

We carried out a two-sample MR analysis to assess the relationship between GM and breast cancer, and designated it as the total effect. Inverse variance weighting (IVW) was considered as the primary approach for causal estimation, which is a dependable method in MR analysis.^[31]^ The Wald ratio of individual SNPs was computed via the formula (β_IV_ = β_ZY_/β_ZX_) to estimate the risk of exposure to the outcome. Additionally, MR-Egger, weighted median, weighted mode, and simple mode were utilized as complements to IVW.^[21,32,33]^ To acquire MR estimates, diverse methods were utilized in accordance with different validity assumptions. The application of IVW rests on the presumption that all genetic variant SNPs are valid instrumental variables. When horizontal pleiotropy is present, even if 50% of the genetic variants are invalid IVs, the weighted median is capable of offering a consistent estimate.^[34]^ Hence, this method can generate precise estimation results.

#### 2.4.2. Sensitivity analysis

To assess the robustness of the MR results, ascertain the reliability of the conclusions, and concurrently detect the potential bias and the impact of the instrumental variables on the outcome variables, a sensitivity analysis was conducted. We employed the *Q* test (heterogeneity was determined when *P* < .05 in accordance with IVW or MR-egger) to measure heterogeneity. When confronted with heterogeneity, we chose the random effect IVW in the main analysis. We assessed the potential impact of directional pleiotropy by inspecting the intercept value in the MR-Egger regression.^[35]^ The intercept is capable of testing gene pleiotropy. When the intercept is closer to 0, it is regarded that the influence of gene pleiotropy is minor (when *P* > .05, it is considered that the possibility of gene pleiotropy in causal analysis is weak and its influence can be disregarded). We further used the MR-PRESSO method to detect possible outliers and calculate causal estimates after removing the identified ones.^[35]^ To clarify if the causal relationship is driven by a single SNP, we adopted the leave-one-out sensitivity analysis. Additionally, using forest plots, scatter plots, and funnel plots showed that the data were stable and there was no heterogeneity. All analyses were conducted in R 4.3.1 software, employing the “Two Sample MR,” “gwasglue,” and “VariantAnnotation” packages for data processing and result visualization (https://www.r-project.org).

#### 2.4.3. Mediation analysis

**Figure 2.**
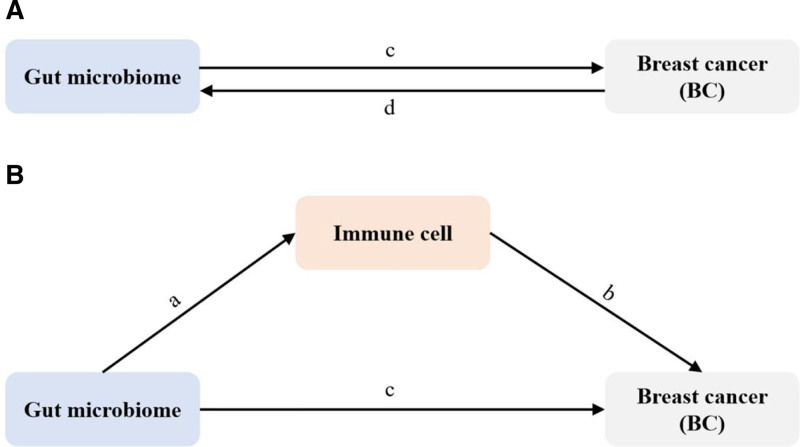
The diagram illustrates the associations examined in this study. *Notes*: (A) this is the total effect between gut microbiota (GM) and breast cancer (BC). *c* is the total effect with GM as exposure and BC as outcome, and *d* is the total effect with BC as exposure and GM as outcome; (B) the total effect is decomposed into: (i) the indirect effect using the two-step method (where a is the total effect of GM on immune cell, *b* is the total effect of immune on BC and the indirect effect using the product method (*a* × *b*)); (ii) direct effect (*c*′ = *c* ‐ *a* × *b*). The intermediary ratio is the indirect effect divided by the total effect.

Mediation analysis is a methodology that breaks down the direct impact of exposure (GM) on the outcome (breast cancer) and the influence generated via mediating variables. We carried out a mediation analysis by employing a two-step MR design to investigate whether immune cells mediate the causal pathway from GM to breast cancer outcome. The overall effect can be disintegrated into an indirect effect (via mediators) and a direct effect (without mediators).^[36]^ The total effect of GM on breast cancer can be disaggregated into (1) the direct effect of GM on breast cancer (c′) and (2) the indirect effect produced by GM via the mediating variable (a × b). We divide the indirect effect by the total effect to compute the percentage mediated by the mediating effect. The illustration of the mediation analysis is presented as follows. As shown in Figure 2.

## 3. Results

### 3.1. Association of gut microbiota and breast cancer

**Figure 3.**
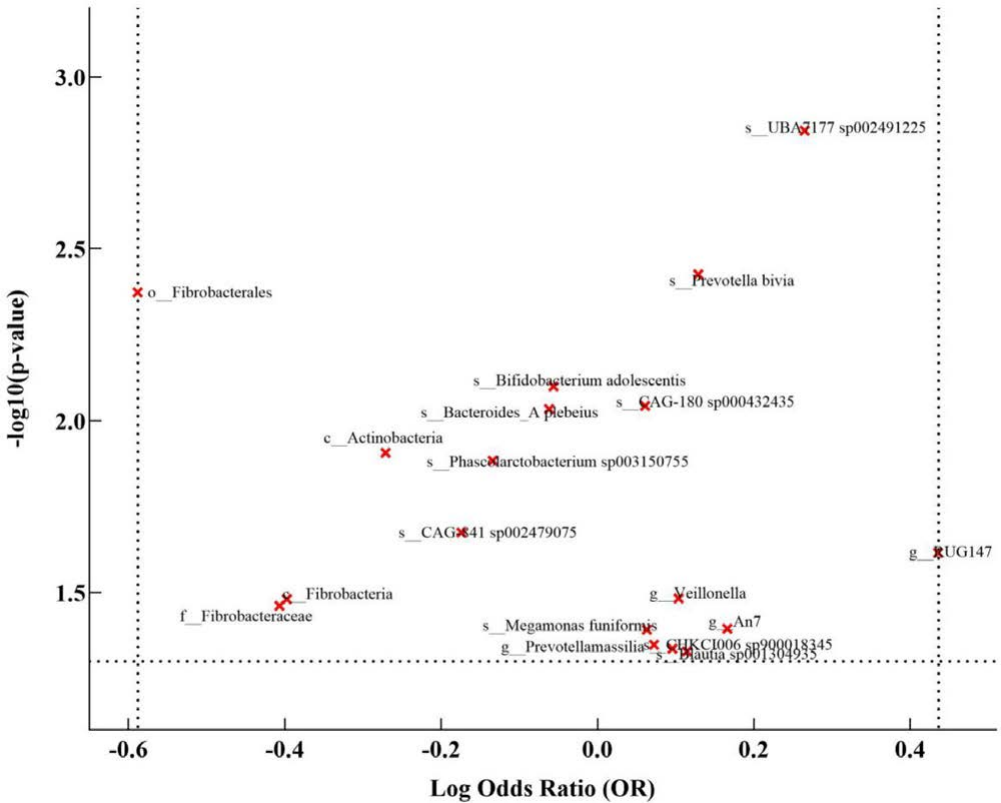
Mendelian randomization analysis between GM and BC. *Notes*: The volcano plot visually illustrates the associations between GM exposures and BC. The *x*-axis represents the adjusted Log OR, indicating the direction and strength of the association, while they-axis showcases the ‐Log10(*P*-value) for significance levels. Exposures are color-coded, with red dots signifying significant associations (*P*-value < .05) and gray dots denoting nonsignificant relationships. BC = breast cancer, GM = gut microbiota.

Through the use of Mendelian randomization, we carried out an in-depth investigation into the association between GM and breast cancer. Relying on the IVW method, we determined 18 GM that could potentially be related to breast cancer. The result analysis is presented in Figure 3. Among these, Genus An7 (OR = 1.1803, 95% CI: 1.0074–1.3830, *P* = .0403), Species Blautia sp001304935 (OR = 1.1210, 95% CI: 1.0016–1.2547, *P* = .0469), Species CAG-180 sp000432435 (OR = 1.0627, 95% CI: 1.0153–1.1124, *P* = .0091), Species CHKCI006 sp900018345 (OR = 1.1003, 95% CI: 1.0017–1.2086, *P* = .0460), Species *Megamonas funiformis* (OR = 1.0649, 95% CI: 1.0027–1.1310, *P* = .0405), Species *Prevotella bivia* (OR = 1.1374, 95% CI: 1.0426–1.2409, *P* = .0038), Species *Prevotellamassilia* (OR = 1.0745, 95% CI: 1.0016–1.1527, *P* = .0449), Genus RUG147 (OR = 1.5459, 95% CI: 1.0583–2.2580, *P* = .0242), Species UBA7177 sp002491225 (OR = 1.3033, 95% CI: 1.1074–1.5338, *P* = .0014), and Genus Veillonella (OR = 1.1091, 95% CI: 1.0085–1.2197, *P* = .0329) all appear to be associated with an elevated risk of breast cancer. Phylum Actinobacteriota (OR = 0.7624, 95% CI: 0.6163–0.9430, *P* = .0124), Species *Bacteroides* A *plebeius* A (OR = 0.9398, 95% CI: 0.8968–0.9848, *P* = .0093), Species *Bifidobacterium adolescentis* (OR = 0.9452, 95% CI: 0.9067–0.9854, *P* = .0080), Species CAG-841 sp002479075 (OR = 0.8401, 95% CI: 0.7244–0.9743, *P* = .0212), Family Fibrobacteraceae (OR = 0.6657, 95%CI: 0.4565–0.9708, *P* = .0345), Order Fibrobacterales (OR = 0.5553, 95% CI: 0.3710–0.8310, *P* = .0042), Class Fibrobacteria (OR = 0.6720, 95% CI: 0.4663–0.9685, *P* = .0330), and Species Phascolarctobacterium sp003150755 (OR = 0.8743, 95% CI = 0.7864–0.9722, *P* = .0131) demonstrated a protective effect and might potentially reduce the risk of breast cancer. Based on Cochran *Q* (*P*-value < .05), there was evidence of heterogeneity in breast cancer analysis. So, random effects MR estimates were used for these models. The MR-Egger regression intercept terms were about 0 (*P* > .05), suggesting no significant horizontal pleiotropic effects (Table S4, Supplemental Digital Content, http://links.lww.com/MD/O102). In the leave-one-out sensitivity analysis (Table S9, Supplemental Digital Content, http://links.lww.com/MD/O102), no single SNP violated the impact of GM on breast cancer. Reverse MR analysis was conducted to verify whether the observed GM was affected by breast cancer. In this analysis, BC was considered as the exposure variable and GM as the outcome variable. The results indicated that there was no evidence suggesting that breast cancer affects the GM in IVW (Table S3, Supplemental Digital Content, http://links.lww.com/MD/O102).

### 3.2. Association of immune cell traits and BC

**Figure 4.**
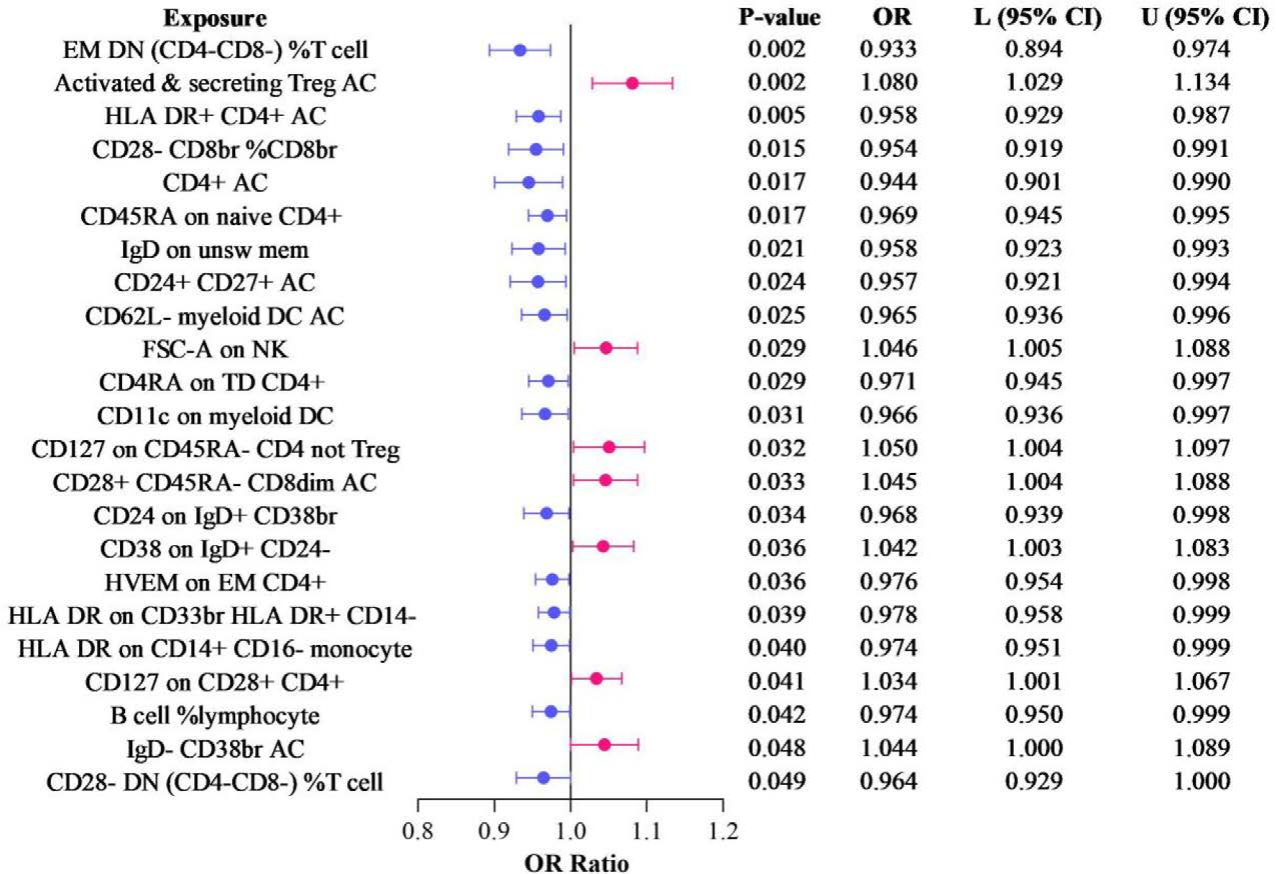
Forest plot of MR results with inverse-variance weighted approach. Mendelian randomization. MR = Mendelian randomization.

In our study for identifying potential mediators, 731 immune cell traits were initially picked to study their effect on breast cancer. Using the IVW method, we found 16 immune cell traits had a protective effect (Table S5, Supplemental Digital Content, http://links.lww.com/MD/O102). Also, 7 immune cell traits would increase the risk (Fig. 4). The percentage of CD24+ CD27+ AC is associated with a decreased risk (OR = 0.9569, 95% CI: 0.9210–0.9942, *P* = .0241). Similarly, the absolute counts of CD62L‐ myeloid DC AC, EM DN (CD4‐ CD8‐)%T cells, and CD4+ AC are respectively correlated with odds ratios of 0.9654 (*P* = .0247), 0.9334 (*P* = .0016), and 0.9444 (*P* = .0171). The presence of FSC-A on NK indicates an increased risk (OR = 1.0455, 95% CI: 1.0046–1.0881, *P* = .0289). Other significant associations encompass exposure factors such as CD28+ CD45RA‐ CD8dim AC (OR = 1.0449, 95% CI: 1.0037–1.0878, *P* = .0326) and CD11c on myeloid DC (OR = 0.9662, 95% CI: 0.9364–0.9969, *P* = .0312). These findings spotlight the intricate relationship between specific cellular markers and breast cancer, laying the foundation for subsequent mediation analyses. The *P* values derived from Cochran Q statistic obtained by the IVW and MR Egger methods are all >0.05, indicating that no obvious heterogeneity is detected. The MR-Egger intercept test is not statistically significant, suggesting that there is no horizontal pleiotropy (Table S6, Supplemental Digital Content, http://links.lww.com/MD/O102). The results of the leave-one-out analysis reveal that removing a specific SNP will not alter the causal estimates (Table S10, Supplemental Digital Content, http://links.lww.com/MD/O102).

### 3.3. Association of GM and immune cell traits

Subsequently, we conducted MR analysis on 18 selected GM and 23 immune cells to further explore the potential mediating role of GM exposure in these important mediators. Our analysis generated several significant findings. Six GMs were highly correlated with 7 immune cell traits (Table 1). Specifically, Genus An7 showed a positive mediating effect through CD11c on myeloid DC, with an effect size of 0.470 (*P* = .0116). Species *M funiformis* exhibited a positive mediating effect through CD38 on IgD+ CD24‐ and HVEM on EM CD4+, with effect sizes of 0.171 and 0.243, respectively (*P* = .0221 and *P* = .0269).Several other GM, such as Species *Bacteroides* A *plebeius* A (*P* = .0019), Family Fibrobacteraceae (*P* = .0311), and Species *Prevotella bivia* (*P* = .0296), also exhibited positive mediating effects through a range of immune cell signatures, with effect sizes of 0.226, 1.220, and 0.241, respectively. Species *Prevotellamassilia* exhibits a negative mediating effect by HLA DR on CD33br HLA DR+ CD14‐ with an effect size of ‐0.260 (*P* = .0199). No signs of heterogeneity and pleiotropy were detected in the causality of these SNPs according to the Q-statistic test, MR-Egger intercept test, and MR-PRESSO (Table S7, Supplemental Digital Content, http://links.lww.com/MD/O102 and Table S8, Supplemental Digital Content, http://links.lww.com/MD/O102).

**Table 1:** Mediation Mendelian randomization analyses of the causal effects among gut microbiota, immune cells, and breast cancer.

Exposure	Mediator	Outcome	Total effect (β)	A (β)	B (β)	Indirect effect (β)
Genus An7	CD11c on myeloid DC	Breast cancer	0.166	0.470	‐0.034	‐0.016
Species *Bacteroides* A *plebeius* A	CD127 on CD45RA‐ CD4 not Treg	Breast cancer	‐0.062	0.226	0.049	0.011
Family Fibrobacteraceae	CD127 on CD28+ CD4+	Breast cancer	‐0.407	1.220	0.033	0.041
Species *Megamonas funiformis*	CD38 on IgD+ CD24‐	Breast cancer	0.063	0.171	0.041	0.007
Species *Megamonas funiformis*	HVEM on EM CD4+	Breast cancer	0.166	0.243	‐0.024	‐0.016
Species *Prevotella bivia*	HLA DR+ CD4+ AC	Breast cancer	0.113	0.241	‐0.043	‐0.010
Species *Prevotellamassilia*	HLA DR on CD33br HLA DR+ CD14‐	Breast cancer	0.072	‐0.260	‐0.022	0.006

Total effect: the causal role of GM on BC; A: the causal role of GM on immune cell traits; B: the causal role of immune cell traits on BC is independent of the effect of the GM; β (indirect effect) = β (A) * β (B).

### 3.4. Proportion of the association between GM and BC mediated by immune cell traits

After having clarified the significant mediators that exert an influence on breast cancer and the subsequent consequences of exposure on mediation, we quantified the proportion of the mediation effect (Table 2). We identified CD38 on IgD+ CD24‐ as a mediator in the causal relationship between Species *M funiformis* and breast cancer. Additionally, HLA DR on CD33br HLA DR+ CD14‐ was identified as a mediator in the causal relationship between Species *Prevotellamassilia* and breast cancer. Finally, we discovered that “CD38 on IgD+ CD24‐” served as a mediator in the causal relationship between Species *M funiformis* and breast cancer, with a mediating ratio of 11.20%. Likewise, “HLA DR on CD33br HLA DR+ CD14‐” mediated the causal relationship between Species *Prevotellamassilia* and breast cancer, having a mediating ratio of 7.89%.

**Table 2:** Mediation effect between gut microbiota and breast cancer.

Exposure	Mediator	Total effect (β)	A (β)	B (β)	Indirect effect (β)	Mediation effect/total effect
*Breast cancer* (outcome)						
Species *Megamonas funiformis*	CD38 on IgD+ CD24-	0.0629	0.1705	0.0412	0.00702	11.20%
Species *Prevotellamassilia*	HLA DR on CD33br HLA DR+ CD14‐	0.072	‐0.260	‐0.022	0.006	7.89%

Total effect: the causal role of GM on BC; A: the causal role of GM on immune cell traits; B: the causal role of immune cell traits on BC is independent of the effect of the GM; β (indirect effect) = β (A) * β (B); the mediated proportion = β (indirect effect)/β (total effect).

## 4. Discussion

There is an association between the GM and numerous cancers. By employing two-sample MR, we found that 18 species of GM had extremely significant causal effects on breast cancer. Among them, Genus An7, Species Blautia sp001304935, CAG-180 sp000432435, Species CHKCI006 sp900018345, Species *M funiformis*, Species *P bivia*, Species *Prevotellamassilia*, Genus RUG147, Species UBA7177 sp002491225, and Genus Veillonella were positively correlated with breast cancer, signifying that an increase in the abundance of these GM might heighten the risk of breast cancer. In addition, we have found that some GM have a protective effect against breast cancer, including: Phylum Actinobacteriota, Species *Bacteroides* A *plebeius* A, Species *B adolescentis*, Species CAG-841 sp002479075, Family Fibrobacteraceae, Order Fibrobacterales, Class Fibrobacteria, and Species Phascolarctobacterium sp003150755. Studies have shown that Firmicutes and Bacteroidetes are the most abundant bacteria in the feces of women with early-stage breast cancer.^[37]^ Moreover, part of the GM can be used as biomarkers for the diagnosis and staging of breast cancer.^[38]^ The total abundance of Bacteroides, Clostridium clusters, *Clostridium scindens* cluster, *Clostridium prausnitzii* cluster, *C prausnitzii*, and Ruminococcus in the phase II/III clinical group was significantly higher than that in the clinical phase 0/I group.^[39]^ Monomonas belongs to the genus Megamonas, a gram-negative bacterium belonging to the phylum Firmicutes. In the human gastrointestinal tract, Firmicutes contains an important gene encoding β-glucuronidase that is the GUS gene. β-Glucuronidase can accelerate the early dissociation of estrogen in the intestine and increase the level of free estrogen, thus inducing the occurrence of breast cancer. In the GM, bacteria such as those of the Clostridaceae and Ruminococcaceae are also capable of producing β-glucuronidase.^[40,41]^ Other cancers are also closely related to the GM. In colon cancer, tumor invasion and metastatic spread may occur as a result of the aggregation of certain microbiota and excessive proliferation of *Fusobacterium nucleatum*.^[42]^ In addition, *Bacteroides fragilis* is capable of playing a protective role in colitis by regulating the inflammatory immune response within the intestinal tract.^[43]^ *H pylori* infection is an important factor in the development of gastric cancer. It can be seen that the GM is one of the key factors in the occurrence and progression of breast cancer.

Similar to the GM, immune cells play an indelible role in the occurrence and development of cancer. We found that *M funiformis* had an effect on breast cancer through CD38 on IgD+ CD24‐. CD38 can interact with M1 macrophages, neutrophils, and T cells to elicit a range of immune responses. Among them, T cells are key mediators of tumor destruction and possess the ability to promote the restoration of intestinal epithelial homeostasis.^[44,45]^ Moreover, their activities on the mucosal surface exert a complex balancing influence during acute inflammatory responses, enabling the host to generate protective immune responses and subsequently restore inhibitory effects. CD38 on IgD+ CD24‐ can also be expressed in a multitude of tumors and assumes a tumor-promoting role, such as cervical cancer within solid tumors, glioma, esophageal cancer, liver cancer, melanoma and lung cancer, as well as in hematological malignancies, for example, multiple myeloma and chronic lymphocytic leukemia.^[46–55]^ HVEM is an immunomodulatory molecule that is expressed on the surface of HVEM on EM CD4+. HVEM on EM CD4+ represents a subset of T cells that differentiates from naïve CD4+ T cells during the immune response. It is capable of rapidly activating, proliferating, and secreting cytokines to initiate the immune response.^[56]^ Ruffell et al discovered that breast cancer tissue contains infiltrations dominated by CD4 and CD8 T lymphocytes. HVEM on EM CD4+ can influence BC by modulating the immune response through CD4+ T cell differentiation. CD11c, a member of the integrin β2 adhesion molecular family, is expressed on the surface of myeloid dendritic cells and other immune cells.^[57]^ It can trigger the activation and differentiation of naïve T cells into effector cells through antigen presentation, which can have an impact on the occurrence and development of BC.^[58]^ CD127 on CD45RA‐ CD4 not Treg cells, the surface receptor CD127, is the Interleukin-7 (IL-7) receptor α chain. IL-7, a pleiotropic cytokine, primarily fulfills its role in the immune system by promoting lymphocyte development and ensuring homeostasis.^[59]^ IL-7 is thought to play a role in breast cancer pathogenesis, promoting the growth and survival of tumor cells in culture.^[60]^ In tumor immunity, HLA-DR+ CD4+ can activate other immune cells, attack tumor cells, and promote the apoptosis of tumor cells, thereby inhibiting tumor growth.^[61]^ HLA DR on CD33br HLA DR+ CD14‐ integrates the associated characteristics of CD33, HLA DR, and CD14 and may serve as a specialized antigen-presenting cell. The HLA-DR molecule binds antigenic peptides and presents these to T cells, thus initiating adaptive immune responses. It plays a complex role in tumor immunity. On the one hand, they may be involved in antitumor immune responses by presenting tumor antigens that activate T cells; On the other hand, it may also be induced by tumor cells to produce immunosuppressive effects, promoting tumor growth and metastasis. Studies have demonstrated that these cells may serve as a potential contributing factor to the development and progression of prostate cancer.^[62]^

For the occurrence and development of cancer, the mechanism of the interaction between GM and immune cells is complex, and the GM can affect cancer through immune cells in a variety of ways, including metabolites, inflammatory markers, direct inhibition or promotion of immune response, and affecting the intestinal barrier.^[55,63,64]^ The GM is capable of metabolizing and generating metabolites like short-chain fatty acids, secondary bile acids, lactic acid, and bacteriocins. These metabolites can regulate the immune response by activating specific neurons and immune cells, thus maintaining the immune homeostasis of the intestinal mucosa.^[65]^ Some of the results of our study, such as *P bivia*, may be involved in the fermentation of dietary fibers and other substances to synthesize butyrate. Butyrate, serving as a crucial energy source for colon cells, exerts a major role in intestinal homeostasis.^[66]^ It can play a role in the development of human tumors by inhibiting histone deacetylase 3 and promoting the differentiation of monocytes into macrophages.^[67]^

Our study found that *M funiformis* can have an effect on breast cancer through CD38 on IgD+ CD24‐. This means that CD38 on IgD+ CD24‐ plays a key mediating role in the link between breast cancer and *M funiformis*. We performed mediation analyses to calculate the proportion of indirect effects. The mediating ratio of 11.20% suggests that these immune cells may play an important role in how these GM affect breast cancer. In addition, Species *Prevotellamassilia* also influenced the development of BC through HLA DR on CD33br HLA DR + CD14-. Studies have demonstrated that Species *M funiformis* is related to obesity, colon cancer, intestinal inflammation, and ankylosing spondylitis. However, the complex mechanism by which it acts on the human body remains unclear. We found that it can interact with CD38 on IgD+ CD24‐ cells, and then regulate the amount of CD38 on IgD+ CD24‐, thereby affecting the occurrence and development of the disease.^[68]^ Some studies have found that CD38 on IgD+ CD24‐ cells are more associated with breast cancer, lung cancer and other diseases. It can suppress the antitumor immune response by secreting certain immunosuppressive cytokines, such as IL-10 and transforming growth factor β. These cytokines possess the ability to inhibit the activity of T cells and attenuate their cytotoxic effect on tumor cells, consequently facilitating tumor growth and metastasis. Follicular B cells can also induce the production of regulatory T cells, further enhancing the immunosuppressive microenvironment, which favors tumor cells to evade immune surveillance, thereby aggravating the development of the disease.^[69]^ Species *Prevotellamassilia* is a common strain that is associated with allergies, rheumatoid arthritis, hypertension, multiple sclerosis and other diseases, and can interact with the gut and regulate the intestinal immune microenvironment. We found that it may be associated with HLA DR on CD33br HLA DR+ CD14‐ cells in the development of breast cancer.CD33br HLA DR+ CD14‐ cells might be a kind of cell possessing certain antigen-presenting ability. These cells could exert immunosuppressive effects, modulate the intensity of immune responses, and avert excessive immune responses from inflicting damage on the body by secreting inhibitory cytokines or directly interacting with other immune cells. The mechanism of action of these cells is rather complex. Some studies have shown that it also has a certain correlation with prostate cancer, and this cell population may regulate breast cancer through complex effects, and its specific mechanism needs to be further studied to prove.^[62]^

In this study, we employed the MR design to delve into the causal effect of the GM on breast cancer and also carried out research on the mediating role of immune cells in the connection between the GM and breast cancer. Within the realm of observational studies, this research can simulate randomized controlled trials at relatively low costs and with a minor risk of reverse causal influence. However, we still have some limitations. (1) Even though a variety of sensitivity analyses have been carried out to assess the hypotheses of Mendelian randomization studies, confounding bias, its potential heterogeneity, and horizontal pleiotropy cannot be entirely eradicated. (2) The majority of our data pertains to the European population. There could be a lack of broad applicability when considering other ethnic groups. The results might not hold true for all races and populations. Hence, it is necessary to undertake further validations to determine whether our research findings can be beneficial to Asian or other groups. (3) Breast cancer cases are gathered from public databases. The absence of individual-level data restricts our exploration of more complex relationships. In reality, these relationships may be even more intricate, involving environmental and other genetic factors. Moreover, it may lead to the neglect of nonlinear associations such as U-shaped or J-shaped associations between GM, immune cell traits and breast cancer. (4) We have merely reached conclusions at the theoretical level and have not been confirmed through clinical or animal experiments. Therefore, the specific mechanism remains unknown. (5) In GWAS of the GM, limited sample sizes might pose difficulties in adequately detecting potential causal relationships. To obtain statistically significant and accurate results, our studies demand larger sample sizes. Nevertheless, this study is currently the largest GWAS on the GM, and it possesses species-level data that can offer a more precise classification of GM.

## 5. Conclusion

Our study identified a potential causal relationship between GM, immune cells, and breast cancer by applying MR analysis. Specifically, *M funiformis* and Species *Prevotellamassilia* can affect breast cancer by CD38 on IgD+ CD24‐ and HLA DR on CD33br HLA DR+ CD14‐, respectively. These findings provide genetic evidence for a causal relationship between GM, immune cells, and breast cancer, highlighting the critical role of the GM in regulating immune responses and its potential importance in breast cancer. The identified associations and mediating effects have blazed a trail for subsequent research and might offer a reference for future mechanistic and clinical studies in this domain. To acquire a more elaborate understanding of the observed association between the GM and breast cancer, future research ought to concentrate on potential mechanistic pathways. Meanwhile, concerted efforts must be dedicated to conducting in-depth studies on the role of GM in modulating the immune response. Such endeavors could steer the development of targeted immunotherapy, furnish invaluable guidance for the prevention of breast cancer, and potentially facilitate early diagnosis and the formulation of more efficacious treatment plans.

## Author contributions

**Conceptualization:** Rui Lv, Tengyue Wang, Rongqun Li, Aiwen Zhuang.

**Data curation:** Rui Lv, Tengyue Wang, Rongqun Li, Aiwen Zhuang.

**Formal analysis:** Rui Lv, Danyan Wang, Tengyue Wang.

**Funding acquisition:** Aiwen Zhuang.

**Investigation:** Rui Lv, Danyan Wang, Tengyue Wang.

**Methodology:** Rui Lv, Danyan Wang, Tengyue Wang, Rongqun Li, Aiwen Zhuang.

**Project administration:** Rongqun Li, Aiwen Zhuang.

**Resources:** Rongqun Li, Aiwen Zhuang.

**Supervision:** Rongqun Li, Aiwen Zhuang.

**Visualization:** Rui Lv, Danyan Wang, Tengyue Wang.

**Writing – original draft:** Rui Lv, Danyan Wang, Tengyue Wang.

**Writing – review & editing:** Rongqun Li, Aiwen Zhuang.

## Supplementary Material



## References

[1] Kawiak A. Molecular research and treatment of breast cancer. Int J Mol Sci. 2022;23:9617. 36077013 10.3390/ijms23179617 PMC9455640

[2] Idossa D Borrero M Blaes A. ERBB2-low (also known as HER2-low) breast cancer. JAMA Oncol. 2023;9:576. 36821135 10.1001/jamaoncol.2022.6889

[3] Britt KL Cuzick J Phillips KA. Key steps for effective breast cancer prevention. Nat Rev Cancer. 2020;20:417–36. 32528185 10.1038/s41568-020-0266-x

[4] Chen J Douglass J Prasath V. The microbiome and breast cancer: a review. Breast Cancer Res Treat. 2019;178:493–6. 31456069 10.1007/s10549-019-05407-5

[5] Qin J Li R Raes J. A human gut microbial gene catalogue established by metagenomic sequencing. Nature. 2010;464:59–65. 20203603 10.1038/nature08821 PMC3779803

[6] Schluter J Peled JU Taylor BP. The gut microbiota is associated with immune cell dynamics in humans. Nature. 2020;588:303–7. 33239790 10.1038/s41586-020-2971-8 PMC7725892

[7] de Martel C Ferlay J Franceschi S. Global burden of cancers attributable to infections in 2008: a review and synthetic analysis. Lancet Oncol. 2012;13:607–15. 22575588 10.1016/S1470-2045(12)70137-7

[8] Fernández MF Reina-Pérez I Astorga JM Rodríguez-Carrillo A Plaza-Díaz J Fontana L. Breast cancer and its relationship with the microbiota. Int J Environ Res Public Health. 2018;15:1747. 30110974 10.3390/ijerph15081747 PMC6121903

[9] Walker MM Talley NJ. Review article: bacteria and pathogenesis of disease in the upper gastrointestinal tract – beyond the era of *Helicobacter pylori*. Aliment Pharmacol Ther. 2014;39:767–79. 24612362 10.1111/apt.12666

[10] Parida S Sharma D. The power of small changes: comprehensive analyses of microbial dysbiosis in breast cancer. Biochim Biophys Acta Rev Cancer. 2019;1871:392–405. 30981803 10.1016/j.bbcan.2019.04.001 PMC8769497

[11] Parida S Sharma D. Microbial alterations and risk factors of breast cancer: connections and mechanistic insights. Cells. 2020;9:1091. 32354130 10.3390/cells9051091 PMC7290701

[12] Tikkanen MJ Adlercreutz H Pulkkinen MO. Effects of antibiotics on oestrogen metabolism. Br Med J. 1973;2:369. 10.1136/bmj.2.5862.369 PMC1589303 4704532

[13] Velicer CM Lampe JW Heckbert SR Potter JD Taplin SH. Hypothesis: is antibiotic use associated with breast cancer? Cancer Causes Control. 2003;14:739–47. 14674738 10.1023/a:1026323424792

[14] Gopalakrishnan V Spencer CN Nezi L. Gut microbiome modulates response to anti-PD-1 immunotherapy in melanoma patients. Science. 2018;359:97–103. 29097493 10.1126/science.aan4236 PMC5827966

[15] Monnot GC Romero P. Rationale for immunological approaches to breast cancer therapy. Breast. 2018;37:187–95. 28629632 10.1016/j.breast.2017.06.009

[16] Corthay A. Does the immune system naturally protect against cancer? Front Immunol. 2014;5:197. 24860567 10.3389/fimmu.2014.00197 PMC4026755

[17] Alspach E Lussier DM Schreiber RD. Interferon γ and its important roles in promoting and inhibiting spontaneous and therapeutic cancer immunity. Cold Spring Harb Perspect Biol. 2019;11:a028480. 29661791 10.1101/cshperspect.a028480 PMC6396335

[18] Alizadehmohajer N Shojaeifar S Nedaeinia R. Association between the microbiota and women’s cancers – cause or consequences? Biomed Pharmacother. 2020;127:110203. 32559847 10.1016/j.biopha.2020.110203

[19] Hwang SW Kim MK Kweon MN. Gut microbiome on immune checkpoint inhibitor therapy and consequent immune-related colitis: a review. Intest Res. 2023;21:433–42. 37640378 10.5217/ir.2023.00019 PMC10626011

[20] Emdin CA Khera AV Kathiresan S. Mendelian randomization. JAMA. 2017;318:1925–6. 29164242 10.1001/jama.2017.17219

[21] Bowden J Davey Smith G Burgess S. Mendelian randomization with invalid instruments: effect estimation and bias detection through Egger regression. Int J Epidemiol. 2015;44:512–25. 26050253 10.1093/ije/dyv080 PMC4469799

[22] Davies NM Holmes MV Davey Smith G. Reading Mendelian randomisation studies: a guide, glossary, and checklist for clinicians. BMJ. 2018;362:k601. 30002074 10.1136/bmj.k601 PMC6041728

[23] Qin Y Havulinna AS Liu Y. Combined effects of host genetics and diet on human gut microbiota and incident disease in a single population cohort [published correction appears in Nat Genet. 2024 Mar;56(3):554. doi: 10.1038/s41588-024-01693-y]. Nat Genet. 2022;54:134–42. 35115689 10.1038/s41588-021-00991-z PMC9883041

[24] Orrù V Steri M Sidore C. Complex genetic signatures in immune cells underlie autoimmunity and inform therapy [published correction appears in Nat Genet. 2020 Nov;52(11):1266. doi: 10.1038/s41588-020-00718-6]. Nat Genet. 2020;52:1036–45. 32929287 10.1038/s41588-020-0684-4 PMC8517961

[25] Wang X Gao H Zeng Y Chen J. A Mendelian analysis of the relationships between immune cells and breast cancer. Front Oncol. 2024;14:1341292. 38327747 10.3389/fonc.2024.1341292 PMC10847340

[26] Sakaue S Kanai M Tanigawa Y. A cross-population atlas of genetic associations for 220 human phenotypes. Nat Genet. 2021;53:1415–24. 34594039 10.1038/s41588-021-00931-x PMC12208603

[27] Wang C Zhu D Zhang D. Causal role of immune cells in schizophrenia: Mendelian randomization (MR) study. BMC Psychiatry. 2023;23:590. 37582716 10.1186/s12888-023-05081-4 PMC10428653

[28] Slatkin M. Linkage disequilibrium – understanding the evolutionary past and mapping the medical future. Nat Rev Genet. 2008;9:477–85. 18427557 10.1038/nrg2361 PMC5124487

[29] Palmer TM Lawlor DA Harbord RM. Using multiple genetic variants as instrumental variables for modifiable risk factors. Stat Methods Med Res. 2012;21:223–42. 21216802 10.1177/0962280210394459 PMC3917707

[30] Ziegler A Pahlke F König IR. Comments on “Mendelian randomization: using genes as instruments for making causal inferences in epidemiology” by Debbie A. Lawlor, R. M. Harbord, J. A. Sterne, N. Timpson and G. Davey Smith, Statistics in Medicine, DOI: 10.1002/sim.3034. Stat Med. 2008;27:2974–6; author reply 2976. 18203119 10.1002/sim.3213

[31] Burgess S Butterworth A Thompson SG. Mendelian randomization analysis with multiple genetic variants using summarized data. Genet Epidemiol. 2013;37:658–65. 24114802 10.1002/gepi.21758 PMC4377079

[32] Bowden J Davey Smith G Haycock PC Burgess S. Consistent estimation in Mendelian randomization with some invalid instruments using a weighted median estimator. Genet Epidemiol. 2016;40:304–14. 27061298 10.1002/gepi.21965 PMC4849733

[33] Hartwig FP Davey Smith G Bowden J. Robust inference in summary data Mendelian randomization via the zero modal pleiotropy assumption. Int J Epidemiol. 2017;46:1985–98. 29040600 10.1093/ije/dyx102 PMC5837715

[34] Zhang Y Liu Z Choudhury T Cornelis MC Liu W. Habitual coffee intake and risk for nonalcoholic fatty liver disease: a two-sample Mendelian randomization study. Eur J Nutr. 2021;60:1761–7. 32856188 10.1007/s00394-020-02369-z PMC7910323

[35] Verbanck M Chen CY Neale B Do R. Detection of widespread horizontal pleiotropy in causal relationships inferred from Mendelian randomization between complex traits and diseases [published correction appears in Nat Genet. 2018 Aug;50(8):1196. doi: 10.1038/s41588-018-0164-2]. Nat Genet. 2018;50:693–8. 29686387 10.1038/s41588-018-0099-7 PMC6083837

[36] Carter AR Sanderson E Hammerton G. Mendelian randomisation for mediation analysis: current methods and challenges for implementation. Eur J Epidemiol. 2021;36:465–78. 33961203 10.1007/s10654-021-00757-1 PMC8159796

[37] Bobin-Dubigeon C Luu HT Leuillet S. Faecal microbiota composition varies between patients with breast cancer and healthy women: a comparative case–control study. Nutrients. 2021;13:2705. 34444865 10.3390/nu13082705 PMC8399700

[38] Xuan C Shamonki JM Chung A. Microbial dysbiosis is associated with human breast cancer. PLoS One. 2014;9:e83744. 24421902 10.1371/journal.pone.0083744 PMC3885448

[39] Luu TH Michel C Bard JM Dravet F Nazih H Bobin-Dubigeon C. Intestinal proportion of Blautia sp. is associated with clinical stage and histoprognostic grade in patients with early-stage breast cancer. Nutr Cancer. 2017;69:267–75. 28094541 10.1080/01635581.2017.1263750

[40] Gloux K Berteau O El Oumami H Béguet F Leclerc M Doré J. A metagenomic β-glucuronidase uncovers a core adaptive function of the human intestinal microbiome. Proc Natl Acad Sci U S A. 2011;108(Suppl 1):4539–46. 20615998 10.1073/pnas.1000066107 PMC3063586

[41] McIntosh FM Maison N Holtrop G. Phylogenetic distribution of genes encoding β-glucuronidase activity in human colonic bacteria and the impact of diet on faecal glycosidase activities. Environ Microbiol. 2012;14:1876–87. 22364273 10.1111/j.1462-2920.2012.02711.x

[42] Kostic AD Gevers D Pedamallu CS. Genomic analysis identifies association of Fusobacterium with colorectal carcinoma. Genome Res. 2012;22:292–8. 22009990 10.1101/gr.126573.111 PMC3266036

[43] Mazmanian SK Round JL Kasper DL. A microbial symbiosis factor prevents intestinal inflammatory disease. Nature. 2008;453:620–5. 18509436 10.1038/nature07008

[44] Malavasi F Deaglio S Funaro A. Evolution and function of the ADP ribosyl cyclase/CD38 gene family in physiology and pathology. Physiol Rev. 2008;88:841–86. 18626062 10.1152/physrev.00035.2007

[45] Morandi F Airoldi I Marimpietri D Bracci C Faini AC Gramignoli R. CD38, a receptor with multifunctional activities: from modulatory functions on regulatory cell subsets and extracellular vesicles, to a target for therapeutic strategies. Cells. 2019;8:1527. 31783629 10.3390/cells8121527 PMC6953043

[46] Liao S Xiao S Zhu G. CD38 is highly expressed and affects the PI3K/Akt signaling pathway in cervical cancer. Oncol Rep. 2014;32:2703–9. 25310288 10.3892/or.2014.3537

[47] Levy A Blacher E Vaknine H Lund FE Stein R Mayo L. CD38 deficiency in the tumor microenvironment attenuates glioma progression and modulates features of tumor-associated microglia/macrophages. Neuro Oncol. 2012;14:1037–49. 22700727 10.1093/neuonc/nos121 PMC3408254

[48] Karakasheva TA Waldron TJ Eruslanov E. CD38-expressing myeloid-derived suppressor cells promote tumor growth in a murine model of esophageal cancer. Cancer Res. 2015;75:4074–85. 26294209 10.1158/0008-5472.CAN-14-3639 PMC4592477

[49] Ben Baruch B Blacher E Mantsur E. Stromal CD38 regulates outgrowth of primary melanoma and generation of spontaneous metastasis. Oncotarget. 2018;9:31797–811. 30159123 10.18632/oncotarget.25737 PMC6112753

[50] Bu X Kato J Hong JA. CD38 knockout suppresses tumorigenesis in mice and clonogenic growth of human lung cancer cells. Carcinogenesis. 2018;39:242–51. 29228209 10.1093/carcin/bgx137 PMC5862338

[51] van de Donk NW Janmaat ML Mutis T. Monoclonal antibodies targeting CD38 in hematological malignancies and beyond. Immunol Rev. 2016;270:95–112. 26864107 10.1111/imr.12389 PMC4755228

[52] Deaglio S Vaisitti T Aydin S. CD38 and ZAP-70 are functionally linked and mark CLL cells with high migratory potential. Blood. 2007;110:4012–21. 17699742 10.1182/blood-2007-06-094029

[53] Chen L Diao L Yang Y. CD38-mediated immunosuppression as a mechanism of tumor cell escape from PD-1/PD-L1 blockade. Cancer Discov. 2018;8:1156–75. 30012853 10.1158/2159-8290.CD-17-1033 PMC6205194

[54] Schwabe RF Jobin C. The microbiome and cancer. Nat Rev Cancer. 2013;13:800–12. 24132111 10.1038/nrc3610 PMC3986062

[55] Yu LX Schwabe RF. The gut microbiome and liver cancer: mechanisms and clinical translation. Nat Rev Gastroenterol Hepatol. 2017;14:527–39. 28676707 10.1038/nrgastro.2017.72 PMC6467288

[56] Ruffell B Au A Rugo HS Esserman LJ Hwang ES Coussens LM. Leukocyte composition of human breast cancer. Proc Natl Acad Sci U S A. 2012;109:2796–801. 21825174 10.1073/pnas.1104303108 PMC3287000

[57] Wang Y Xu B Hu WW. High expression of CD11c indicates favorable prognosis in patients with gastric cancer. World J Gastroenterol. 2015;21:9403–12. 26309367 10.3748/wjg.v21.i31.9403 PMC4541393

[58] Thaiss CA Semmling V Franken L Wagner H Kurts C. Chemokines: a new dendritic cell signal for T cell activation. Front Immunol. 2011;2:31. 22566821 10.3389/fimmu.2011.00031 PMC3342358

[59] Ma A Koka R Burkett P. Diverse functions of IL-2, IL-15, and IL-7 in lymphoid homeostasis. Annu Rev Immunol. 2006;24:657–79. 16551262 10.1146/annurev.immunol.24.021605.090727

[60] Al-Rawi MA Rmali K Mansel RE Jiang WG. Interleukin 7 induces the growth of breast cancer cells through a wortmannin-sensitive pathway. Br J Surg. 2004;91:61–8. 14716795 10.1002/bjs.4449

[61] Kubo M Satoh T Ishiyama H. Enhanced activated T cell subsets in prostate cancer patients receiving iodine-125 low-dose-rate prostate brachytherapy. Oncol Rep. 2018;39:417–24. 29138841 10.3892/or.2017.6095

[62] Zhong M Zhan X Zhong FP. Causal role of immune cells in prostate cancer: a bidirectional Mendelian-randomization analyses. Aging (Albany NY). 2024;16:10477–88. 38888513 10.18632/aging.205942 PMC11236311

[63] Thiele Orberg E Fan H Tam AJ. The myeloid immune signature of enterotoxigenic *Bacteroides fragilis*-induced murine colon tumorigenesis. Mucosal Immunol. 2017;10:421–33. 27301879 10.1038/mi.2016.53 PMC5159334

[64] Vétizou M Pitt JM Daillère R. Anticancer immunotherapy by CTLA-4 blockade relies on the gut microbiota. Science. 2015;350:1079–84. 26541610 10.1126/science.aad1329 PMC4721659

[65] de Sablet T Chassard C Bernalier-Donadille A Vareille M Gobert AP Martin C. Human microbiota-secreted factors inhibit shiga toxin synthesis by enterohemorrhagic *Escherichia coli* O157:H7. Infect Immun. 2009;77:783–90. 19064636 10.1128/IAI.01048-08 PMC2632037

[66] Yoo JY Groer M Dutra SVO Sarkar A McSkimming DI. Gut microbiota and immune system interactions [published correction appears in Microorganisms. 2020 Dec 21;8(12):E2046. doi: 10.3390/microorganisms8122046]. Microorganisms. 2020;8:1587. 33371530 10.3390/microorganisms8122046 PMC7765795

[67] Schulthess J Pandey S Capitani M. The short chain fatty acid butyrate imprints an antimicrobial program in macrophages. Immunity. 2019;50:432–45.e7. 30683619 10.1016/j.immuni.2018.12.018 PMC6382411

[68] Yang X Zhang M Liu Y. Inulin-enriched *Megamonas funiformis* ameliorates metabolic dysfunction-associated fatty liver disease by producing propionic acid [published correction appears in NPJ Biofilms Microbiomes. 2024 Jan 29;10(1):9. doi: 10.1038/s41522-024-00480-1]. npj Biofilms Microbiomes. 2023;9:84. 37925493 10.1038/s41522-023-00451-y PMC10625582

[69] Germain C Gnjatic S Tamzalit F. Presence of B cells in tertiary lymphoid structures is associated with a protective immunity in patients with lung cancer. Am J Respir Crit Care Med. 2014;189:832–44. 24484236 10.1164/rccm.201309-1611OC

