# Supplementary material for: Causality between gut microbiota, immune cells, and breast cancer: Mendelian randomization analysis
Source: Medicine (Baltimore). 2024 Dec 6;103(49):e40815. doi: 10.1097/MD.0000000000040815 (PMC11630993; doi:10.1097/MD.0000000000040815)

| **Table S1.** Overview of data | | | | | | | | | | |  |  |  |  |  |  |  |  |  |  |  |  |  |  |  |
| --- | --- | --- | --- | --- | --- | --- | --- | --- | --- | --- | --- | --- | --- | --- | --- | --- | --- | --- | --- | --- | --- | --- | --- | --- | --- |
|  |  |  |  |  |  |  |  |  |  |  |  |  |  |  |  |  |  |  |  |  |  |  |  |  |  |
| **Phenotype** | **Consortium** | **Population** | **n case** | **n control** | **Link** | **Accession number** |  |  |  |  |  |  |  |  |  |  |  |  |  |  |  |  |  |  |  |
| 473 gut microbiota | doi: 10.1038/s41588-021-00991-z | European | 5959 |  | https://gwas.mrcieu.ac.uk/datasets/ | ebi-a-GCST90016908 to ebi-a-GCST90017118 | |  |  |  |  |  |  |  |  |  |  |  |  |  |  |  |  |  |  |
|  |  |  |  |  |  |  |  |  |  |  |  |  |  |  |  |  |  |  |  |  |  |  |  |  |  |
| 731 immune cells traits | doi: 10.1038/s41588-020-0684-4 | European | over 3000 |  | https://gwas.mrcieu.ac.uk/datasets/ | ebi-a-GCST90001391 to ebi-a-GCST90002121 | |  |  |  |  |  |  |  |  |  |  |  |  |  |  |  |  |  |  |
|  |  |  |  |  |  |  |  |  |  |  |  |  |  |  |  |  |  |  |  |  |  |  |  |  |  |
| breast cancer | doi:10.1038/s41588-021-00931-x | European | 17389 | 240341 | https://gwas.mrcieu.ac.uk/datasets/ |  |  |  |  |  |  |  |  |  |  |  |  |  |  |  |  |  |  |  |  |

| **Table S2.** SNP for significant MR analysis | | | | | |
| --- | --- | --- | --- | --- | --- |
|  |  |  |  |  |  |
| **SNP for MR analysis between Gut microbiota and Breast cancer** | | | | | |
| Exposure | Outcome | SNP | Beta | Se | Pvalue |
| Phylum Actinobacteriota | Breast Cancer | rs10440729 | 0.0294462 | 0.00661194 | 8.40E-06 |
| Phylum Actinobacteriota | Breast Cancer | rs112134915 | 0.0694174 | 0.0153756 | 6.30E-06 |
| Phylum Actinobacteriota | Breast Cancer | rs1890115 | -0.0318961 | 0.00708692 | 6.80E-06 |
| Phylum Actinobacteriota | Breast Cancer | rs2164706 | 0.0311264 | 0.00696623 | 7.90E-06 |
| Phylum Actinobacteriota | Breast Cancer | rs2250301 | -0.0229346 | 0.00516973 | 9.20E-06 |
| Phylum Actinobacteriota | Breast Cancer | rs2983215 | -0.0254038 | 0.0055947 | 5.60E-06 |
| Phylum Actinobacteriota | Breast Cancer | rs35307665 | -0.031698 | 0.00670697 | 2.30E-06 |
| Phylum Actinobacteriota | Breast Cancer | rs3940549 | 0.050729 | 0.00513697 | 5.30E-23 |
| Phylum Actinobacteriota | Breast Cancer | rs62484243 | 0.0400151 | 0.00848481 | 2.40E-06 |
| Phylum Actinobacteriota | Breast Cancer | rs6492094 | 0.0246117 | 0.0055456 | 9.10E-06 |
| Phylum Actinobacteriota | Breast Cancer | rs73101198 | 0.0532742 | 0.01196 | 8.40E-06 |
| Phylum Actinobacteriota | Breast Cancer | rs771997 | -0.0250611 | 0.00558648 | 7.30E-06 |
| Phylum Actinobacteriota | Breast Cancer | rs797815 | -0.0273506 | 0.00601988 | 5.50E-06 |
| Genus An7 | Breast Cancer | rs1016579 | 0.0817956 | 0.0181092 | 6.30E-06 |
| Genus An7 | Breast Cancer | rs118162561 | 0.0961169 | 0.020823 | 3.90E-06 |
| Genus An7 | Breast Cancer | rs139195374 | 0.0655593 | 0.0146199 | 7.30E-06 |
| Genus An7 | Breast Cancer | rs143347000 | 0.0574873 | 0.012811 | 7.20E-06 |
| Genus An7 | Breast Cancer | rs145171932 | 0.123477 | 0.0225103 | 4.10E-08 |
| Genus An7 | Breast Cancer | rs17258709 | -0.0480784 | 0.0105296 | 5.00E-06 |
| Genus An7 | Breast Cancer | rs2283177 | 0.0714829 | 0.0155977 | 4.60E-06 |
| Genus An7 | Breast Cancer | rs2797887 | 0.119365 | 0.0265818 | 7.10E-06 |
| Genus An7 | Breast Cancer | rs2852623 | -0.0556296 | 0.0111079 | 5.50E-07 |
| Genus An7 | Breast Cancer | rs3911603 | -0.0438764 | 0.00992335 | 9.80E-06 |
| Genus An7 | Breast Cancer | rs4674463 | -0.0710934 | 0.0143534 | 7.30E-07 |
| Genus An7 | Breast Cancer | rs4722327 | 0.034588 | 0.00738657 | 2.80E-06 |
| Genus An7 | Breast Cancer | rs61742849 | 0.137142 | 0.0267657 | 3.00E-07 |
| Genus An7 | Breast Cancer | rs7043204 | 0.0414647 | 0.00909919 | 5.20E-06 |
| Genus An7 | Breast Cancer | rs76928381 | 0.153177 | 0.0290407 | 1.30E-07 |
| Species Bacteroides A plebeius A | Breast Cancer | rs10510731 | 0.112354 | 0.0224698 | 5.70E-07 |
| Species Bacteroides A plebeius A | Breast Cancer | rs11193092 | 0.239782 | 0.0539899 | 8.90E-06 |
| Species Bacteroides A plebeius A | Breast Cancer | rs12314696 | 0.195811 | 0.0431981 | 5.80E-06 |
| Species Bacteroides A plebeius A | Breast Cancer | rs12436076 | -0.121437 | 0.0267624 | 5.70E-06 |
| Species Bacteroides A plebeius A | Breast Cancer | rs12885338 | -0.0843342 | 0.0189146 | 8.20E-06 |
| Species Bacteroides A plebeius A | Breast Cancer | rs139389953 | 0.466232 | 0.0826217 | 1.70E-08 |
| Species Bacteroides A plebeius A | Breast Cancer | rs145791346 | 0.309116 | 0.069153 | 7.80E-06 |
| Species Bacteroides A plebeius A | Breast Cancer | rs149561196 | 0.273343 | 0.0562642 | 1.20E-06 |
| Species Bacteroides A plebeius A | Breast Cancer | rs34760623 | 0.245941 | 0.0513769 | 1.70E-06 |
| Species Bacteroides A plebeius A | Breast Cancer | rs3748487 | 0.236002 | 0.0520699 | 5.80E-06 |
| Species Bacteroides A plebeius A | Breast Cancer | rs3754935 | 0.169694 | 0.0377568 | 7.00E-06 |
| Species Bacteroides A plebeius A | Breast Cancer | rs57197355 | -0.123645 | 0.0272504 | 5.70E-06 |
| Species Bacteroides A plebeius A | Breast Cancer | rs61269211 | 0.125397 | 0.0257311 | 1.10E-06 |
| Species Bacteroides A plebeius A | Breast Cancer | rs61881773 | 0.254248 | 0.054798 | 3.50E-06 |
| Species Bacteroides A plebeius A | Breast Cancer | rs61912384 | 0.112245 | 0.024946 | 6.80E-06 |
| Species Bacteroides A plebeius A | Breast Cancer | rs7179783 | 0.170501 | 0.0378988 | 6.80E-06 |
| Species Bacteroides A plebeius A | Breast Cancer | rs73113075 | -0.243542 | 0.054858 | 9.00E-06 |
| Species Bacteroides A plebeius A | Breast Cancer | rs73594223 | 0.28694 | 0.0596332 | 1.50E-06 |
| Species Bacteroides A plebeius A | Breast Cancer | rs77969904 | 0.274313 | 0.0590701 | 3.40E-06 |
| Species Bacteroides A plebeius A | Breast Cancer | rs7927877 | 0.0959895 | 0.020696 | 3.50E-06 |
| Species Bacteroides A plebeius A | Breast Cancer | rs9759894 | 0.100905 | 0.0216277 | 3.10E-06 |
| Species Bifidobacterium adolescentis | Breast Cancer | rs10055404 | -0.101644 | 0.0227161 | 7.70E-06 |
| Species Bifidobacterium adolescentis | Breast Cancer | rs113861295 | 0.222257 | 0.0497553 | 7.90E-06 |
| Species Bifidobacterium adolescentis | Breast Cancer | rs114319347 | -0.469817 | 0.0994123 | 2.30E-06 |
| Species Bifidobacterium adolescentis | Breast Cancer | rs11672816 | -0.141269 | 0.0315614 | 7.60E-06 |
| Species Bifidobacterium adolescentis | Breast Cancer | rs12611001 | -0.263923 | 0.0546761 | 1.40E-06 |
| Species Bifidobacterium adolescentis | Breast Cancer | rs139688845 | 0.216822 | 0.0477455 | 5.60E-06 |
| Species Bifidobacterium adolescentis | Breast Cancer | rs140200601 | 0.113001 | 0.0242159 | 3.10E-06 |
| Species Bifidobacterium adolescentis | Breast Cancer | rs1511397 | -0.0992589 | 0.0222703 | 8.30E-06 |
| Species Bifidobacterium adolescentis | Breast Cancer | rs170149 | -0.273959 | 0.0607642 | 6.50E-06 |
| Species Bifidobacterium adolescentis | Breast Cancer | rs17092955 | -0.165315 | 0.0339814 | 1.10E-06 |
| Species Bifidobacterium adolescentis | Breast Cancer | rs17849163 | 0.120378 | 0.0251932 | 1.80E-06 |
| Species Bifidobacterium adolescentis | Breast Cancer | rs17881456 | -0.207193 | 0.0455853 | 5.50E-06 |
| Species Bifidobacterium adolescentis | Breast Cancer | rs182549 | 0.206968 | 0.0224734 | 3.30E-20 |
| Species Bifidobacterium adolescentis | Breast Cancer | rs2836338 | -0.0999606 | 0.0220739 | 5.90E-06 |
| Species Bifidobacterium adolescentis | Breast Cancer | rs335752 | 0.130423 | 0.0279642 | 3.10E-06 |
| Species Bifidobacterium adolescentis | Breast Cancer | rs55892303 | 0.125537 | 0.0262787 | 1.80E-06 |
| Species Bifidobacterium adolescentis | Breast Cancer | rs56098739 | 0.123291 | 0.0244984 | 4.80E-07 |
| Species Bifidobacterium adolescentis | Breast Cancer | rs6768653 | -0.195529 | 0.0426163 | 4.50E-06 |
| Species Bifidobacterium adolescentis | Breast Cancer | rs716064 | 0.10581 | 0.0234062 | 6.20E-06 |
| Species Bifidobacterium adolescentis | Breast Cancer | rs78686363 | -0.227139 | 0.0493098 | 4.10E-06 |
| Species Bifidobacterium adolescentis | Breast Cancer | rs79931074 | 0.213489 | 0.0463538 | 4.10E-06 |
| Species Bifidobacterium adolescentis | Breast Cancer | rs9520255 | -0.111115 | 0.0234837 | 2.20E-06 |
| Species Blautia sp001304935 | Breast Cancer | rs10497521 | -0.0718947 | 0.0148629 | 1.30E-06 |
| Species Blautia sp001304935 | Breast Cancer | rs114081723 | 0.180116 | 0.0317909 | 1.50E-08 |
| Species Blautia sp001304935 | Breast Cancer | rs115707269 | 0.168265 | 0.0373666 | 6.70E-06 |
| Species Blautia sp001304935 | Breast Cancer | rs117918530 | 0.119673 | 0.0270317 | 9.60E-06 |
| Species Blautia sp001304935 | Breast Cancer | rs12183541 | 0.0761242 | 0.0161782 | 2.50E-06 |
| Species Blautia sp001304935 | Breast Cancer | rs13274856 | 0.10852 | 0.0216775 | 5.60E-07 |
| Species Blautia sp001304935 | Breast Cancer | rs144583916 | 0.153229 | 0.0333708 | 4.40E-06 |
| Species Blautia sp001304935 | Breast Cancer | rs145847454 | 0.0585718 | 0.0131 | 7.80E-06 |
| Species Blautia sp001304935 | Breast Cancer | rs147296376 | 0.177493 | 0.0344684 | 2.60E-07 |
| Species Blautia sp001304935 | Breast Cancer | rs2224153 | -0.0478212 | 0.00999479 | 1.70E-06 |
| Species Blautia sp001304935 | Breast Cancer | rs26147 | 0.0436257 | 0.00947517 | 4.10E-06 |
| Species Blautia sp001304935 | Breast Cancer | rs2678796 | -0.0951989 | 0.019855 | 1.60E-06 |
| Species Blautia sp001304935 | Breast Cancer | rs7227267 | 0.0461591 | 0.00961494 | 1.60E-06 |
| Species Blautia sp001304935 | Breast Cancer | rs72834033 | 0.151712 | 0.0286251 | 1.20E-07 |
| Species Blautia sp001304935 | Breast Cancer | rs763475 | 0.07009 | 0.0150079 | 3.00E-06 |
| Species Blautia sp001304935 | Breast Cancer | rs78943972 | 0.13834 | 0.0303478 | 5.20E-06 |
| Species CAG-180 sp000432435 | Breast Cancer | rs10495553 | 0.0803598 | 0.0180744 | 8.70E-06 |
| Species CAG-180 sp000432435 | Breast Cancer | rs11025048 | -0.091333 | 0.0189275 | 1.40E-06 |
| Species CAG-180 sp000432435 | Breast Cancer | rs117308453 | -0.16097 | 0.0352892 | 5.10E-06 |
| Species CAG-180 sp000432435 | Breast Cancer | rs118021689 | -0.123948 | 0.0276586 | 7.40E-06 |
| Species CAG-180 sp000432435 | Breast Cancer | rs143664615 | -0.0705877 | 0.015975 | 9.90E-06 |
| Species CAG-180 sp000432435 | Breast Cancer | rs146036661 | -0.214617 | 0.0464503 | 3.80E-06 |
| Species CAG-180 sp000432435 | Breast Cancer | rs147678928 | -0.208215 | 0.0425086 | 9.70E-07 |
| Species CAG-180 sp000432435 | Breast Cancer | rs150768243 | -0.232293 | 0.0520998 | 8.20E-06 |
| Species CAG-180 sp000432435 | Breast Cancer | rs2483001 | -0.0717234 | 0.0131167 | 4.50E-08 |
| Species CAG-180 sp000432435 | Breast Cancer | rs34967948 | 0.0631729 | 0.0140288 | 6.70E-06 |
| Species CAG-180 sp000432435 | Breast Cancer | rs4886473 | -0.0550531 | 0.0121963 | 6.40E-06 |
| Species CAG-180 sp000432435 | Breast Cancer | rs6044205 | -0.133547 | 0.0271549 | 8.70E-07 |
| Species CAG-180 sp000432435 | Breast Cancer | rs61132499 | -0.206648 | 0.0464643 | 8.70E-06 |
| Species CAG-180 sp000432435 | Breast Cancer | rs61516561 | 0.234085 | 0.050625 | 3.80E-06 |
| Species CAG-180 sp000432435 | Breast Cancer | rs61852604 | -0.134955 | 0.0287918 | 2.80E-06 |
| Species CAG-180 sp000432435 | Breast Cancer | rs71478860 | 0.0713731 | 0.0161101 | 9.40E-06 |
| Species CAG-180 sp000432435 | Breast Cancer | rs7255963 | 0.0560613 | 0.0125684 | 8.20E-06 |
| Species CAG-180 sp000432435 | Breast Cancer | rs72766877 | -0.0722325 | 0.0160863 | 7.10E-06 |
| Species CAG-841 sp002479075 | Breast Cancer | rs10496950 | -0.128266 | 0.0286111 | 7.40E-06 |
| Species CAG-841 sp002479075 | Breast Cancer | rs10930336 | -0.137781 | 0.0305013 | 6.30E-06 |
| Species CAG-841 sp002479075 | Breast Cancer | rs11057029 | 0.135177 | 0.0266785 | 4.00E-07 |
| Species CAG-841 sp002479075 | Breast Cancer | rs111717438 | 0.12414 | 0.0269076 | 4.00E-06 |
| Species CAG-841 sp002479075 | Breast Cancer | rs11681167 | 0.127692 | 0.0247108 | 2.40E-07 |
| Species CAG-841 sp002479075 | Breast Cancer | rs12004677 | 0.162525 | 0.036736 | 9.70E-06 |
| Species CAG-841 sp002479075 | Breast Cancer | rs148911923 | 0.600151 | 0.128504 | 3.00E-06 |
| Species CAG-841 sp002479075 | Breast Cancer | rs2073454 | -0.11128 | 0.0251628 | 9.80E-06 |
| Species CAG-841 sp002479075 | Breast Cancer | rs278576 | -0.132723 | 0.0262572 | 4.30E-07 |
| Species CAG-841 sp002479075 | Breast Cancer | rs6871853 | -0.14048 | 0.0307812 | 5.00E-06 |
| Species CAG-841 sp002479075 | Breast Cancer | rs7445912 | 0.165662 | 0.0371766 | 8.30E-06 |
| Species CAG-841 sp002479075 | Breast Cancer | rs74582725 | 0.220783 | 0.0487532 | 5.90E-06 |
| Species CAG-841 sp002479075 | Breast Cancer | rs7599804 | 0.20293 | 0.0419521 | 1.30E-06 |
| Species CAG-841 sp002479075 | Breast Cancer | rs78279385 | 0.158067 | 0.0284821 | 2.90E-08 |
| Species CAG-841 sp002479075 | Breast Cancer | rs79338453 | -0.237214 | 0.0503448 | 2.50E-06 |
| Species CAG-841 sp002479075 | Breast Cancer | rs79834276 | -0.249813 | 0.0524976 | 1.90E-06 |
| Species CAG-841 sp002479075 | Breast Cancer | rs967895 | 0.121929 | 0.0264269 | 4.00E-06 |
| Species CHKCI006 sp900018345 | Breast Cancer | rs117019146 | -0.0997299 | 0.0217383 | 4.50E-06 |
| Species CHKCI006 sp900018345 | Breast Cancer | rs140230273 | 0.0756622 | 0.0164939 | 4.50E-06 |
| Species CHKCI006 sp900018345 | Breast Cancer | rs142316427 | -0.115406 | 0.0261051 | 9.80E-06 |
| Species CHKCI006 sp900018345 | Breast Cancer | rs146266330 | 0.125742 | 0.0263629 | 1.80E-06 |
| Species CHKCI006 sp900018345 | Breast Cancer | rs17479671 | 0.0490554 | 0.00927035 | 1.20E-07 |
| Species CHKCI006 sp900018345 | Breast Cancer | rs1921533 | -0.0684652 | 0.0153417 | 8.10E-06 |
| Species CHKCI006 sp900018345 | Breast Cancer | rs2032232 | 0.042094 | 0.00941497 | 7.80E-06 |
| Species CHKCI006 sp900018345 | Breast Cancer | rs2406603 | 0.1727 | 0.0389347 | 9.20E-06 |
| Species CHKCI006 sp900018345 | Breast Cancer | rs34336094 | 0.166622 | 0.0349401 | 1.90E-06 |
| Species CHKCI006 sp900018345 | Breast Cancer | rs34783347 | 0.0658597 | 0.0141303 | 3.10E-06 |
| Species CHKCI006 sp900018345 | Breast Cancer | rs3744783 | 0.0838475 | 0.0179288 | 2.90E-06 |
| Species CHKCI006 sp900018345 | Breast Cancer | rs4246167 | 0.0456398 | 0.0100968 | 6.20E-06 |
| Species CHKCI006 sp900018345 | Breast Cancer | rs4575760 | -0.046753 | 0.00963759 | 1.20E-06 |
| Species CHKCI006 sp900018345 | Breast Cancer | rs4857937 | 0.0451478 | 0.00939838 | 1.60E-06 |
| Species CHKCI006 sp900018345 | Breast Cancer | rs4980826 | 0.0529093 | 0.00969764 | 4.90E-08 |
| Species CHKCI006 sp900018345 | Breast Cancer | rs59673460 | -0.0509445 | 0.011147 | 4.90E-06 |
| Species CHKCI006 sp900018345 | Breast Cancer | rs61781392 | 0.0500522 | 0.0111458 | 7.10E-06 |
| Species CHKCI006 sp900018345 | Breast Cancer | rs71463082 | 0.110885 | 0.0233855 | 2.10E-06 |
| Species CHKCI006 sp900018345 | Breast Cancer | rs74785846 | 0.1972 | 0.0429594 | 4.40E-06 |
| Species CHKCI006 sp900018345 | Breast Cancer | rs78991715 | -0.167065 | 0.0365868 | 5.00E-06 |
| Species CHKCI006 sp900018345 | Breast Cancer | rs902730 | 0.0431238 | 0.00927927 | 3.40E-06 |
| Family Fibrobacteraceae | Breast Cancer | rs11040652 | 0.0131414 | 0.00296249 | 9.20E-06 |
| Family Fibrobacteraceae | Breast Cancer | rs11129117 | 0.0139802 | 0.00305237 | 4.60E-06 |
| Family Fibrobacteraceae | Breast Cancer | rs112448126 | 0.0456544 | 0.00904268 | 4.40E-07 |
| Family Fibrobacteraceae | Breast Cancer | rs12543777 | 0.0134018 | 0.00298661 | 7.20E-06 |
| Family Fibrobacteraceae | Breast Cancer | rs13391970 | -0.0245971 | 0.00525995 | 2.90E-06 |
| Family Fibrobacteraceae | Breast Cancer | rs144057235 | 0.056383 | 0.0124256 | 5.70E-06 |
| Family Fibrobacteraceae | Breast Cancer | rs17183864 | 0.0520869 | 0.0112418 | 3.60E-06 |
| Family Fibrobacteraceae | Breast Cancer | rs2418034 | -0.0178498 | 0.0035847 | 6.40E-07 |
| Family Fibrobacteraceae | Breast Cancer | rs34832380 | 0.0427599 | 0.00949516 | 6.70E-06 |
| Family Fibrobacteraceae | Breast Cancer | rs4783644 | -0.0165962 | 0.00366164 | 5.80E-06 |
| Family Fibrobacteraceae | Breast Cancer | rs56278671 | 0.0595988 | 0.0134351 | 9.20E-06 |
| Family Fibrobacteraceae | Breast Cancer | rs7138143 | -0.0158182 | 0.00308459 | 2.90E-07 |
| Family Fibrobacteraceae | Breast Cancer | rs7250654 | 0.0240002 | 0.00479289 | 5.50E-07 |
| Family Fibrobacteraceae | Breast Cancer | rs753897 | -0.0146198 | 0.00315518 | 3.60E-06 |
| Family Fibrobacteraceae | Breast Cancer | rs76620736 | 0.0183326 | 0.00411456 | 8.40E-06 |
| Family Fibrobacteraceae | Breast Cancer | rs9890225 | -0.0130659 | 0.00294692 | 9.30E-06 |
| Order Fibrobacterales | Breast Cancer | rs11129117 | 0.012965 | 0.00285959 | 5.80E-06 |
| Order Fibrobacterales | Breast Cancer | rs112448126 | 0.0414192 | 0.00847114 | 1.00E-06 |
| Order Fibrobacterales | Breast Cancer | rs13391970 | -0.0242061 | 0.00492763 | 9.00E-07 |
| Order Fibrobacterales | Breast Cancer | rs144057235 | 0.0534828 | 0.0116403 | 4.30E-06 |
| Order Fibrobacterales | Breast Cancer | rs17183864 | 0.0476534 | 0.0105311 | 6.00E-06 |
| Order Fibrobacterales | Breast Cancer | rs17587994 | 0.0123686 | 0.00277209 | 8.10E-06 |
| Order Fibrobacterales | Breast Cancer | rs2418034 | -0.0168189 | 0.00335825 | 5.50E-07 |
| Order Fibrobacterales | Breast Cancer | rs34832380 | 0.0411672 | 0.00889546 | 3.70E-06 |
| Order Fibrobacterales | Breast Cancer | rs4783644 | -0.0162073 | 0.0034302 | 2.30E-06 |
| Order Fibrobacterales | Breast Cancer | rs56278671 | 0.0563334 | 0.0125865 | 7.60E-06 |
| Order Fibrobacterales | Breast Cancer | rs62052260 | 0.0148742 | 0.00334976 | 9.00E-06 |
| Order Fibrobacterales | Breast Cancer | rs7138143 | -0.0147137 | 0.00288971 | 3.50E-07 |
| Order Fibrobacterales | Breast Cancer | rs7250654 | 0.0221246 | 0.0044903 | 8.30E-07 |
| Order Fibrobacterales | Breast Cancer | rs753897 | -0.0135664 | 0.00295584 | 4.40E-06 |
| Order Fibrobacterales | Breast Cancer | rs78102086 | -0.0281858 | 0.00616688 | 4.90E-06 |
| Class Fibrobacteria | Breast Cancer | rs10833653 | 0.0114752 | 0.00258854 | 9.30E-06 |
| Class Fibrobacteria | Breast Cancer | rs112448126 | 0.0372008 | 0.00780632 | 1.90E-06 |
| Class Fibrobacteria | Breast Cancer | rs13391970 | -0.0227082 | 0.00454096 | 5.70E-07 |
| Class Fibrobacteria | Breast Cancer | rs144057235 | 0.0491863 | 0.0107268 | 4.50E-06 |
| Class Fibrobacteria | Breast Cancer | rs17183864 | 0.0438587 | 0.00970457 | 6.20E-06 |
| Class Fibrobacteria | Breast Cancer | rs1860287 | -0.0121509 | 0.00270473 | 7.00E-06 |
| Class Fibrobacteria | Breast Cancer | rs2418034 | -0.0154083 | 0.00309471 | 6.40E-07 |
| Class Fibrobacteria | Breast Cancer | rs28730413 | -0.0120771 | 0.0025457 | 2.10E-06 |
| Class Fibrobacteria | Breast Cancer | rs34657897 | 0.0333696 | 0.00719424 | 3.50E-06 |
| Class Fibrobacteria | Breast Cancer | rs34814525 | 0.0116821 | 0.00255655 | 4.90E-06 |
| Class Fibrobacteria | Breast Cancer | rs34905826 | 0.0307628 | 0.00651171 | 2.30E-06 |
| Class Fibrobacteria | Breast Cancer | rs4783644 | -0.0145893 | 0.00316102 | 3.90E-06 |
| Class Fibrobacteria | Breast Cancer | rs56278671 | 0.0530163 | 0.0115988 | 4.90E-06 |
| Class Fibrobacteria | Breast Cancer | rs61782717 | 0.0494751 | 0.0111518 | 9.10E-06 |
| Class Fibrobacteria | Breast Cancer | rs62052260 | 0.0145689 | 0.00308691 | 2.40E-06 |
| Class Fibrobacteria | Breast Cancer | rs6599051 | 0.0137088 | 0.00303597 | 6.30E-06 |
| Class Fibrobacteria | Breast Cancer | rs7138143 | -0.0140111 | 0.00266294 | 1.40E-07 |
| Class Fibrobacteria | Breast Cancer | rs7250654 | 0.0206978 | 0.00413798 | 5.70E-07 |
| Class Fibrobacteria | Breast Cancer | rs753897 | -0.0126427 | 0.0027239 | 3.50E-06 |
| Class Fibrobacteria | Breast Cancer | rs76161196 | 0.0259466 | 0.0058024 | 7.80E-06 |
| Species Megamonas funiformis | Breast Cancer | rs10025256 | 0.0662322 | 0.0147327 | 6.90E-06 |
| Species Megamonas funiformis | Breast Cancer | rs10196495 | 0.0490404 | 0.0107514 | 5.10E-06 |
| Species Megamonas funiformis | Breast Cancer | rs112910828 | 0.149515 | 0.031759 | 2.50E-06 |
| Species Megamonas funiformis | Breast Cancer | rs114894604 | 0.14832 | 0.0312218 | 2.00E-06 |
| Species Megamonas funiformis | Breast Cancer | rs116338908 | 0.236574 | 0.0520765 | 5.60E-06 |
| Species Megamonas funiformis | Breast Cancer | rs116895877 | 0.20284 | 0.0444071 | 4.90E-06 |
| Species Megamonas funiformis | Breast Cancer | rs117243639 | 0.227846 | 0.0436466 | 1.80E-07 |
| Species Megamonas funiformis | Breast Cancer | rs117992479 | 0.243606 | 0.0490238 | 6.70E-07 |
| Species Megamonas funiformis | Breast Cancer | rs12491882 | 0.0491718 | 0.010936 | 6.90E-06 |
| Species Megamonas funiformis | Breast Cancer | rs138309059 | 0.1929 | 0.0424601 | 5.50E-06 |
| Species Megamonas funiformis | Breast Cancer | rs139556348 | 0.168807 | 0.0367688 | 4.40E-06 |
| Species Megamonas funiformis | Breast Cancer | rs139616585 | 0.22327 | 0.042319 | 1.30E-07 |
| Species Megamonas funiformis | Breast Cancer | rs149221610 | 0.193402 | 0.0428127 | 6.30E-06 |
| Species Megamonas funiformis | Breast Cancer | rs17456563 | 0.129309 | 0.0279236 | 3.60E-06 |
| Species Megamonas funiformis | Breast Cancer | rs1821595 | 0.192357 | 0.0427188 | 6.70E-06 |
| Species Megamonas funiformis | Breast Cancer | rs190157640 | 0.213602 | 0.0478313 | 8.00E-06 |
| Species Megamonas funiformis | Breast Cancer | rs1999144 | 0.112175 | 0.0228878 | 9.50E-07 |
| Species Megamonas funiformis | Breast Cancer | rs2281616 | 0.232607 | 0.0471002 | 7.90E-07 |
| Species Megamonas funiformis | Breast Cancer | rs2451340 | 0.14932 | 0.0311923 | 1.70E-06 |
| Species Megamonas funiformis | Breast Cancer | rs35481789 | 0.0543384 | 0.0121015 | 7.10E-06 |
| Species Megamonas funiformis | Breast Cancer | rs36112940 | 0.124939 | 0.0251967 | 7.10E-07 |
| Species Megamonas funiformis | Breast Cancer | rs3783925 | -0.0510338 | 0.0111127 | 4.40E-06 |
| Species Megamonas funiformis | Breast Cancer | rs4658309 | -0.0505118 | 0.0114103 | 9.60E-06 |
| Species Megamonas funiformis | Breast Cancer | rs56009508 | 0.220364 | 0.0461912 | 1.80E-06 |
| Species Megamonas funiformis | Breast Cancer | rs56293483 | 0.0670958 | 0.0150349 | 8.10E-06 |
| Species Megamonas funiformis | Breast Cancer | rs58277973 | 0.121583 | 0.0270259 | 6.80E-06 |
| Species Megamonas funiformis | Breast Cancer | rs6976718 | 0.0487653 | 0.0106901 | 5.10E-06 |
| Species Megamonas funiformis | Breast Cancer | rs71539626 | 0.223808 | 0.0494546 | 6.00E-06 |
| Species Megamonas funiformis | Breast Cancer | rs73789361 | 0.146333 | 0.0321678 | 5.40E-06 |
| Species Megamonas funiformis | Breast Cancer | rs75550369 | 0.22381 | 0.0482229 | 3.50E-06 |
| Species Megamonas funiformis | Breast Cancer | rs75824665 | 0.168225 | 0.0370359 | 5.60E-06 |
| Species Megamonas funiformis | Breast Cancer | rs76400051 | 0.0816296 | 0.0179743 | 5.60E-06 |
| Species Megamonas funiformis | Breast Cancer | rs77458346 | 0.180514 | 0.0398821 | 6.00E-06 |
| Species Megamonas funiformis | Breast Cancer | rs78393153 | 0.23191 | 0.0418813 | 3.10E-08 |
| Species Megamonas funiformis | Breast Cancer | rs79498746 | 0.121598 | 0.0257342 | 2.30E-06 |
| Species Megamonas funiformis | Breast Cancer | rs79605300 | 0.176044 | 0.0397788 | 9.60E-06 |
| Species Megamonas funiformis | Breast Cancer | rs79747352 | 0.181793 | 0.0366964 | 7.30E-07 |
| Species Megamonas funiformis | Breast Cancer | rs874689 | 0.0534554 | 0.0116456 | 4.40E-06 |
| Species Megamonas funiformis | Breast Cancer | rs908047 | 0.0572192 | 0.0123284 | 3.50E-06 |
| Species Prevotella bivia | Breast Cancer | rs117221088 | 0.1845 | 0.0410279 | 6.90E-06 |
| Species Prevotella bivia | Breast Cancer | rs12070072 | 0.0568365 | 0.0119981 | 2.20E-06 |
| Species Prevotella bivia | Breast Cancer | rs12200654 | 0.115444 | 0.0249668 | 3.80E-06 |
| Species Prevotella bivia | Breast Cancer | rs13037592 | 0.222829 | 0.0501418 | 8.80E-06 |
| Species Prevotella bivia | Breast Cancer | rs149009235 | 0.224698 | 0.0446353 | 4.80E-07 |
| Species Prevotella bivia | Breast Cancer | rs2166098 | 0.141595 | 0.0313895 | 6.50E-06 |
| Species Prevotella bivia | Breast Cancer | rs4610049 | 0.0657885 | 0.0138263 | 2.00E-06 |
| Species Prevotella bivia | Breast Cancer | rs471538 | -0.0692225 | 0.0124687 | 2.80E-08 |
| Species Prevotella bivia | Breast Cancer | rs6565531 | 0.0526467 | 0.0119033 | 9.70E-06 |
| Species Prevotella bivia | Breast Cancer | rs675632 | 0.158716 | 0.0334912 | 2.10E-06 |
| Species Prevotella bivia | Breast Cancer | rs77898767 | 0.187434 | 0.0415782 | 6.50E-06 |
| Species Prevotella bivia | Breast Cancer | rs78007943 | -0.122704 | 0.0276713 | 9.20E-06 |
| Species Prevotella bivia | Breast Cancer | rs7821969 | -0.0664149 | 0.0134515 | 7.90E-07 |
| Species Prevotella bivia | Breast Cancer | rs78254123 | 0.226874 | 0.0506372 | 7.50E-06 |
| Species Prevotellamassilia | Breast Cancer | rs117293965 | -0.206411 | 0.042606 | 1.30E-06 |
| Species Prevotellamassilia | Breast Cancer | rs117476696 | 0.277274 | 0.0551208 | 4.90E-07 |
| Species Prevotellamassilia | Breast Cancer | rs117555310 | 0.194175 | 0.0407989 | 1.90E-06 |
| Species Prevotellamassilia | Breast Cancer | rs11812171 | 0.262774 | 0.0537724 | 1.00E-06 |
| Species Prevotellamassilia | Breast Cancer | rs139488692 | 0.336464 | 0.074174 | 5.70E-06 |
| Species Prevotellamassilia | Breast Cancer | rs142992142 | 0.341165 | 0.0706808 | 1.40E-06 |
| Species Prevotellamassilia | Breast Cancer | rs144042766 | 0.275672 | 0.058329 | 2.30E-06 |
| Species Prevotellamassilia | Breast Cancer | rs202091594 | 0.170298 | 0.0341205 | 6.00E-07 |
| Species Prevotellamassilia | Breast Cancer | rs2140551 | -0.10983 | 0.0181879 | 1.60E-09 |
| Species Prevotellamassilia | Breast Cancer | rs324324 | 0.101936 | 0.0219302 | 3.30E-06 |
| Species Prevotellamassilia | Breast Cancer | rs62148957 | 0.257941 | 0.0559507 | 4.00E-06 |
| Species Prevotellamassilia | Breast Cancer | rs6699072 | 0.17703 | 0.0386407 | 4.60E-06 |
| Species Prevotellamassilia | Breast Cancer | rs6825590 | 0.0801551 | 0.0179531 | 8.00E-06 |
| Species Prevotellamassilia | Breast Cancer | rs9970188 | -0.108387 | 0.0232379 | 3.10E-06 |
| Genus RUG147 | Breast Cancer | rs112435825 | 0.0702669 | 0.0150659 | 3.10E-06 |
| Genus RUG147 | Breast Cancer | rs117684381 | 0.0598415 | 0.0127173 | 2.50E-06 |
| Genus RUG147 | Breast Cancer | rs4802988 | -0.0321018 | 0.00646424 | 6.80E-07 |
| Genus RUG147 | Breast Cancer | rs6062357 | 0.0274681 | 0.00614222 | 7.70E-06 |
| Genus RUG147 | Breast Cancer | rs74523015 | -0.0709708 | 0.0156911 | 6.10E-06 |
| Genus RUG147 | Breast Cancer | rs79557454 | -0.0588962 | 0.0129057 | 5.00E-06 |
| Species UBA7177 sp002491225 | Breast Cancer | rs10763683 | -0.026154 | 0.00557496 | 2.70E-06 |
| Species UBA7177 sp002491225 | Breast Cancer | rs111754047 | -0.0832652 | 0.0158451 | 1.50E-07 |
| Species UBA7177 sp002491225 | Breast Cancer | rs111760505 | -0.0516161 | 0.010954 | 2.50E-06 |
| Species UBA7177 sp002491225 | Breast Cancer | rs115508224 | 0.0581738 | 0.0129445 | 7.00E-06 |
| Species UBA7177 sp002491225 | Breast Cancer | rs115906416 | -0.0968046 | 0.0200442 | 1.40E-06 |
| Species UBA7177 sp002491225 | Breast Cancer | rs11706534 | 0.0537092 | 0.00884725 | 1.30E-09 |
| Species UBA7177 sp002491225 | Breast Cancer | rs12561867 | -0.061767 | 0.012574 | 9.00E-07 |
| Species UBA7177 sp002491225 | Breast Cancer | rs150921994 | 0.0873509 | 0.0185454 | 2.50E-06 |
| Species UBA7177 sp002491225 | Breast Cancer | rs155955 | 0.0867861 | 0.0184343 | 2.50E-06 |
| Species UBA7177 sp002491225 | Breast Cancer | rs2731799 | -0.0935361 | 0.0199731 | 2.80E-06 |
| Species UBA7177 sp002491225 | Breast Cancer | rs2948048 | 0.0310449 | 0.00669064 | 3.50E-06 |
| Species UBA7177 sp002491225 | Breast Cancer | rs3805739 | -0.0443047 | 0.00996421 | 8.70E-06 |
| Species UBA7177 sp002491225 | Breast Cancer | rs62289166 | -0.0438792 | 0.00816634 | 7.70E-08 |
| Species UBA7177 sp002491225 | Breast Cancer | rs6708696 | -0.0292654 | 0.00618544 | 2.20E-06 |
| Species UBA7177 sp002491225 | Breast Cancer | rs7216601 | -0.0309104 | 0.00650898 | 2.00E-06 |
| Species UBA7177 sp002491225 | Breast Cancer | rs73303077 | -0.03739 | 0.00845833 | 9.80E-06 |
| Species UBA7177 sp002491225 | Breast Cancer | rs75629510 | -0.0438567 | 0.00972726 | 6.50E-06 |
| Species UBA7177 sp002491225 | Breast Cancer | rs76566606 | -0.0446829 | 0.00983789 | 5.60E-06 |
| Species UBA7177 sp002491225 | Breast Cancer | rs78003791 | 0.0561315 | 0.0117944 | 1.90E-06 |
| Species UBA7177 sp002491225 | Breast Cancer | rs7903786 | 0.0259431 | 0.00573016 | 6.00E-06 |
| Genus Veillonella | Breast Cancer | rs112835958 | 0.210843 | 0.042223 | 5.90E-07 |
| Genus Veillonella | Breast Cancer | rs118081085 | 0.117521 | 0.0265165 | 9.30E-06 |
| Genus Veillonella | Breast Cancer | rs2555391 | 0.114619 | 0.0241503 | 2.10E-06 |
| Genus Veillonella | Breast Cancer | rs6666482 | -0.0816776 | 0.0181235 | 6.60E-06 |
| Genus Veillonella | Breast Cancer | rs73344440 | 0.174955 | 0.0387665 | 6.40E-06 |
| Genus Veillonella | Breast Cancer | rs73855513 | 0.225391 | 0.0463064 | 1.10E-06 |
| Genus Veillonella | Breast Cancer | rs78042276 | 0.216774 | 0.0454969 | 1.90E-06 |
| Genus Veillonella | Breast Cancer | rs78552828 | 0.180177 | 0.0394529 | 4.90E-06 |
| Species Phascolarctobacterium sp003150755 | Breast Cancer | rs10833244 | 0.154294 | 0.0335862 | 4.30E-06 |
| Species Phascolarctobacterium sp003150755 | Breast Cancer | rs10862672 | 0.246958 | 0.0554249 | 8.40E-06 |
| Species Phascolarctobacterium sp003150755 | Breast Cancer | rs10999818 | -0.124625 | 0.0271786 | 4.50E-06 |
| Species Phascolarctobacterium sp003150755 | Breast Cancer | rs11047795 | -0.066279 | 0.0147873 | 7.40E-06 |
| Species Phascolarctobacterium sp003150755 | Breast Cancer | rs116447189 | 0.162444 | 0.0365516 | 8.80E-06 |
| Species Phascolarctobacterium sp003150755 | Breast Cancer | rs118117698 | 0.224317 | 0.0507465 | 9.90E-06 |
| Species Phascolarctobacterium sp003150755 | Breast Cancer | rs12891383 | -0.0706764 | 0.0142399 | 6.90E-07 |
| Species Phascolarctobacterium sp003150755 | Breast Cancer | rs182171742 | 0.194516 | 0.0431744 | 6.60E-06 |
| Species Phascolarctobacterium sp003150755 | Breast Cancer | rs2221099 | -0.0581672 | 0.0127468 | 5.00E-06 |
| Species Phascolarctobacterium sp003150755 | Breast Cancer | rs3094786 | 0.131686 | 0.0294522 | 7.80E-06 |
| Species Phascolarctobacterium sp003150755 | Breast Cancer | rs4676213 | -0.114348 | 0.0251706 | 5.50E-06 |
| Species Phascolarctobacterium sp003150755 | Breast Cancer | rs4681028 | 0.059889 | 0.0132248 | 5.90E-06 |
| Species Phascolarctobacterium sp003150755 | Breast Cancer | rs6023456 | 0.16576 | 0.0367317 | 6.40E-06 |
| Species Phascolarctobacterium sp003150755 | Breast Cancer | rs62262357 | 0.185413 | 0.0311101 | 2.50E-09 |
| Species Phascolarctobacterium sp003150755 | Breast Cancer | rs62296310 | -0.0668427 | 0.013971 | 1.70E-06 |
| Species Phascolarctobacterium sp003150755 | Breast Cancer | rs62324492 | 0.218253 | 0.0448669 | 1.10E-06 |
| Species Phascolarctobacterium sp003150755 | Breast Cancer | rs769046 | 0.06551 | 0.0144073 | 5.40E-06 |
| Species Phascolarctobacterium sp003150755 | Breast Cancer | rs78197954 | 0.101575 | 0.0222936 | 5.20E-06 |
|  |  |  |  |  |  |
|  |  |  |  |  |  |
| **SNP for MR analysis between Mediator and Breast cancer** | | | | | |
| Exposure | Outcome | SNP | beta | Se | Pvalue |
| CD24+ CD27+ AC | Breast Cancer | rs10759006 | 0.1133 | 0.02556 | 9.51E-06 |
| CD24+ CD27+ AC | Breast Cancer | rs10762541 | 0.1488 | 0.02978 | 6.09E-07 |
| CD24+ CD27+ AC | Breast Cancer | rs10942726 | 0.1188 | 0.02672 | 8.93E-06 |
| CD24+ CD27+ AC | Breast Cancer | rs11649808 | 0.1546 | 0.03472 | 8.75E-06 |
| CD24+ CD27+ AC | Breast Cancer | rs11653761 | -0.118 | 0.0247 | 1.84E-06 |
| CD24+ CD27+ AC | Breast Cancer | rs118092259 | -0.2901 | 0.06345 | 4.98E-06 |
| CD24+ CD27+ AC | Breast Cancer | rs12874404 | 0.1691 | 0.02653 | 2.04E-10 |
| CD24+ CD27+ AC | Breast Cancer | rs138329462 | -0.4374 | 0.09365 | 3.11E-06 |
| CD24+ CD27+ AC | Breast Cancer | rs144295598 | 0.5664 | 0.1203 | 2.60E-06 |
| CD24+ CD27+ AC | Breast Cancer | rs149085540 | -0.4318 | 0.09574 | 6.67E-06 |
| CD24+ CD27+ AC | Breast Cancer | rs16867231 | 0.5129 | 0.1154 | 8.98E-06 |
| CD24+ CD27+ AC | Breast Cancer | rs35057381 | 0.1344 | 0.02969 | 6.18E-06 |
| CD24+ CD27+ AC | Breast Cancer | rs4076316 | -0.1404 | 0.02729 | 2.84E-07 |
| CD24+ CD27+ AC | Breast Cancer | rs56033406 | 0.312 | 0.06692 | 3.23E-06 |
| CD24+ CD27+ AC | Breast Cancer | rs7254039 | -0.2606 | 0.0585 | 8.64E-06 |
| CD24+ CD27+ AC | Breast Cancer | rs728776 | -0.1712 | 0.03808 | 7.09E-06 |
| CD24+ CD27+ AC | Breast Cancer | rs73192651 | 0.4411 | 0.08893 | 7.38E-07 |
| CD24+ CD27+ AC | Breast Cancer | rs75766272 | -0.4055 | 0.08609 | 2.57E-06 |
| CD24+ CD27+ AC | Breast Cancer | rs7640784 | -0.1111 | 0.02476 | 7.48E-06 |
| CD24+ CD27+ AC | Breast Cancer | rs76552872 | 0.283 | 0.06219 | 5.54E-06 |
| CD24+ CD27+ AC | Breast Cancer | rs77762131 | 0.2815 | 0.05774 | 1.13E-06 |
| CD24+ CD27+ AC | Breast Cancer | rs79207264 | 0.1752 | 0.0393 | 8.47E-06 |
| IgD- CD38br AC | Breast Cancer | rs10815908 | 0.2526 | 0.0538 | 2.76E-06 |
| IgD- CD38br AC | Breast Cancer | rs11605946 | 0.1457 | 0.03191 | 5.16E-06 |
| IgD- CD38br AC | Breast Cancer | rs11609248 | -0.1355 | 0.03014 | 7.11E-06 |
| IgD- CD38br AC | Breast Cancer | rs141490841 | 0.3828 | 0.08506 | 6.99E-06 |
| IgD- CD38br AC | Breast Cancer | rs17037450 | -0.3311 | 0.07236 | 4.91E-06 |
| IgD- CD38br AC | Breast Cancer | rs2075365 | -0.1142 | 0.02477 | 4.15E-06 |
| IgD- CD38br AC | Breast Cancer | rs2792563 | -0.108 | 0.02437 | 9.60E-06 |
| IgD- CD38br AC | Breast Cancer | rs478251 | 0.1689 | 0.03431 | 8.86E-07 |
| IgD- CD38br AC | Breast Cancer | rs62353585 | 0.3438 | 0.07709 | 8.48E-06 |
| IgD- CD38br AC | Breast Cancer | rs66491584 | -0.1466 | 0.02957 | 7.43E-07 |
| IgD- CD38br AC | Breast Cancer | rs72834736 | 0.3465 | 0.07762 | 8.31E-06 |
| IgD- CD38br AC | Breast Cancer | rs74011406 | 0.1928 | 0.04113 | 2.87E-06 |
| IgD- CD38br AC | Breast Cancer | rs75544731 | 0.4192 | 0.09428 | 8.99E-06 |
| IgD- CD38br AC | Breast Cancer | rs78678130 | -0.1707 | 0.03819 | 8.03E-06 |
| IgD- CD38br AC | Breast Cancer | rs79801395 | 0.1973 | 0.04374 | 6.69E-06 |
| CD62L- myeloid DC AC | Breast Cancer | rs10779896 | -0.2364 | 0.05339 | 9.83E-06 |
| CD62L- myeloid DC AC | Breast Cancer | rs112501424 | 0.1694 | 0.03788 | 8.04E-06 |
| CD62L- myeloid DC AC | Breast Cancer | rs115805162 | -0.2833 | 0.03326 | 2.43E-17 |
| CD62L- myeloid DC AC | Breast Cancer | rs116910026 | 0.2902 | 0.05778 | 5.39E-07 |
| CD62L- myeloid DC AC | Breast Cancer | rs11830537 | -0.314 | 0.06982 | 7.12E-06 |
| CD62L- myeloid DC AC | Breast Cancer | rs13148506 | 0.1304 | 0.02714 | 1.62E-06 |
| CD62L- myeloid DC AC | Breast Cancer | rs148765873 | -0.3791 | 0.06661 | 1.36E-08 |
| CD62L- myeloid DC AC | Breast Cancer | rs17438107 | 0.2161 | 0.04523 | 1.84E-06 |
| CD62L- myeloid DC AC | Breast Cancer | rs35519924 | 0.1281 | 0.02803 | 5.04E-06 |
| CD62L- myeloid DC AC | Breast Cancer | rs4997747 | 0.1346 | 0.02844 | 2.29E-06 |
| CD62L- myeloid DC AC | Breast Cancer | rs5767014 | -0.1397 | 0.03083 | 6.08E-06 |
| CD62L- myeloid DC AC | Breast Cancer | rs71632979 | 0.8094 | 0.02787 | 9.84E-166 |
| CD62L- myeloid DC AC | Breast Cancer | rs71639910 | 0.401 | 0.05202 | 1.66E-14 |
| CD62L- myeloid DC AC | Breast Cancer | rs73172229 | -0.1908 | 0.03986 | 1.77E-06 |
| CD62L- myeloid DC AC | Breast Cancer | rs75072970 | 0.4285 | 0.05879 | 3.88E-13 |
| Activated & secreting Treg AC | Breast Cancer | rs10905876 | -0.1232 | 0.02732 | 6.75E-06 |
| Activated & secreting Treg AC | Breast Cancer | rs111992639 | -0.7163 | 0.1247 | 9.94E-09 |
| Activated & secreting Treg AC | Breast Cancer | rs145600407 | -0.5371 | 0.1044 | 2.85E-07 |
| Activated & secreting Treg AC | Breast Cancer | rs149951966 | 0.2078 | 0.04392 | 2.33E-06 |
| Activated & secreting Treg AC | Breast Cancer | rs17717645 | 0.3923 | 0.08188 | 1.73E-06 |
| Activated & secreting Treg AC | Breast Cancer | rs61839660 | 0.3316 | 0.05733 | 7.93E-09 |
| Activated & secreting Treg AC | Breast Cancer | rs7162344 | -0.1207 | 0.02577 | 2.91E-06 |
| Activated & secreting Treg AC | Breast Cancer | rs72833913 | 0.1855 | 0.04169 | 8.83E-06 |
| Activated & secreting Treg AC | Breast Cancer | rs75596315 | 0.2653 | 0.05762 | 4.28E-06 |
| Activated & secreting Treg AC | Breast Cancer | rs76202906 | -0.3574 | 0.07469 | 1.78E-06 |
| Activated & secreting Treg AC | Breast Cancer | rs9709738 | -0.1302 | 0.02894 | 6.99E-06 |
| EM DN (CD4-CD8-) %T cell | Breast Cancer | rs10225877 | -0.1278 | 0.02656 | 1.55E-06 |
| EM DN (CD4-CD8-) %T cell | Breast Cancer | rs11180778 | 0.1213 | 0.02717 | 8.31E-06 |
| EM DN (CD4-CD8-) %T cell | Breast Cancer | rs111902980 | 0.4328 | 0.08768 | 8.35E-07 |
| EM DN (CD4-CD8-) %T cell | Breast Cancer | rs113075410 | 0.2447 | 0.05465 | 7.79E-06 |
| EM DN (CD4-CD8-) %T cell | Breast Cancer | rs113500908 | 0.3476 | 0.05835 | 2.83E-09 |
| EM DN (CD4-CD8-) %T cell | Breast Cancer | rs116230798 | -0.2684 | 0.05681 | 2.40E-06 |
| EM DN (CD4-CD8-) %T cell | Breast Cancer | rs11635763 | 0.1159 | 0.02481 | 3.09E-06 |
| EM DN (CD4-CD8-) %T cell | Breast Cancer | rs116877116 | 0.1973 | 0.04041 | 1.09E-06 |
| EM DN (CD4-CD8-) %T cell | Breast Cancer | rs13210358 | 0.4707 | 0.07114 | 4.24E-11 |
| EM DN (CD4-CD8-) %T cell | Breast Cancer | rs140247402 | -0.2519 | 0.05542 | 5.68E-06 |
| EM DN (CD4-CD8-) %T cell | Breast Cancer | rs141519401 | 0.1737 | 0.03911 | 9.22E-06 |
| EM DN (CD4-CD8-) %T cell | Breast Cancer | rs28555522 | 0.1258 | 0.02629 | 1.78E-06 |
| EM DN (CD4-CD8-) %T cell | Breast Cancer | rs28647367 | 0.2288 | 0.05128 | 8.41E-06 |
| EM DN (CD4-CD8-) %T cell | Breast Cancer | rs34546581 | 0.2019 | 0.03063 | 5.06E-11 |
| EM DN (CD4-CD8-) %T cell | Breast Cancer | rs437936 | -0.1625 | 0.03568 | 5.43E-06 |
| EM DN (CD4-CD8-) %T cell | Breast Cancer | rs4583837 | 0.2578 | 0.05553 | 3.58E-06 |
| EM DN (CD4-CD8-) %T cell | Breast Cancer | rs55781756 | 0.1501 | 0.0323 | 3.52E-06 |
| EM DN (CD4-CD8-) %T cell | Breast Cancer | rs75679242 | -0.1752 | 0.03783 | 3.79E-06 |
| EM DN (CD4-CD8-) %T cell | Breast Cancer | rs78118522 | -0.1278 | 0.02756 | 3.66E-06 |
| EM DN (CD4-CD8-) %T cell | Breast Cancer | rs78522661 | 0.1964 | 0.04026 | 1.11E-06 |
| EM DN (CD4-CD8-) %T cell | Breast Cancer | rs9896155 | 0.1315 | 0.02587 | 3.91E-07 |
| CD4+ AC | Breast Cancer | rs10040274 | 0.132 | 0.02702 | 1.06E-06 |
| CD4+ AC | Breast Cancer | rs10051472 | 0.1181 | 0.02592 | 5.39E-06 |
| CD4+ AC | Breast Cancer | rs117563350 | -0.324 | 0.06938 | 3.12E-06 |
| CD4+ AC | Breast Cancer | rs1849876 | 0.1324 | 0.02769 | 1.81E-06 |
| CD4+ AC | Breast Cancer | rs28578320 | 0.1133 | 0.02558 | 9.78E-06 |
| CD4+ AC | Breast Cancer | rs2972073 | 0.1612 | 0.03603 | 7.86E-06 |
| CD4+ AC | Breast Cancer | rs3129891 | -0.1631 | 0.03448 | 2.32E-06 |
| CD4+ AC | Breast Cancer | rs3184504 | -0.1838 | 0.02676 | 7.66E-12 |
| CD4+ AC | Breast Cancer | rs414743 | 0.1352 | 0.02918 | 3.73E-06 |
| CD4+ AC | Breast Cancer | rs4425753 | 0.1776 | 0.0374 | 2.14E-06 |
| CD4+ AC | Breast Cancer | rs61935579 | -0.2776 | 0.06065 | 4.88E-06 |
| CD4+ AC | Breast Cancer | rs7162344 | -0.1136 | 0.02442 | 3.37E-06 |
| CD4+ AC | Breast Cancer | rs72960548 | 0.2456 | 0.04852 | 4.34E-07 |
| CD4+ AC | Breast Cancer | rs73132830 | 0.1352 | 0.03023 | 7.94E-06 |
| CD4+ AC | Breast Cancer | rs7537437 | -0.1404 | 0.03159 | 9.07E-06 |
| CD4+ AC | Breast Cancer | rs75494288 | -0.3345 | 0.07401 | 6.40E-06 |
| CD4+ AC | Breast Cancer | rs75697875 | 0.2783 | 0.06013 | 3.81E-06 |
| CD4+ AC | Breast Cancer | rs78628680 | 0.3565 | 0.08034 | 9.38E-06 |
| CD4+ AC | Breast Cancer | rs905461 | 0.1209 | 0.02726 | 9.49E-06 |
| CD4+ AC | Breast Cancer | rs9271772 | 0.1877 | 0.03517 | 1.01E-07 |
| HLA DR+ CD4+ AC | Breast Cancer | rs10787670 | -0.1963 | 0.04335 | 6.14E-06 |
| HLA DR+ CD4+ AC | Breast Cancer | rs11251832 | 0.1894 | 0.04192 | 6.44E-06 |
| HLA DR+ CD4+ AC | Breast Cancer | rs11502961 | 0.1625 | 0.03468 | 2.88E-06 |
| HLA DR+ CD4+ AC | Breast Cancer | rs12166155 | 0.1146 | 0.025 | 4.70E-06 |
| HLA DR+ CD4+ AC | Breast Cancer | rs1322067 | -0.1511 | 0.02575 | 4.75E-09 |
| HLA DR+ CD4+ AC | Breast Cancer | rs17198888 | -0.2281 | 0.04783 | 1.93E-06 |
| HLA DR+ CD4+ AC | Breast Cancer | rs2064600 | -0.3548 | 0.07367 | 1.53E-06 |
| HLA DR+ CD4+ AC | Breast Cancer | rs2977197 | -0.1662 | 0.03693 | 7.03E-06 |
| HLA DR+ CD4+ AC | Breast Cancer | rs3087456 | -0.2926 | 0.02777 | 1.40E-25 |
| HLA DR+ CD4+ AC | Breast Cancer | rs3134968 | 0.3737 | 0.07884 | 2.23E-06 |
| HLA DR+ CD4+ AC | Breast Cancer | rs55849757 | -0.1129 | 0.02435 | 3.69E-06 |
| HLA DR+ CD4+ AC | Breast Cancer | rs62166973 | -0.4445 | 0.09861 | 6.76E-06 |
| HLA DR+ CD4+ AC | Breast Cancer | rs62417423 | -0.1396 | 0.03071 | 5.67E-06 |
| HLA DR+ CD4+ AC | Breast Cancer | rs6767083 | 0.1335 | 0.02844 | 2.78E-06 |
| HLA DR+ CD4+ AC | Breast Cancer | rs6891593 | 0.3491 | 0.07676 | 5.62E-06 |
| HLA DR+ CD4+ AC | Breast Cancer | rs7150202 | 0.1473 | 0.03164 | 3.33E-06 |
| HLA DR+ CD4+ AC | Breast Cancer | rs7405251 | -0.1151 | 0.02473 | 3.35E-06 |
| HLA DR+ CD4+ AC | Breast Cancer | rs78229126 | 0.1603 | 0.03437 | 3.23E-06 |
| HLA DR+ CD4+ AC | Breast Cancer | rs79769908 | -0.1766 | 0.03991 | 9.98E-06 |
| HLA DR+ CD4+ AC | Breast Cancer | rs9613063 | -0.1553 | 0.03005 | 2.50E-07 |
| B cell %lymphocyte | Breast Cancer | rs112526427 | 0.3025 | 0.06807 | 9.10E-06 |
| B cell %lymphocyte | Breast Cancer | rs115698234 | 0.5437 | 0.1169 | 3.43E-06 |
| B cell %lymphocyte | Breast Cancer | rs11627066 | -0.1185 | 0.02483 | 1.90E-06 |
| B cell %lymphocyte | Breast Cancer | rs12874404 | 0.2272 | 0.02633 | 8.99E-18 |
| B cell %lymphocyte | Breast Cancer | rs13108273 | 0.1849 | 0.04159 | 9.03E-06 |
| B cell %lymphocyte | Breast Cancer | rs1408899 | 0.1297 | 0.02886 | 7.21E-06 |
| B cell %lymphocyte | Breast Cancer | rs144426024 | 0.5227 | 0.114 | 4.71E-06 |
| B cell %lymphocyte | Breast Cancer | rs147049458 | 0.5051 | 0.1073 | 2.61E-06 |
| B cell %lymphocyte | Breast Cancer | rs150774187 | 0.1705 | 0.0357 | 1.86E-06 |
| B cell %lymphocyte | Breast Cancer | rs1557987 | 0.1431 | 0.03213 | 8.64E-06 |
| B cell %lymphocyte | Breast Cancer | rs1572133 | -0.1398 | 0.03093 | 6.41E-06 |
| B cell %lymphocyte | Breast Cancer | rs17113367 | 0.2019 | 0.04192 | 1.51E-06 |
| B cell %lymphocyte | Breast Cancer | rs191368650 | -0.4331 | 0.09277 | 3.14E-06 |
| B cell %lymphocyte | Breast Cancer | rs2223650 | -0.1173 | 0.0253 | 3.70E-06 |
| B cell %lymphocyte | Breast Cancer | rs2395904 | -0.1413 | 0.02766 | 3.44E-07 |
| B cell %lymphocyte | Breast Cancer | rs2679580 | -0.1171 | 0.02431 | 1.51E-06 |
| B cell %lymphocyte | Breast Cancer | rs34588768 | -0.42 | 0.0944 | 8.90E-06 |
| B cell %lymphocyte | Breast Cancer | rs35911859 | 0.3174 | 0.06906 | 4.47E-06 |
| B cell %lymphocyte | Breast Cancer | rs3738088 | 0.5216 | 0.1115 | 2.97E-06 |
| B cell %lymphocyte | Breast Cancer | rs4748742 | 0.1147 | 0.02437 | 2.61E-06 |
| B cell %lymphocyte | Breast Cancer | rs55658072 | 0.1135 | 0.02541 | 8.23E-06 |
| B cell %lymphocyte | Breast Cancer | rs5754162 | 0.1356 | 0.02545 | 1.04E-07 |
| B cell %lymphocyte | Breast Cancer | rs58114937 | 0.1732 | 0.03637 | 2.00E-06 |
| B cell %lymphocyte | Breast Cancer | rs66777340 | -0.1493 | 0.02419 | 7.63E-10 |
| B cell %lymphocyte | Breast Cancer | rs6985401 | -0.4402 | 0.09906 | 9.12E-06 |
| B cell %lymphocyte | Breast Cancer | rs72941136 | 0.2075 | 0.04021 | 2.59E-07 |
| B cell %lymphocyte | Breast Cancer | rs73520190 | 0.4121 | 0.08319 | 7.62E-07 |
| B cell %lymphocyte | Breast Cancer | rs74535587 | -0.4522 | 0.1022 | 9.95E-06 |
| B cell %lymphocyte | Breast Cancer | rs7483614 | 0.1453 | 0.03221 | 6.68E-06 |
| B cell %lymphocyte | Breast Cancer | rs77233730 | -0.2127 | 0.04478 | 2.10E-06 |
| B cell %lymphocyte | Breast Cancer | rs7990182 | -0.1288 | 0.02457 | 1.69E-07 |
| B cell %lymphocyte | Breast Cancer | rs9368867 | -0.1301 | 0.02649 | 9.47E-07 |
| B cell %lymphocyte | Breast Cancer | rs9520905 | 0.1266 | 0.02497 | 4.19E-07 |
| CD28+ CD45RA- CD8dim AC | Breast Cancer | rs111992639 | -0.6604 | 0.1093 | 1.69E-09 |
| CD28+ CD45RA- CD8dim AC | Breast Cancer | rs116499679 | 0.235 | 0.05229 | 7.23E-06 |
| CD28+ CD45RA- CD8dim AC | Breast Cancer | rs116884829 | 0.1788 | 0.03398 | 1.50E-07 |
| CD28+ CD45RA- CD8dim AC | Breast Cancer | rs12235775 | 0.1255 | 0.02537 | 7.91E-07 |
| CD28+ CD45RA- CD8dim AC | Breast Cancer | rs13231184 | -0.4138 | 0.09073 | 5.29E-06 |
| CD28+ CD45RA- CD8dim AC | Breast Cancer | rs138678985 | 0.2897 | 0.06184 | 2.92E-06 |
| CD28+ CD45RA- CD8dim AC | Breast Cancer | rs1450949 | 0.1131 | 0.02512 | 6.97E-06 |
| CD28+ CD45RA- CD8dim AC | Breast Cancer | rs2585739 | 0.2989 | 0.06454 | 3.78E-06 |
| CD28+ CD45RA- CD8dim AC | Breast Cancer | rs2670003 | -0.1279 | 0.0272 | 2.66E-06 |
| CD28+ CD45RA- CD8dim AC | Breast Cancer | rs28527279 | -0.1214 | 0.02643 | 4.54E-06 |
| CD28+ CD45RA- CD8dim AC | Breast Cancer | rs35117954 | -0.2546 | 0.05658 | 7.03E-06 |
| CD28+ CD45RA- CD8dim AC | Breast Cancer | rs35222020 | 0.3244 | 0.07107 | 5.19E-06 |
| CD28+ CD45RA- CD8dim AC | Breast Cancer | rs4129441 | -0.1161 | 0.02528 | 4.53E-06 |
| CD28+ CD45RA- CD8dim AC | Breast Cancer | rs565801 | -0.1068 | 0.02285 | 3.08E-06 |
| CD28+ CD45RA- CD8dim AC | Breast Cancer | rs61269136 | 0.2626 | 0.058 | 6.18E-06 |
| CD28+ CD45RA- CD8dim AC | Breast Cancer | rs687264 | -0.2594 | 0.05546 | 3.03E-06 |
| CD28- CD8br %CD8br | Breast Cancer | rs11544989 | 0.1078 | 0.02331 | 3.87E-06 |
| CD28- CD8br %CD8br | Breast Cancer | rs117279064 | -0.4812 | 0.1043 | 4.07E-06 |
| CD28- CD8br %CD8br | Breast Cancer | rs12450916 | 0.108 | 0.02273 | 2.11E-06 |
| CD28- CD8br %CD8br | Breast Cancer | rs139331134 | 0.2126 | 0.048 | 9.78E-06 |
| CD28- CD8br %CD8br | Breast Cancer | rs142216336 | 0.3436 | 0.06925 | 7.31E-07 |
| CD28- CD8br %CD8br | Breast Cancer | rs148460149 | -0.3239 | 0.06304 | 2.92E-07 |
| CD28- CD8br %CD8br | Breast Cancer | rs149359490 | -0.2474 | 0.0551 | 7.37E-06 |
| CD28- CD8br %CD8br | Breast Cancer | rs193125329 | -0.3709 | 0.07776 | 1.92E-06 |
| CD28- CD8br %CD8br | Breast Cancer | rs1978254 | 0.2113 | 0.04699 | 7.13E-06 |
| CD28- CD8br %CD8br | Breast Cancer | rs2275854 | 0.142 | 0.02743 | 2.40E-07 |
| CD28- CD8br %CD8br | Breast Cancer | rs34752208 | -0.1118 | 0.02423 | 4.08E-06 |
| CD28- CD8br %CD8br | Breast Cancer | rs4796089 | -0.2034 | 0.03346 | 1.35E-09 |
| CD28- CD8br %CD8br | Breast Cancer | rs56216385 | 0.1211 | 0.02399 | 4.74E-07 |
| CD28- CD8br %CD8br | Breast Cancer | rs57658648 | -0.2599 | 0.05531 | 2.73E-06 |
| CD28- CD8br %CD8br | Breast Cancer | rs57679712 | -0.1578 | 0.0352 | 7.56E-06 |
| CD28- CD8br %CD8br | Breast Cancer | rs62490686 | -0.1252 | 0.02805 | 8.34E-06 |
| CD28- CD8br %CD8br | Breast Cancer | rs62530389 | 0.1327 | 0.02954 | 7.25E-06 |
| CD28- CD8br %CD8br | Breast Cancer | rs669537 | -0.1092 | 0.02339 | 3.13E-06 |
| CD28- CD8br %CD8br | Breast Cancer | rs6781800 | 0.1308 | 0.02948 | 9.45E-06 |
| CD28- CD8br %CD8br | Breast Cancer | rs6904669 | -0.1458 | 0.02634 | 3.34E-08 |
| CD28- CD8br %CD8br | Breast Cancer | rs7728865 | -0.2169 | 0.03774 | 9.95E-09 |
| CD28- CD8br %CD8br | Breast Cancer | rs78240333 | 0.4293 | 0.09636 | 8.66E-06 |
| CD28- CD8br %CD8br | Breast Cancer | rs7989683 | -0.2218 | 0.04685 | 2.29E-06 |
| CD28- CD8br %CD8br | Breast Cancer | rs9468618 | -0.153 | 0.03449 | 9.46E-06 |
| CD28- DN (CD4-CD8-) %T cell | Breast Cancer | rs115953192 | -0.2826 | 0.06188 | 5.14E-06 |
| CD28- DN (CD4-CD8-) %T cell | Breast Cancer | rs117477265 | 0.3088 | 0.06891 | 7.68E-06 |
| CD28- DN (CD4-CD8-) %T cell | Breast Cancer | rs139249541 | 0.4291 | 0.07557 | 1.48E-08 |
| CD28- DN (CD4-CD8-) %T cell | Breast Cancer | rs140247402 | -0.2627 | 0.05866 | 7.76E-06 |
| CD28- DN (CD4-CD8-) %T cell | Breast Cancer | rs140444563 | 0.1863 | 0.04188 | 8.90E-06 |
| CD28- DN (CD4-CD8-) %T cell | Breast Cancer | rs142716558 | 0.1773 | 0.03883 | 5.15E-06 |
| CD28- DN (CD4-CD8-) %T cell | Breast Cancer | rs144346829 | 0.3042 | 0.06797 | 7.86E-06 |
| CD28- DN (CD4-CD8-) %T cell | Breast Cancer | rs1580182 | -0.2239 | 0.04791 | 3.08E-06 |
| CD28- DN (CD4-CD8-) %T cell | Breast Cancer | rs1875168 | -0.1431 | 0.03044 | 2.70E-06 |
| CD28- DN (CD4-CD8-) %T cell | Breast Cancer | rs2511751 | -0.1228 | 0.02595 | 2.31E-06 |
| CD28- DN (CD4-CD8-) %T cell | Breast Cancer | rs28383283 | -0.1593 | 0.03536 | 6.84E-06 |
| CD28- DN (CD4-CD8-) %T cell | Breast Cancer | rs36037322 | -0.14 | 0.03029 | 3.92E-06 |
| CD28- DN (CD4-CD8-) %T cell | Breast Cancer | rs66989520 | 0.1779 | 0.03737 | 2.00E-06 |
| CD28- DN (CD4-CD8-) %T cell | Breast Cancer | rs6755452 | 0.2159 | 0.03275 | 5.00E-11 |
| CD28- DN (CD4-CD8-) %T cell | Breast Cancer | rs6907169 | 0.2334 | 0.04707 | 7.39E-07 |
| CD28- DN (CD4-CD8-) %T cell | Breast Cancer | rs73560923 | 0.1771 | 0.03918 | 6.39E-06 |
| CD28- DN (CD4-CD8-) %T cell | Breast Cancer | rs76549873 | -0.5406 | 0.1168 | 3.80E-06 |
| CD28- DN (CD4-CD8-) %T cell | Breast Cancer | rs78936881 | -0.2123 | 0.04619 | 4.45E-06 |
| CD28- DN (CD4-CD8-) %T cell | Breast Cancer | rs79086781 | 0.3371 | 0.06742 | 6.03E-07 |
| CD28- DN (CD4-CD8-) %T cell | Breast Cancer | rs8077688 | 0.1485 | 0.03238 | 4.67E-06 |
| CD28- DN (CD4-CD8-) %T cell | Breast Cancer | rs8113259 | -0.2368 | 0.05213 | 5.77E-06 |
| CD28- DN (CD4-CD8-) %T cell | Breast Cancer | rs9488834 | 0.2727 | 0.06114 | 8.41E-06 |
| CD28- DN (CD4-CD8-) %T cell | Breast Cancer | rs958219 | -0.1255 | 0.02611 | 1.61E-06 |
| CD28- DN (CD4-CD8-) %T cell | Breast Cancer | rs9896155 | 0.1701 | 0.02733 | 5.51E-10 |
| CD24 on IgD+ CD38br | Breast Cancer | rs10056811 | -0.1322 | 0.02752 | 1.62E-06 |
| CD24 on IgD+ CD38br | Breast Cancer | rs10108273 | 0.1701 | 0.0373 | 5.28E-06 |
| CD24 on IgD+ CD38br | Breast Cancer | rs116032501 | -0.1879 | 0.04125 | 5.42E-06 |
| CD24 on IgD+ CD38br | Breast Cancer | rs117920360 | 0.1459 | 0.03267 | 8.28E-06 |
| CD24 on IgD+ CD38br | Breast Cancer | rs146379662 | 0.5365 | 0.1154 | 3.47E-06 |
| CD24 on IgD+ CD38br | Breast Cancer | rs16923647 | -0.164 | 0.03428 | 1.79E-06 |
| CD24 on IgD+ CD38br | Breast Cancer | rs1799693 | -0.2018 | 0.03253 | 6.12E-10 |
| CD24 on IgD+ CD38br | Breast Cancer | rs181036992 | -0.4375 | 0.091 | 1.59E-06 |
| CD24 on IgD+ CD38br | Breast Cancer | rs192325628 | 0.4876 | 0.09848 | 7.70E-07 |
| CD24 on IgD+ CD38br | Breast Cancer | rs3097667 | -0.1519 | 0.03161 | 1.61E-06 |
| CD24 on IgD+ CD38br | Breast Cancer | rs35093039 | -0.21 | 0.04748 | 9.99E-06 |
| CD24 on IgD+ CD38br | Breast Cancer | rs4368221 | 0.1558 | 0.03385 | 4.33E-06 |
| CD24 on IgD+ CD38br | Breast Cancer | rs45609135 | -0.1767 | 0.03959 | 8.31E-06 |
| CD24 on IgD+ CD38br | Breast Cancer | rs4765854 | -0.2274 | 0.05027 | 6.26E-06 |
| CD24 on IgD+ CD38br | Breast Cancer | rs57680652 | 0.128 | 0.02606 | 9.41E-07 |
| CD24 on IgD+ CD38br | Breast Cancer | rs59655785 | 0.1174 | 0.02474 | 2.18E-06 |
| CD24 on IgD+ CD38br | Breast Cancer | rs6666130 | -0.2436 | 0.05141 | 2.25E-06 |
| CD24 on IgD+ CD38br | Breast Cancer | rs75059545 | 0.1761 | 0.03786 | 3.39E-06 |
| CD24 on IgD+ CD38br | Breast Cancer | rs7781145 | -0.1421 | 0.0291 | 1.09E-06 |
| CD24 on IgD+ CD38br | Breast Cancer | rs9326867 | 0.1697 | 0.03713 | 5.04E-06 |
| CD24 on IgD+ CD38br | Breast Cancer | rs9398107 | -0.4386 | 0.0546 | 1.28E-15 |
| CD24 on IgD+ CD38br | Breast Cancer | rs9452529 | -0.5098 | 0.1073 | 2.09E-06 |
| IgD on unsw mem | Breast Cancer | rs11042077 | 0.1233 | 0.02525 | 1.09E-06 |
| IgD on unsw mem | Breast Cancer | rs115062208 | -0.6992 | 0.1209 | 7.97E-09 |
| IgD on unsw mem | Breast Cancer | rs12032479 | -0.1554 | 0.03281 | 2.27E-06 |
| IgD on unsw mem | Breast Cancer | rs147970068 | -0.537 | 0.1213 | 9.86E-06 |
| IgD on unsw mem | Breast Cancer | rs17179337 | 0.1195 | 0.02533 | 2.45E-06 |
| IgD on unsw mem | Breast Cancer | rs1878808 | -0.1139 | 0.02471 | 4.21E-06 |
| IgD on unsw mem | Breast Cancer | rs2516708 | 0.1331 | 0.02723 | 1.05E-06 |
| IgD on unsw mem | Breast Cancer | rs28393044 | 0.3099 | 0.0697 | 8.99E-06 |
| IgD on unsw mem | Breast Cancer | rs572542 | -0.3492 | 0.07317 | 1.89E-06 |
| IgD on unsw mem | Breast Cancer | rs6895789 | -0.1644 | 0.03492 | 2.60E-06 |
| IgD on unsw mem | Breast Cancer | rs72715691 | -0.4872 | 0.1087 | 7.54E-06 |
| IgD on unsw mem | Breast Cancer | rs76112278 | -0.7826 | 0.08987 | 4.58E-18 |
| IgD on unsw mem | Breast Cancer | rs76354299 | -0.3606 | 0.06977 | 2.48E-07 |
| IgD on unsw mem | Breast Cancer | rs79300919 | 0.3949 | 0.08781 | 7.10E-06 |
| IgD on unsw mem | Breast Cancer | rs9599916 | 0.1395 | 0.02975 | 2.83E-06 |
| HVEM on EM CD4+ | Breast Cancer | rs114805022 | -0.7777 | 0.173 | 7.63E-06 |
| HVEM on EM CD4+ | Breast Cancer | rs115176674 | -0.2329 | 0.05134 | 6.26E-06 |
| HVEM on EM CD4+ | Breast Cancer | rs116838007 | 0.6668 | 0.1453 | 4.88E-06 |
| HVEM on EM CD4+ | Breast Cancer | rs11758857 | -0.4723 | 0.1012 | 3.38E-06 |
| HVEM on EM CD4+ | Breast Cancer | rs117761782 | -0.4469 | 0.09389 | 2.17E-06 |
| HVEM on EM CD4+ | Breast Cancer | rs12053385 | -0.2366 | 0.05297 | 8.64E-06 |
| HVEM on EM CD4+ | Breast Cancer | rs1772154 | -0.2144 | 0.04552 | 2.76E-06 |
| HVEM on EM CD4+ | Breast Cancer | rs2002586 | -0.2163 | 0.04097 | 1.52E-07 |
| HVEM on EM CD4+ | Breast Cancer | rs2182176 | 0.2882 | 0.03927 | 3.86E-13 |
| HVEM on EM CD4+ | Breast Cancer | rs2959645 | 0.1935 | 0.04354 | 9.65E-06 |
| HVEM on EM CD4+ | Breast Cancer | rs4657680 | -0.1852 | 0.04078 | 6.16E-06 |
| HVEM on EM CD4+ | Breast Cancer | rs4803979 | -0.2273 | 0.05006 | 6.16E-06 |
| HVEM on EM CD4+ | Breast Cancer | rs557685 | -0.3261 | 0.06505 | 6.13E-07 |
| HVEM on EM CD4+ | Breast Cancer | rs61873707 | 0.5528 | 0.1182 | 3.22E-06 |
| HVEM on EM CD4+ | Breast Cancer | rs6882776 | 0.1889 | 0.04193 | 7.24E-06 |
| HVEM on EM CD4+ | Breast Cancer | rs73150361 | 0.4366 | 0.09612 | 6.10E-06 |
| HVEM on EM CD4+ | Breast Cancer | rs74257706 | -0.2898 | 0.06329 | 5.15E-06 |
| HVEM on EM CD4+ | Breast Cancer | rs7754903 | 0.3032 | 0.05775 | 1.79E-07 |
| HVEM on EM CD4+ | Breast Cancer | rs7864163 | 0.5627 | 0.124 | 6.19E-06 |
| CD127 on CD45RA- CD4 not Treg | Breast Cancer | rs112348392 | -0.4045 | 0.08672 | 3.24E-06 |
| CD127 on CD45RA- CD4 not Treg | Breast Cancer | rs12513323 | -0.2432 | 0.05334 | 5.33E-06 |
| CD127 on CD45RA- CD4 not Treg | Breast Cancer | rs12634099 | 0.1548 | 0.03293 | 2.70E-06 |
| CD127 on CD45RA- CD4 not Treg | Breast Cancer | rs140978083 | 0.352 | 0.07528 | 3.06E-06 |
| CD127 on CD45RA- CD4 not Treg | Breast Cancer | rs143650607 | 0.3859 | 0.08324 | 3.70E-06 |
| CD127 on CD45RA- CD4 not Treg | Breast Cancer | rs149514573 | 0.2994 | 0.06583 | 5.63E-06 |
| CD127 on CD45RA- CD4 not Treg | Breast Cancer | rs16899999 | -0.2507 | 0.04281 | 5.30E-09 |
| CD127 on CD45RA- CD4 not Treg | Breast Cancer | rs17064297 | 0.1268 | 0.02828 | 7.66E-06 |
| CD127 on CD45RA- CD4 not Treg | Breast Cancer | rs17739576 | 0.1447 | 0.0307 | 2.54E-06 |
| CD127 on CD45RA- CD4 not Treg | Breast Cancer | rs3848363 | -0.1271 | 0.02718 | 3.06E-06 |
| CD127 on CD45RA- CD4 not Treg | Breast Cancer | rs5999348 | 0.1392 | 0.0307 | 6.02E-06 |
| CD127 on CD45RA- CD4 not Treg | Breast Cancer | rs6038366 | -0.1298 | 0.02825 | 4.51E-06 |
| CD127 on CD45RA- CD4 not Treg | Breast Cancer | rs6703836 | 0.1447 | 0.03013 | 1.64E-06 |
| CD127 on CD45RA- CD4 not Treg | Breast Cancer | rs75904097 | 0.4371 | 0.09733 | 7.37E-06 |
| CD127 on CD45RA- CD4 not Treg | Breast Cancer | rs8081659 | 0.1262 | 0.0278 | 5.84E-06 |
| CD127 on CD28+ CD4+ | Breast Cancer | rs111717712 | -0.3607 | 0.07979 | 6.41E-06 |
| CD127 on CD28+ CD4+ | Breast Cancer | rs12138848 | 0.6181 | 0.1236 | 6.06E-07 |
| CD127 on CD28+ CD4+ | Breast Cancer | rs12513323 | -0.2663 | 0.05352 | 6.85E-07 |
| CD127 on CD28+ CD4+ | Breast Cancer | rs130970 | -0.1281 | 0.028 | 4.93E-06 |
| CD127 on CD28+ CD4+ | Breast Cancer | rs149514573 | 0.3029 | 0.06608 | 4.75E-06 |
| CD127 on CD28+ CD4+ | Breast Cancer | rs1551777 | -0.1363 | 0.03063 | 8.84E-06 |
| CD127 on CD28+ CD4+ | Breast Cancer | rs1573734 | -0.182 | 0.02858 | 2.20E-10 |
| CD127 on CD28+ CD4+ | Breast Cancer | rs16899999 | -0.2835 | 0.04291 | 4.67E-11 |
| CD127 on CD28+ CD4+ | Breast Cancer | rs17064297 | 0.1323 | 0.02839 | 3.29E-06 |
| CD127 on CD28+ CD4+ | Breast Cancer | rs17739576 | 0.1374 | 0.03083 | 8.58E-06 |
| CD127 on CD28+ CD4+ | Breast Cancer | rs28680958 | 0.1855 | 0.03217 | 8.94E-09 |
| CD127 on CD28+ CD4+ | Breast Cancer | rs2961796 | 0.1547 | 0.03107 | 6.76E-07 |
| CD127 on CD28+ CD4+ | Breast Cancer | rs4426311 | -0.1315 | 0.02944 | 8.24E-06 |
| CD127 on CD28+ CD4+ | Breast Cancer | rs55872368 | -0.1732 | 0.03829 | 6.31E-06 |
| CD127 on CD28+ CD4+ | Breast Cancer | rs72652037 | -0.2897 | 0.06326 | 4.83E-06 |
| CD127 on CD28+ CD4+ | Breast Cancer | rs75904097 | 0.4429 | 0.09771 | 6.05E-06 |
| CD127 on CD28+ CD4+ | Breast Cancer | rs78763541 | -0.241 | 0.05094 | 2.33E-06 |
| CD127 on CD28+ CD4+ | Breast Cancer | rs84125 | -0.1287 | 0.02893 | 9.03E-06 |
| FSC-A on NK | Breast Cancer | rs1006368 | 0.389 | 0.04003 | 5.08E-22 |
| FSC-A on NK | Breast Cancer | rs10919543 | -0.1509 | 0.02717 | 3.03E-08 |
| FSC-A on NK | Breast Cancer | rs111460281 | 0.2794 | 0.06138 | 5.54E-06 |
| FSC-A on NK | Breast Cancer | rs117893932 | 0.2818 | 0.06153 | 4.84E-06 |
| FSC-A on NK | Breast Cancer | rs12542554 | 0.2057 | 0.04354 | 2.40E-06 |
| FSC-A on NK | Breast Cancer | rs146765347 | -0.286 | 0.06255 | 5.01E-06 |
| FSC-A on NK | Breast Cancer | rs16970975 | -0.1698 | 0.03597 | 2.45E-06 |
| FSC-A on NK | Breast Cancer | rs1735091 | -0.1744 | 0.03795 | 4.50E-06 |
| FSC-A on NK | Breast Cancer | rs2774933 | 0.1363 | 0.0281 | 1.30E-06 |
| FSC-A on NK | Breast Cancer | rs34700818 | 0.133 | 0.03006 | 9.94E-06 |
| FSC-A on NK | Breast Cancer | rs55708007 | 0.3112 | 0.0648 | 1.65E-06 |
| FSC-A on NK | Breast Cancer | rs5768144 | -0.1937 | 0.04237 | 5.05E-06 |
| FSC-A on NK | Breast Cancer | rs73112545 | -0.139 | 0.02933 | 2.24E-06 |
| FSC-A on NK | Breast Cancer | rs78273995 | 0.1351 | 0.0302 | 8.00E-06 |
| FSC-A on NK | Breast Cancer | rs79678187 | -0.1634 | 0.0336 | 1.21E-06 |
| FSC-A on NK | Breast Cancer | rs9912354 | 0.1606 | 0.02681 | 2.34E-09 |
| HLA DR on CD14+ CD16- monocyte | Breast Cancer | rs113337372 | -0.3065 | 0.06892 | 8.97E-06 |
| HLA DR on CD14+ CD16- monocyte | Breast Cancer | rs113555953 | 0.5125 | 0.1129 | 5.86E-06 |
| HLA DR on CD14+ CD16- monocyte | Breast Cancer | rs11788133 | -0.1567 | 0.03359 | 3.20E-06 |
| HLA DR on CD14+ CD16- monocyte | Breast Cancer | rs143858253 | 0.1754 | 0.039 | 7.12E-06 |
| HLA DR on CD14+ CD16- monocyte | Breast Cancer | rs146479897 | 0.4693 | 0.1013 | 3.72E-06 |
| HLA DR on CD14+ CD16- monocyte | Breast Cancer | rs148974843 | 0.2723 | 0.05871 | 3.64E-06 |
| HLA DR on CD14+ CD16- monocyte | Breast Cancer | rs150649461 | -0.2944 | 0.06482 | 5.77E-06 |
| HLA DR on CD14+ CD16- monocyte | Breast Cancer | rs1800973 | -0.3123 | 0.04579 | 1.06E-11 |
| HLA DR on CD14+ CD16- monocyte | Breast Cancer | rs188394267 | -0.2065 | 0.04611 | 7.72E-06 |
| HLA DR on CD14+ CD16- monocyte | Breast Cancer | rs241432 | 0.2595 | 0.02732 | 3.75E-21 |
| HLA DR on CD14+ CD16- monocyte | Breast Cancer | rs3130396 | -0.149 | 0.02972 | 5.58E-07 |
| HLA DR on CD14+ CD16- monocyte | Breast Cancer | rs35848415 | -0.274 | 0.05943 | 4.14E-06 |
| HLA DR on CD14+ CD16- monocyte | Breast Cancer | rs62005962 | -0.1224 | 0.02703 | 6.10E-06 |
| HLA DR on CD14+ CD16- monocyte | Breast Cancer | rs62399337 | -0.2547 | 0.05493 | 3.66E-06 |
| HLA DR on CD14+ CD16- monocyte | Breast Cancer | rs74803828 | 0.1783 | 0.03997 | 8.47E-06 |
| HLA DR on CD14+ CD16- monocyte | Breast Cancer | rs80032720 | 0.4253 | 0.0545 | 7.76E-15 |
| HLA DR on CD14+ CD16- monocyte | Breast Cancer | rs8043889 | -0.1291 | 0.0272 | 2.16E-06 |
| HLA DR on CD14+ CD16- monocyte | Breast Cancer | rs9270585 | -0.6054 | 0.0279 | 3.04E-98 |
| HLA DR on CD14+ CD16- monocyte | Breast Cancer | rs9271768 | -0.5252 | 0.02936 | 1.21E-68 |
| CD11c on myeloid DC | Breast Cancer | rs11004890 | 0.1239 | 0.02786 | 9.01E-06 |
| CD11c on myeloid DC | Breast Cancer | rs113167560 | -0.3213 | 0.06762 | 2.12E-06 |
| CD11c on myeloid DC | Breast Cancer | rs113404848 | 0.2758 | 0.05575 | 7.96E-07 |
| CD11c on myeloid DC | Breast Cancer | rs11708131 | -0.2627 | 0.05339 | 9.12E-07 |
| CD11c on myeloid DC | Breast Cancer | rs13255633 | 0.1629 | 0.03496 | 3.31E-06 |
| CD11c on myeloid DC | Breast Cancer | rs16881169 | 0.2616 | 0.05889 | 9.26E-06 |
| CD11c on myeloid DC | Breast Cancer | rs17481824 | 0.1773 | 0.03875 | 4.96E-06 |
| CD11c on myeloid DC | Breast Cancer | rs2541232 | 0.1224 | 0.02695 | 5.77E-06 |
| CD11c on myeloid DC | Breast Cancer | rs35945038 | -0.1261 | 0.02762 | 5.18E-06 |
| CD11c on myeloid DC | Breast Cancer | rs4770411 | 0.1622 | 0.03224 | 5.17E-07 |
| CD11c on myeloid DC | Breast Cancer | rs56116087 | -0.2078 | 0.04419 | 2.69E-06 |
| CD11c on myeloid DC | Breast Cancer | rs56210513 | -0.5557 | 0.1234 | 6.90E-06 |
| CD11c on myeloid DC | Breast Cancer | rs61802329 | 0.1669 | 0.02718 | 9.48E-10 |
| CD11c on myeloid DC | Breast Cancer | rs67898294 | 0.3276 | 0.02803 | 7.19E-31 |
| CD11c on myeloid DC | Breast Cancer | rs71652355 | 0.3556 | 0.07661 | 3.62E-06 |
| CD11c on myeloid DC | Breast Cancer | rs72662658 | 0.5005 | 0.09331 | 8.80E-08 |
| CD11c on myeloid DC | Breast Cancer | rs72732223 | 0.2036 | 0.04558 | 8.27E-06 |
| CD11c on myeloid DC | Breast Cancer | rs72873714 | 0.2715 | 0.06014 | 6.61E-06 |
| CD11c on myeloid DC | Breast Cancer | rs75841521 | -0.442 | 0.08067 | 4.65E-08 |
| CD11c on myeloid DC | Breast Cancer | rs778233 | -0.1335 | 0.0273 | 1.06E-06 |
| CD11c on myeloid DC | Breast Cancer | rs7795869 | 0.1616 | 0.03467 | 3.27E-06 |
| CD11c on myeloid DC | Breast Cancer | rs78520150 | 0.3947 | 0.07143 | 3.57E-08 |
| CD11c on myeloid DC | Breast Cancer | rs7869020 | -0.2428 | 0.05031 | 1.46E-06 |
| CD11c on myeloid DC | Breast Cancer | rs79298699 | -0.279 | 0.04807 | 7.22E-09 |
| CD11c on myeloid DC | Breast Cancer | rs79355481 | 0.383 | 0.0736 | 2.08E-07 |
| CD11c on myeloid DC | Breast Cancer | rs80318951 | -0.1501 | 0.02905 | 2.54E-07 |
| CD11c on myeloid DC | Breast Cancer | rs9562128 | 0.1343 | 0.02956 | 5.80E-06 |
| CD45RA on naive CD4+ | Breast Cancer | rs10022333 | -0.1337 | 0.02982 | 7.58E-06 |
| CD45RA on naive CD4+ | Breast Cancer | rs113885925 | 0.4252 | 0.09388 | 6.16E-06 |
| CD45RA on naive CD4+ | Breast Cancer | rs115645435 | 0.4693 | 0.1042 | 6.92E-06 |
| CD45RA on naive CD4+ | Breast Cancer | rs11589644 | 0.5402 | 0.1165 | 3.69E-06 |
| CD45RA on naive CD4+ | Breast Cancer | rs12730924 | 0.1271 | 0.02865 | 9.45E-06 |
| CD45RA on naive CD4+ | Breast Cancer | rs138283413 | 0.3299 | 0.0697 | 2.32E-06 |
| CD45RA on naive CD4+ | Breast Cancer | rs139544135 | 0.3938 | 0.08713 | 6.46E-06 |
| CD45RA on naive CD4+ | Breast Cancer | rs145947216 | 0.7147 | 0.07055 | 9.98E-24 |
| CD45RA on naive CD4+ | Breast Cancer | rs147692482 | 0.6089 | 0.08426 | 6.32E-13 |
| CD45RA on naive CD4+ | Breast Cancer | rs1641548 | 0.176 | 0.03597 | 1.05E-06 |
| CD45RA on naive CD4+ | Breast Cancer | rs182240059 | 0.6602 | 0.07094 | 2.53E-20 |
| CD45RA on naive CD4+ | Breast Cancer | rs28590272 | 0.1304 | 0.02937 | 9.36E-06 |
| CD45RA on naive CD4+ | Breast Cancer | rs34558770 | 0.4963 | 0.03241 | 5.75E-51 |
| CD45RA on naive CD4+ | Breast Cancer | rs35550194 | 0.3391 | 0.06779 | 6.00E-07 |
| CD45RA on naive CD4+ | Breast Cancer | rs35592432 | -0.4046 | 0.08034 | 5.05E-07 |
| CD45RA on naive CD4+ | Breast Cancer | rs531042 | -0.1404 | 0.03111 | 6.65E-06 |
| CD45RA on naive CD4+ | Breast Cancer | rs55733272 | 0.1463 | 0.0328 | 8.47E-06 |
| CD45RA on naive CD4+ | Breast Cancer | rs61913600 | 0.1272 | 0.0278 | 4.92E-06 |
| CD45RA on naive CD4+ | Breast Cancer | rs61938012 | -0.1286 | 0.02895 | 9.22E-06 |
| CD45RA on naive CD4+ | Breast Cancer | rs62328019 | 0.2505 | 0.05636 | 9.15E-06 |
| CD45RA on naive CD4+ | Breast Cancer | rs72859264 | -0.4805 | 0.108 | 8.93E-06 |
| CD45RA on naive CD4+ | Breast Cancer | rs75425544 | -0.4207 | 0.09318 | 6.59E-06 |
| CD45RA on naive CD4+ | Breast Cancer | rs7605193 | -0.3245 | 0.05318 | 1.19E-09 |
| CD45RA on naive CD4+ | Breast Cancer | rs76156778 | 0.6246 | 0.1023 | 1.15E-09 |
| CD45RA on naive CD4+ | Breast Cancer | rs79000121 | -0.3227 | 0.0645 | 5.96E-07 |
| CD45RA on naive CD4+ | Breast Cancer | rs79505210 | 0.4859 | 0.09477 | 3.13E-07 |
| CD45RA on naive CD4+ | Breast Cancer | rs79747818 | 0.3184 | 0.0526 | 1.59E-09 |
| CD45RA on naive CD4+ | Breast Cancer | rs917116 | 0.1762 | 0.0299 | 4.26E-09 |
| CD45RA on naive CD4+ | Breast Cancer | rs9834739 | -0.3956 | 0.08788 | 7.02E-06 |
| CD4RA on TD CD4+ | Breast Cancer | rs10022333 | -0.1337 | 0.02982 | 7.58E-06 |
| CD4RA on TD CD4+ | Breast Cancer | rs113885925 | 0.4252 | 0.09388 | 6.16E-06 |
| CD4RA on TD CD4+ | Breast Cancer | rs115645435 | 0.4693 | 0.1042 | 6.92E-06 |
| CD4RA on TD CD4+ | Breast Cancer | rs11589644 | 0.5402 | 0.1165 | 3.69E-06 |
| CD4RA on TD CD4+ | Breast Cancer | rs12730924 | 0.1271 | 0.02865 | 9.45E-06 |
| CD4RA on TD CD4+ | Breast Cancer | rs138283413 | 0.3299 | 0.0697 | 2.32E-06 |
| CD4RA on TD CD4+ | Breast Cancer | rs139544135 | 0.3938 | 0.08713 | 6.46E-06 |
| CD4RA on TD CD4+ | Breast Cancer | rs145947216 | 0.7147 | 0.07055 | 9.98E-24 |
| CD4RA on TD CD4+ | Breast Cancer | rs147692482 | 0.6089 | 0.08426 | 6.32E-13 |
| CD4RA on TD CD4+ | Breast Cancer | rs1641548 | 0.176 | 0.03597 | 1.05E-06 |
| CD4RA on TD CD4+ | Breast Cancer | rs182240059 | 0.6602 | 0.07094 | 2.53E-20 |
| CD4RA on TD CD4+ | Breast Cancer | rs28590272 | 0.1304 | 0.02937 | 9.36E-06 |
| CD4RA on TD CD4+ | Breast Cancer | rs34558770 | 0.4963 | 0.03241 | 5.75E-51 |
| CD4RA on TD CD4+ | Breast Cancer | rs35550194 | 0.3391 | 0.06779 | 6.00E-07 |
| CD4RA on TD CD4+ | Breast Cancer | rs35592432 | -0.4046 | 0.08034 | 5.05E-07 |
| CD4RA on TD CD4+ | Breast Cancer | rs531042 | -0.1404 | 0.03111 | 6.65E-06 |
| CD4RA on TD CD4+ | Breast Cancer | rs55733272 | 0.1463 | 0.0328 | 8.47E-06 |
| CD4RA on TD CD4+ | Breast Cancer | rs61913600 | 0.1272 | 0.0278 | 4.92E-06 |
| CD4RA on TD CD4+ | Breast Cancer | rs61938012 | -0.1286 | 0.02895 | 9.22E-06 |
| CD4RA on TD CD4+ | Breast Cancer | rs62328019 | 0.2505 | 0.05636 | 9.15E-06 |
| CD4RA on TD CD4+ | Breast Cancer | rs72859264 | -0.4805 | 0.108 | 8.93E-06 |
| CD4RA on TD CD4+ | Breast Cancer | rs75425544 | -0.4207 | 0.09318 | 6.59E-06 |
| CD4RA on TD CD4+ | Breast Cancer | rs7605193 | -0.3245 | 0.05318 | 1.19E-09 |
| CD4RA on TD CD4+ | Breast Cancer | rs76156778 | 0.6246 | 0.1023 | 1.15E-09 |
| CD4RA on TD CD4+ | Breast Cancer | rs79000121 | -0.3227 | 0.0645 | 5.96E-07 |
| CD4RA on TD CD4+ | Breast Cancer | rs79505210 | 0.4859 | 0.09477 | 3.13E-07 |
| CD4RA on TD CD4+ | Breast Cancer | rs79747818 | 0.3184 | 0.0526 | 1.59E-09 |
| CD4RA on TD CD4+ | Breast Cancer | rs917116 | 0.1762 | 0.0299 | 4.26E-09 |
| CD4RA on TD CD4+ | Breast Cancer | rs9834739 | -0.3956 | 0.08788 | 7.02E-06 |
| HLA DR on CD33br HLA DR+ CD14- | Breast Cancer | rs112096735 | -0.286 | 0.06037 | 2.36E-06 |
| HLA DR on CD33br HLA DR+ CD14- | Breast Cancer | rs116007826 | 0.6643 | 0.07716 | 1.75E-17 |
| HLA DR on CD33br HLA DR+ CD14- | Breast Cancer | rs1424773 | -0.1924 | 0.04154 | 3.95E-06 |
| HLA DR on CD33br HLA DR+ CD14- | Breast Cancer | rs146788953 | -0.481 | 0.1005 | 1.86E-06 |
| HLA DR on CD33br HLA DR+ CD14- | Breast Cancer | rs147951036 | -0.7396 | 0.1516 | 1.17E-06 |
| HLA DR on CD33br HLA DR+ CD14- | Breast Cancer | rs16987905 | -0.2637 | 0.05636 | 3.15E-06 |
| HLA DR on CD33br HLA DR+ CD14- | Breast Cancer | rs186879638 | 0.4693 | 0.09858 | 2.10E-06 |
| HLA DR on CD33br HLA DR+ CD14- | Breast Cancer | rs238873 | 0.5614 | 0.1033 | 6.26E-08 |
| HLA DR on CD33br HLA DR+ CD14- | Breast Cancer | rs36037794 | 0.4846 | 0.1091 | 9.49E-06 |
| HLA DR on CD33br HLA DR+ CD14- | Breast Cancer | rs523727 | -0.3524 | 0.04389 | 1.89E-15 |
| HLA DR on CD33br HLA DR+ CD14- | Breast Cancer | rs57623845 | -0.2111 | 0.04284 | 9.25E-07 |
| HLA DR on CD33br HLA DR+ CD14- | Breast Cancer | rs6925683 | 0.2904 | 0.05585 | 2.25E-07 |
| HLA DR on CD33br HLA DR+ CD14- | Breast Cancer | rs71363011 | -0.3545 | 0.07906 | 7.84E-06 |
| HLA DR on CD33br HLA DR+ CD14- | Breast Cancer | rs71600299 | -0.1834 | 0.0401 | 5.16E-06 |
| HLA DR on CD33br HLA DR+ CD14- | Breast Cancer | rs77506340 | 0.4117 | 0.09279 | 9.74E-06 |
| HLA DR on CD33br HLA DR+ CD14- | Breast Cancer | rs847842 | 0.2527 | 0.05585 | 6.52E-06 |
| HLA DR on CD33br HLA DR+ CD14- | Breast Cancer | rs9270588 | -0.5992 | 0.03915 | 2.14E-49 |
|  |  |  |  |  |  |
|  |  |  |  |  |  |
| **SNP for MR analysis between Gut microbiota and Mediator** | | | | | |
| Exposure | Outcome | SNP | beta | Se | Pvalue |
| Genus An7 | CD11c on myeloid DC | rs1016579 | 0.0817956 | 0.0181092 | 6.30E-06 |
| Genus An7 | CD11c on myeloid DC | rs118162561 | 0.0961169 | 0.020823 | 3.90E-06 |
| Genus An7 | CD11c on myeloid DC | rs139195374 | 0.0655593 | 0.0146199 | 7.30E-06 |
| Genus An7 | CD11c on myeloid DC | rs143347000 | 0.0574873 | 0.012811 | 7.20E-06 |
| Genus An7 | CD11c on myeloid DC | rs145171932 | 0.123477 | 0.0225103 | 4.10E-08 |
| Genus An7 | CD11c on myeloid DC | rs17258709 | -0.0480784 | 0.0105296 | 5.00E-06 |
| Genus An7 | CD11c on myeloid DC | rs2283177 | 0.0714829 | 0.0155977 | 4.60E-06 |
| Genus An7 | CD11c on myeloid DC | rs2797887 | 0.119365 | 0.0265818 | 7.10E-06 |
| Genus An7 | CD11c on myeloid DC | rs2852623 | -0.0556296 | 0.0111079 | 5.50E-07 |
| Genus An7 | CD11c on myeloid DC | rs3911603 | -0.0438764 | 0.00992335 | 9.80E-06 |
| Genus An7 | CD11c on myeloid DC | rs4674463 | -0.0710934 | 0.0143534 | 7.30E-07 |
| Genus An7 | CD11c on myeloid DC | rs4722327 | 0.034588 | 0.00738657 | 2.80E-06 |
| Genus An7 | CD11c on myeloid DC | rs61742849 | 0.137142 | 0.0267657 | 3.00E-07 |
| Genus An7 | CD11c on myeloid DC | rs7043204 | 0.0414647 | 0.00909919 | 5.20E-06 |
| Genus An7 | CD11c on myeloid DC | rs76928381 | 0.153177 | 0.0290407 | 1.30E-07 |
| Species Bacteroides A plebeius A | CD127 on CD45RA- CD4 not Treg | rs10510731 | 0.112354 | 0.0224698 | 5.70E-07 |
| Species Bacteroides A plebeius A | CD127 on CD45RA- CD4 not Treg | rs11193092 | 0.239782 | 0.0539899 | 8.90E-06 |
| Species Bacteroides A plebeius A | CD127 on CD45RA- CD4 not Treg | rs12314696 | 0.195811 | 0.0431981 | 5.80E-06 |
| Species Bacteroides A plebeius A | CD127 on CD45RA- CD4 not Treg | rs12436076 | -0.121437 | 0.0267624 | 5.70E-06 |
| Species Bacteroides A plebeius A | CD127 on CD45RA- CD4 not Treg | rs12885338 | -0.0843342 | 0.0189146 | 8.20E-06 |
| Species Bacteroides A plebeius A | CD127 on CD45RA- CD4 not Treg | rs139389953 | 0.466232 | 0.0826217 | 1.70E-08 |
| Species Bacteroides A plebeius A | CD127 on CD45RA- CD4 not Treg | rs145791346 | 0.309116 | 0.069153 | 7.80E-06 |
| Species Bacteroides A plebeius A | CD127 on CD45RA- CD4 not Treg | rs149561196 | 0.273343 | 0.0562642 | 1.20E-06 |
| Species Bacteroides A plebeius A | CD127 on CD45RA- CD4 not Treg | rs34760623 | 0.245941 | 0.0513769 | 1.70E-06 |
| Species Bacteroides A plebeius A | CD127 on CD45RA- CD4 not Treg | rs3748487 | 0.236002 | 0.0520699 | 5.80E-06 |
| Species Bacteroides A plebeius A | CD127 on CD45RA- CD4 not Treg | rs3754935 | 0.169694 | 0.0377568 | 7.00E-06 |
| Species Bacteroides A plebeius A | CD127 on CD45RA- CD4 not Treg | rs57197355 | -0.123645 | 0.0272504 | 5.70E-06 |
| Species Bacteroides A plebeius A | CD127 on CD45RA- CD4 not Treg | rs61269211 | 0.125397 | 0.0257311 | 1.10E-06 |
| Species Bacteroides A plebeius A | CD127 on CD45RA- CD4 not Treg | rs61881773 | 0.254248 | 0.054798 | 3.50E-06 |
| Species Bacteroides A plebeius A | CD127 on CD45RA- CD4 not Treg | rs7179783 | 0.170501 | 0.0378988 | 6.80E-06 |
| Species Bacteroides A plebeius A | CD127 on CD45RA- CD4 not Treg | rs73113075 | -0.243542 | 0.054858 | 9.00E-06 |
| Species Bacteroides A plebeius A | CD127 on CD45RA- CD4 not Treg | rs73594223 | 0.28694 | 0.0596332 | 1.50E-06 |
| Species Bacteroides A plebeius A | CD127 on CD45RA- CD4 not Treg | rs77969904 | 0.274313 | 0.0590701 | 3.40E-06 |
| Species Bacteroides A plebeius A | CD127 on CD45RA- CD4 not Treg | rs7927877 | 0.0959895 | 0.020696 | 3.50E-06 |
| Species Bacteroides A plebeius A | CD127 on CD45RA- CD4 not Treg | rs9759894 | 0.100905 | 0.0216277 | 3.10E-06 |
| Family Fibrobacteraceae | CD127 on CD28+ CD4+ | rs11040652 | 0.0131414 | 0.00296249 | 9.20E-06 |
| Family Fibrobacteraceae | CD127 on CD28+ CD5+ | rs11129117 | 0.0139802 | 0.00305237 | 4.60E-06 |
| Family Fibrobacteraceae | CD127 on CD28+ CD6+ | rs112448126 | 0.0456544 | 0.00904268 | 4.40E-07 |
| Family Fibrobacteraceae | CD127 on CD28+ CD7+ | rs12543777 | 0.0134018 | 0.00298661 | 7.20E-06 |
| Family Fibrobacteraceae | CD127 on CD28+ CD8+ | rs13391970 | -0.0245971 | 0.00525995 | 2.90E-06 |
| Family Fibrobacteraceae | CD127 on CD28+ CD9+ | rs144057235 | 0.056383 | 0.0124256 | 5.70E-06 |
| Family Fibrobacteraceae | CD127 on CD28+ CD10+ | rs17183864 | 0.0520869 | 0.0112418 | 3.60E-06 |
| Family Fibrobacteraceae | CD127 on CD28+ CD11+ | rs2418034 | -0.0178498 | 0.0035847 | 6.40E-07 |
| Family Fibrobacteraceae | CD127 on CD28+ CD12+ | rs34832380 | 0.0427599 | 0.00949516 | 6.70E-06 |
| Family Fibrobacteraceae | CD127 on CD28+ CD13+ | rs4783644 | -0.0165962 | 0.00366164 | 5.80E-06 |
| Family Fibrobacteraceae | CD127 on CD28+ CD14+ | rs56278671 | 0.0595988 | 0.0134351 | 9.20E-06 |
| Family Fibrobacteraceae | CD127 on CD28+ CD15+ | rs7138143 | -0.0158182 | 0.00308459 | 2.90E-07 |
| Family Fibrobacteraceae | CD127 on CD28+ CD16+ | rs7250654 | 0.0240002 | 0.00479289 | 5.50E-07 |
| Family Fibrobacteraceae | CD127 on CD28+ CD17+ | rs753897 | -0.0146198 | 0.00315518 | 3.60E-06 |
| Family Fibrobacteraceae | CD127 on CD28+ CD18+ | rs76620736 | 0.0183326 | 0.00411456 | 8.40E-06 |
| Family Fibrobacteraceae | CD127 on CD28+ CD19+ | rs9890225 | -0.0130659 | 0.00294692 | 9.30E-06 |
| Species Megamonas funiformis | CD38 on IgD+ CD24- | rs10025256 | 0.0662322 | 0.0147327 | 6.90E-06 |
| Species Megamonas funiformis | CD38 on IgD+ CD24- | rs10196495 | 0.0490404 | 0.0107514 | 5.10E-06 |
| Species Megamonas funiformis | CD38 on IgD+ CD24- | rs112910828 | 0.149515 | 0.031759 | 2.50E-06 |
| Species Megamonas funiformis | CD38 on IgD+ CD24- | rs114894604 | 0.14832 | 0.0312218 | 2.00E-06 |
| Species Megamonas funiformis | CD38 on IgD+ CD24- | rs116338908 | 0.236574 | 0.0520765 | 5.60E-06 |
| Species Megamonas funiformis | CD38 on IgD+ CD24- | rs116895877 | 0.20284 | 0.0444071 | 4.90E-06 |
| Species Megamonas funiformis | CD38 on IgD+ CD24- | rs117243639 | 0.227846 | 0.0436466 | 1.80E-07 |
| Species Megamonas funiformis | CD38 on IgD+ CD24- | rs117992479 | 0.243606 | 0.0490238 | 6.70E-07 |
| Species Megamonas funiformis | CD38 on IgD+ CD24- | rs12491882 | 0.0491718 | 0.010936 | 6.90E-06 |
| Species Megamonas funiformis | CD38 on IgD+ CD24- | rs138309059 | 0.1929 | 0.0424601 | 5.50E-06 |
| Species Megamonas funiformis | CD38 on IgD+ CD24- | rs139556348 | 0.168807 | 0.0367688 | 4.40E-06 |
| Species Megamonas funiformis | CD38 on IgD+ CD24- | rs139616585 | 0.22327 | 0.042319 | 1.30E-07 |
| Species Megamonas funiformis | CD38 on IgD+ CD24- | rs149221610 | 0.193402 | 0.0428127 | 6.30E-06 |
| Species Megamonas funiformis | CD38 on IgD+ CD24- | rs17456563 | 0.129309 | 0.0279236 | 3.60E-06 |
| Species Megamonas funiformis | CD38 on IgD+ CD24- | rs1821595 | 0.192357 | 0.0427188 | 6.70E-06 |
| Species Megamonas funiformis | CD38 on IgD+ CD24- | rs190157640 | 0.213602 | 0.0478313 | 8.00E-06 |
| Species Megamonas funiformis | CD38 on IgD+ CD24- | rs1999144 | 0.112175 | 0.0228878 | 9.50E-07 |
| Species Megamonas funiformis | CD38 on IgD+ CD24- | rs2451340 | 0.14932 | 0.0311923 | 1.70E-06 |
| Species Megamonas funiformis | CD38 on IgD+ CD24- | rs35481789 | 0.0543384 | 0.0121015 | 7.10E-06 |
| Species Megamonas funiformis | CD38 on IgD+ CD24- | rs36112940 | 0.124939 | 0.0251967 | 7.10E-07 |
| Species Megamonas funiformis | CD38 on IgD+ CD24- | rs3783925 | -0.0510338 | 0.0111127 | 4.40E-06 |
| Species Megamonas funiformis | CD38 on IgD+ CD24- | rs4658309 | -0.0505118 | 0.0114103 | 9.60E-06 |
| Species Megamonas funiformis | CD38 on IgD+ CD24- | rs56009508 | 0.220364 | 0.0461912 | 1.80E-06 |
| Species Megamonas funiformis | CD38 on IgD+ CD24- | rs56293483 | 0.0670958 | 0.0150349 | 8.10E-06 |
| Species Megamonas funiformis | CD38 on IgD+ CD24- | rs58277973 | 0.121583 | 0.0270259 | 6.80E-06 |
| Species Megamonas funiformis | CD38 on IgD+ CD24- | rs6976718 | 0.0487653 | 0.0106901 | 5.10E-06 |
| Species Megamonas funiformis | CD38 on IgD+ CD24- | rs71539626 | 0.223808 | 0.0494546 | 6.00E-06 |
| Species Megamonas funiformis | CD38 on IgD+ CD24- | rs73789361 | 0.146333 | 0.0321678 | 5.40E-06 |
| Species Megamonas funiformis | CD38 on IgD+ CD24- | rs75550369 | 0.22381 | 0.0482229 | 3.50E-06 |
| Species Megamonas funiformis | CD38 on IgD+ CD24- | rs75824665 | 0.168225 | 0.0370359 | 5.60E-06 |
| Species Megamonas funiformis | CD38 on IgD+ CD24- | rs76400051 | 0.0816296 | 0.0179743 | 5.60E-06 |
| Species Megamonas funiformis | CD38 on IgD+ CD24- | rs77458346 | 0.180514 | 0.0398821 | 6.00E-06 |
| Species Megamonas funiformis | CD38 on IgD+ CD24- | rs78393153 | 0.23191 | 0.0418813 | 3.10E-08 |
| Species Megamonas funiformis | CD38 on IgD+ CD24- | rs79498746 | 0.121598 | 0.0257342 | 2.30E-06 |
| Species Megamonas funiformis | CD38 on IgD+ CD24- | rs79605300 | 0.176044 | 0.0397788 | 9.60E-06 |
| Species Megamonas funiformis | CD38 on IgD+ CD24- | rs79747352 | 0.181793 | 0.0366964 | 7.30E-07 |
| Species Megamonas funiformis | CD38 on IgD+ CD24- | rs908047 | 0.0572192 | 0.0123284 | 3.50E-06 |
| Species Megamonas funiformis | HVEM on EM CD4+ | rs10025256 | 0.0662322 | 0.0147327 | 6.90E-06 |
| Species Megamonas funiformis | HVEM on EM CD4+ | rs10196495 | 0.0490404 | 0.0107514 | 5.10E-06 |
| Species Megamonas funiformis | HVEM on EM CD4+ | rs112910828 | 0.149515 | 0.031759 | 2.50E-06 |
| Species Megamonas funiformis | HVEM on EM CD4+ | rs114894604 | 0.14832 | 0.0312218 | 2.00E-06 |
| Species Megamonas funiformis | HVEM on EM CD4+ | rs116338908 | 0.236574 | 0.0520765 | 5.60E-06 |
| Species Megamonas funiformis | HVEM on EM CD4+ | rs116895877 | 0.20284 | 0.0444071 | 4.90E-06 |
| Species Megamonas funiformis | HVEM on EM CD4+ | rs117243639 | 0.227846 | 0.0436466 | 1.80E-07 |
| Species Megamonas funiformis | HVEM on EM CD4+ | rs117992479 | 0.243606 | 0.0490238 | 6.70E-07 |
| Species Megamonas funiformis | HVEM on EM CD4+ | rs12491882 | 0.0491718 | 0.010936 | 6.90E-06 |
| Species Megamonas funiformis | HVEM on EM CD4+ | rs138309059 | 0.1929 | 0.0424601 | 5.50E-06 |
| Species Megamonas funiformis | HVEM on EM CD4+ | rs139556348 | 0.168807 | 0.0367688 | 4.40E-06 |
| Species Megamonas funiformis | HVEM on EM CD4+ | rs139616585 | 0.22327 | 0.042319 | 1.30E-07 |
| Species Megamonas funiformis | HVEM on EM CD4+ | rs149221610 | 0.193402 | 0.0428127 | 6.30E-06 |
| Species Megamonas funiformis | HVEM on EM CD4+ | rs17456563 | 0.129309 | 0.0279236 | 3.60E-06 |
| Species Megamonas funiformis | HVEM on EM CD4+ | rs1821595 | 0.192357 | 0.0427188 | 6.70E-06 |
| Species Megamonas funiformis | HVEM on EM CD4+ | rs190157640 | 0.213602 | 0.0478313 | 8.00E-06 |
| Species Megamonas funiformis | HVEM on EM CD4+ | rs1999144 | 0.112175 | 0.0228878 | 9.50E-07 |
| Species Megamonas funiformis | HVEM on EM CD4+ | rs2451340 | 0.14932 | 0.0311923 | 1.70E-06 |
| Species Megamonas funiformis | HVEM on EM CD4+ | rs35481789 | 0.0543384 | 0.0121015 | 7.10E-06 |
| Species Megamonas funiformis | HVEM on EM CD4+ | rs36112940 | 0.124939 | 0.0251967 | 7.10E-07 |
| Species Megamonas funiformis | HVEM on EM CD4+ | rs3783925 | -0.0510338 | 0.0111127 | 4.40E-06 |
| Species Megamonas funiformis | HVEM on EM CD4+ | rs4658309 | -0.0505118 | 0.0114103 | 9.60E-06 |
| Species Megamonas funiformis | HVEM on EM CD4+ | rs56009508 | 0.220364 | 0.0461912 | 1.80E-06 |
| Species Megamonas funiformis | HVEM on EM CD4+ | rs56293483 | 0.0670958 | 0.0150349 | 8.10E-06 |
| Species Megamonas funiformis | HVEM on EM CD4+ | rs58277973 | 0.121583 | 0.0270259 | 6.80E-06 |
| Species Megamonas funiformis | HVEM on EM CD4+ | rs6976718 | 0.0487653 | 0.0106901 | 5.10E-06 |
| Species Megamonas funiformis | HVEM on EM CD4+ | rs71539626 | 0.223808 | 0.0494546 | 6.00E-06 |
| Species Megamonas funiformis | HVEM on EM CD4+ | rs73789361 | 0.146333 | 0.0321678 | 5.40E-06 |
| Species Megamonas funiformis | HVEM on EM CD4+ | rs75550369 | 0.22381 | 0.0482229 | 3.50E-06 |
| Species Megamonas funiformis | HVEM on EM CD4+ | rs75824665 | 0.168225 | 0.0370359 | 5.60E-06 |
| Species Megamonas funiformis | HVEM on EM CD4+ | rs76400051 | 0.0816296 | 0.0179743 | 5.60E-06 |
| Species Megamonas funiformis | HVEM on EM CD4+ | rs77458346 | 0.180514 | 0.0398821 | 6.00E-06 |
| Species Megamonas funiformis | HVEM on EM CD4+ | rs78393153 | 0.23191 | 0.0418813 | 3.10E-08 |
| Species Megamonas funiformis | HVEM on EM CD4+ | rs79498746 | 0.121598 | 0.0257342 | 2.30E-06 |
| Species Megamonas funiformis | HVEM on EM CD4+ | rs79605300 | 0.176044 | 0.0397788 | 9.60E-06 |
| Species Megamonas funiformis | HVEM on EM CD4+ | rs79747352 | 0.181793 | 0.0366964 | 7.30E-07 |
| Species Megamonas funiformis | HVEM on EM CD4+ | rs874689 | 0.0534554 | 0.0116456 | 4.40E-06 |
| Species Megamonas funiformis | HVEM on EM CD4+ | rs908047 | 0.0572192 | 0.0123284 | 3.50E-06 |
| Species Prevotella bivia | HLA DR+ CD4+ AC | rs117221088 | 0.1845 | 0.0410279 | 6.90E-06 |
| Species Prevotella bivia | HLA DR+ CD4+ AC | rs12070072 | 0.0568365 | 0.0119981 | 2.20E-06 |
| Species Prevotella bivia | HLA DR+ CD4+ AC | rs12200654 | 0.115444 | 0.0249668 | 3.80E-06 |
| Species Prevotella bivia | HLA DR+ CD4+ AC | rs13037592 | 0.222829 | 0.0501418 | 8.80E-06 |
| Species Prevotella bivia | HLA DR+ CD4+ AC | rs149009235 | 0.224698 | 0.0446353 | 4.80E-07 |
| Species Prevotella bivia | HLA DR+ CD4+ AC | rs2166098 | 0.141595 | 0.0313895 | 6.50E-06 |
| Species Prevotella bivia | HLA DR+ CD4+ AC | rs4610049 | 0.0657885 | 0.0138263 | 2.00E-06 |
| Species Prevotella bivia | HLA DR+ CD4+ AC | rs471538 | -0.0692225 | 0.0124687 | 2.80E-08 |
| Species Prevotella bivia | HLA DR+ CD4+ AC | rs6565531 | 0.0526467 | 0.0119033 | 9.70E-06 |
| Species Prevotella bivia | HLA DR+ CD4+ AC | rs675632 | 0.158716 | 0.0334912 | 2.10E-06 |
| Species Prevotella bivia | HLA DR+ CD4+ AC | rs77898767 | 0.187434 | 0.0415782 | 6.50E-06 |
| Species Prevotella bivia | HLA DR+ CD4+ AC | rs78007943 | -0.122704 | 0.0276713 | 9.20E-06 |
| Species Prevotella bivia | HLA DR+ CD4+ AC | rs7821969 | -0.0664149 | 0.0134515 | 7.90E-07 |
| Species Prevotella bivia | HLA DR+ CD4+ AC | rs78254123 | 0.226874 | 0.0506372 | 7.50E-06 |
| Species Prevotellamassilia | HLA DR on CD33br HLA DR+ CD14- | rs117293965 | -0.206411 | 0.042606 | 1.30E-06 |
| Species Prevotellamassilia | HLA DR on CD33br HLA DR+ CD14- | rs117476696 | 0.277274 | 0.0551208 | 4.90E-07 |
| Species Prevotellamassilia | HLA DR on CD33br HLA DR+ CD14- | rs117555310 | 0.194175 | 0.0407989 | 1.90E-06 |
| Species Prevotellamassilia | HLA DR on CD33br HLA DR+ CD14- | rs11812171 | 0.262774 | 0.0537724 | 1.00E-06 |
| Species Prevotellamassilia | HLA DR on CD33br HLA DR+ CD14- | rs139488692 | 0.336464 | 0.074174 | 5.70E-06 |
| Species Prevotellamassilia | HLA DR on CD33br HLA DR+ CD14- | rs142992142 | 0.341165 | 0.0706808 | 1.40E-06 |
| Species Prevotellamassilia | HLA DR on CD33br HLA DR+ CD14- | rs202091594 | 0.170298 | 0.0341205 | 6.00E-07 |
| Species Prevotellamassilia | HLA DR on CD33br HLA DR+ CD14- | rs2140551 | -0.10983 | 0.0181879 | 1.60E-09 |
| Species Prevotellamassilia | HLA DR on CD33br HLA DR+ CD14- | rs324324 | 0.101936 | 0.0219302 | 3.30E-06 |
| Species Prevotellamassilia | HLA DR on CD33br HLA DR+ CD14- | rs62148957 | 0.257941 | 0.0559507 | 4.00E-06 |
| Species Prevotellamassilia | HLA DR on CD33br HLA DR+ CD14- | rs6699072 | 0.17703 | 0.0386407 | 4.60E-06 |
| Species Prevotellamassilia | HLA DR on CD33br HLA DR+ CD14- | rs6825590 | 0.0801551 | 0.0179531 | 8.00E-06 |
| Species Prevotellamassilia | HLA DR on CD33br HLA DR+ CD14- | rs9970188 | -0.108387 | 0.0232379 | 3.10E-06 |

| **Table S3.** MR analysis And Reverse MR analysis of Gut microbiota and Breast cancer | |  |  |  |  |  |  |  |  |  | |  |  |  |  |  |  |  |  |  |  |  |  |  |  |  |  |  |  |  |  |  |  |  |  |  |  |  |  |  |  |  |  |  |  |  |  |  |  |  |  |  |  |  |  |  |  |  |  |  |  |  |
| --- | --- | --- | --- | --- | --- | --- | --- | --- | --- | --- | --- | --- | --- | --- | --- | --- | --- | --- | --- | --- | --- | --- | --- | --- | --- | --- | --- | --- | --- | --- | --- | --- | --- | --- | --- | --- | --- | --- | --- | --- | --- | --- | --- | --- | --- | --- | --- | --- | --- | --- | --- | --- | --- | --- | --- | --- | --- | --- | --- | --- | --- | --- |
|  |  |  |  |  |  |  |  |  |  |  |  |  |  |  |  |  |  |  |  |  |  |  |  |  |  |  |  |  |  |  |  |  |  |  |  |  |  |  |  |  |  |  |  |  |  |  |  |  |  |  |  |  |  |  |  |  |  |  |  |  |  |  |
| **Causal estimations of Gut microbiota on Breast cancer identified in MR methods** | | | | | | | | |  |  |  |  |  |  |  |  |  |  |  |  |  |  |  |  |  |  |  |  |  |  |  |  |  |  |  |  |  |  |  |  |  |  |  |  |  |  |  |  |  |  |  |  |  |  |  |  |  |  |  |  |  |  |
| Outcome | Exposure | Method | NO.of SNPs | Beta | Se | Pvalue | or | or_lci95 | or_uci95 | revPvalue |  |  |  |  |  |  |  |  |  |  |  |  |  |  |  |  |  |  |  |  |  |  |  |  |  |  |  |  |  |  |  |  |  |  |  |  |  |  |  |  |  |  |  |  |  |  |  |  |  |  |  |  |
| Breast Cancer | Phylum Actinobacteriota | MR Egger | 13 | -0.048459273 | 0.369934286 | 0.898144879 | 0.952696139 | 0.461381295 | 1.967201407 | 0.68965482 |  |  |  |  |  |  |  |  |  |  |  |  |  |  |  |  |  |  |  |  |  |  |  |  |  |  |  |  |  |  |  |  |  |  |  |  |  |  |  |  |  |  |  |  |  |  |  |  |  |  |  |  |
| Breast Cancer | Phylum Actinobacteriota | Weighted median |  | -0.338006685 | 0.148116231 | 0.02248717 | 0.71319052 | 0.533490242 | 0.953420846 | 0.40682719 |  |  |  |  |  |  |  |  |  |  |  |  |  |  |  |  |  |  |  |  |  |  |  |  |  |  |  |  |  |  |  |  |  |  |  |  |  |  |  |  |  |  |  |  |  |  |  |  |  |  |  |  |
| Breast Cancer | Phylum Actinobacteriota | Inverse variance weighted |  | -0.271307775 | 0.108487468 | 0.012390567 | 0.762381818 | 0.616348202 | 0.94301571 | 0.54899765 |  |  |  |  |  |  |  |  |  |  |  |  |  |  |  |  |  |  |  |  |  |  |  |  |  |  |  |  |  |  |  |  |  |  |  |  |  |  |  |  |  |  |  |  |  |  |  |  |  |  |  |  |
| Breast Cancer | Phylum Actinobacteriota | Simple mode |  | -0.475949118 | 0.263882177 | 0.096432379 | 0.621295094 | 0.370405034 | 1.042122968 | 0.82715443 |  |  |  |  |  |  |  |  |  |  |  |  |  |  |  |  |  |  |  |  |  |  |  |  |  |  |  |  |  |  |  |  |  |  |  |  |  |  |  |  |  |  |  |  |  |  |  |  |  |  |  |  |
| Breast Cancer | Phylum Actinobacteriota | Weighted mode |  | -0.444857963 | 0.219475969 | 0.065483787 | 0.640915303 | 0.416849154 | 0.985422236 | 0.3833839 |  |  |  |  |  |  |  |  |  |  |  |  |  |  |  |  |  |  |  |  |  |  |  |  |  |  |  |  |  |  |  |  |  |  |  |  |  |  |  |  |  |  |  |  |  |  |  |  |  |  |  |  |
| Breast Cancer | Genus An7 | MR Egger | 15 | 0.353003445 | 0.182887945 | 0.075695644 | 1.423336047 | 0.994557935 | 2.036970829 | 0.2760173 |  |  |  |  |  |  |  |  |  |  |  |  |  |  |  |  |  |  |  |  |  |  |  |  |  |  |  |  |  |  |  |  |  |  |  |  |  |  |  |  |  |  |  |  |  |  |  |  |  |  |  |  |
| Breast Cancer | Genus An7 | Weighted median |  | 0.233187929 | 0.101464104 | 0.021548657 | 1.26261874 | 1.034913952 | 1.540423799 | 0.0566375 |  |  |  |  |  |  |  |  |  |  |  |  |  |  |  |  |  |  |  |  |  |  |  |  |  |  |  |  |  |  |  |  |  |  |  |  |  |  |  |  |  |  |  |  |  |  |  |  |  |  |  |  |
| Breast Cancer | Genus An7 | Inverse variance weighted |  | 0.165804685 | 0.080848916 | 0.040286755 | 1.18034254 | 1.00736782 | 1.38301868 | 0.16478326 |  |  |  |  |  |  |  |  |  |  |  |  |  |  |  |  |  |  |  |  |  |  |  |  |  |  |  |  |  |  |  |  |  |  |  |  |  |  |  |  |  |  |  |  |  |  |  |  |  |  |  |  |
| Breast Cancer | Genus An7 | Simple mode |  | 0.223844683 | 0.163617526 | 0.192839713 | 1.250876722 | 0.907696082 | 1.723806683 | 0.14981332 |  |  |  |  |  |  |  |  |  |  |  |  |  |  |  |  |  |  |  |  |  |  |  |  |  |  |  |  |  |  |  |  |  |  |  |  |  |  |  |  |  |  |  |  |  |  |  |  |  |  |  |  |
| Breast Cancer | Genus An7 | Weighted mode |  | 0.261906901 | 0.133674252 | 0.070300355 | 1.299405563 | 0.999905372 | 1.688614609 | 0.05746472 |  |  |  |  |  |  |  |  |  |  |  |  |  |  |  |  |  |  |  |  |  |  |  |  |  |  |  |  |  |  |  |  |  |  |  |  |  |  |  |  |  |  |  |  |  |  |  |  |  |  |  |  |
| Breast Cancer | Species Bacteroides A plebeius A | MR Egger | 21 | -0.150220136 | 0.056981568 | 0.016272154 | 0.860518524 | 0.769584892 | 0.962196813 | 0.69825979 |  |  |  |  |  |  |  |  |  |  |  |  |  |  |  |  |  |  |  |  |  |  |  |  |  |  |  |  |  |  |  |  |  |  |  |  |  |  |  |  |  |  |  |  |  |  |  |  |  |  |  |  |
| Breast Cancer | Species Bacteroides A plebeius A | Weighted median |  | -0.071172608 | 0.034440567 | 0.038778072 | 0.931301129 | 0.870509924 | 0.996337626 | 0.9505165 |  |  |  |  |  |  |  |  |  |  |  |  |  |  |  |  |  |  |  |  |  |  |  |  |  |  |  |  |  |  |  |  |  |  |  |  |  |  |  |  |  |  |  |  |  |  |  |  |  |  |  |  |
| Breast Cancer | Species Bacteroides A plebeius A | Inverse variance weighted |  | -0.062130192 | 0.023873162 | 0.009254357 | 0.93976053 | 0.896800733 | 0.984778246 | 0.90322747 |  |  |  |  |  |  |  |  |  |  |  |  |  |  |  |  |  |  |  |  |  |  |  |  |  |  |  |  |  |  |  |  |  |  |  |  |  |  |  |  |  |  |  |  |  |  |  |  |  |  |  |  |
| Breast Cancer | Species Bacteroides A plebeius A | Simple mode |  | -0.080897697 | 0.058316925 | 0.180644378 | 0.922288039 | 0.822671038 | 1.033967636 | 0.83037928 |  |  |  |  |  |  |  |  |  |  |  |  |  |  |  |  |  |  |  |  |  |  |  |  |  |  |  |  |  |  |  |  |  |  |  |  |  |  |  |  |  |  |  |  |  |  |  |  |  |  |  |  |
| Breast Cancer | Species Bacteroides A plebeius A | Weighted mode |  | -0.080897697 | 0.048704338 | 0.112311144 | 0.922288039 | 0.838317644 | 1.01466936 | 0.97015675 |  |  |  |  |  |  |  |  |  |  |  |  |  |  |  |  |  |  |  |  |  |  |  |  |  |  |  |  |  |  |  |  |  |  |  |  |  |  |  |  |  |  |  |  |  |  |  |  |  |  |  |  |
| Breast Cancer | Species Bifidobacterium adolescentis | MR Egger | 22 | -0.00280778 | 0.058152972 | 0.961969887 | 0.997196158 | 0.889774157 | 1.117587166 | 0.92920888 |  |  |  |  |  |  |  |  |  |  |  |  |  |  |  |  |  |  |  |  |  |  |  |  |  |  |  |  |  |  |  |  |  |  |  |  |  |  |  |  |  |  |  |  |  |  |  |  |  |  |  |  |
| Breast Cancer | Species Bifidobacterium adolescentis | Weighted median |  | -0.037432548 | 0.031102327 | 0.228771724 | 0.963259389 | 0.906292563 | 1.023806979 | 0.45861125 |  |  |  |  |  |  |  |  |  |  |  |  |  |  |  |  |  |  |  |  |  |  |  |  |  |  |  |  |  |  |  |  |  |  |  |  |  |  |  |  |  |  |  |  |  |  |  |  |  |  |  |  |
| Breast Cancer | Species Bifidobacterium adolescentis | Inverse variance weighted |  | -0.056340674 | 0.021230037 | 0.00795865 | 0.94521707 | 0.906692836 | 0.98537815 | 0.11286605 |  |  |  |  |  |  |  |  |  |  |  |  |  |  |  |  |  |  |  |  |  |  |  |  |  |  |  |  |  |  |  |  |  |  |  |  |  |  |  |  |  |  |  |  |  |  |  |  |  |  |  |  |
| Breast Cancer | Species Bifidobacterium adolescentis | Simple mode |  | -0.02994475 | 0.06250823 | 0.6368516 | 0.970499152 | 0.85859248 | 1.096991443 | 0.81086802 |  |  |  |  |  |  |  |  |  |  |  |  |  |  |  |  |  |  |  |  |  |  |  |  |  |  |  |  |  |  |  |  |  |  |  |  |  |  |  |  |  |  |  |  |  |  |  |  |  |  |  |  |
| Breast Cancer | Species Bifidobacterium adolescentis | Weighted mode |  | -0.097619243 | 0.066373885 | 0.156185394 | 0.906994183 | 0.796353531 | 1.033006593 | 0.57080751 |  |  |  |  |  |  |  |  |  |  |  |  |  |  |  |  |  |  |  |  |  |  |  |  |  |  |  |  |  |  |  |  |  |  |  |  |  |  |  |  |  |  |  |  |  |  |  |  |  |  |  |  |
| Breast Cancer | Species Blautia sp001304935 | MR Egger | 16 | 0.239330186 | 0.129482945 | 0.085782301 | 1.270397936 | 0.985647606 | 1.637411693 | 0.23572585 |  |  |  |  |  |  |  |  |  |  |  |  |  |  |  |  |  |  |  |  |  |  |  |  |  |  |  |  |  |  |  |  |  |  |  |  |  |  |  |  |  |  |  |  |  |  |  |  |  |  |  |  |
| Breast Cancer | Species Blautia sp001304935 | Weighted median |  | 0.129943219 | 0.078000879 | 0.095729364 | 1.138763722 | 0.977322583 | 1.326872862 | 0.49201024 |  |  |  |  |  |  |  |  |  |  |  |  |  |  |  |  |  |  |  |  |  |  |  |  |  |  |  |  |  |  |  |  |  |  |  |  |  |  |  |  |  |  |  |  |  |  |  |  |  |  |  |  |
| Breast Cancer | Species Blautia sp001304935 | Inverse variance weighted |  | 0.114243894 | 0.057495121 | 0.046920297 | 1.121025503 | 1.001554665 | 1.254747467 | 0.11009661 |  |  |  |  |  |  |  |  |  |  |  |  |  |  |  |  |  |  |  |  |  |  |  |  |  |  |  |  |  |  |  |  |  |  |  |  |  |  |  |  |  |  |  |  |  |  |  |  |  |  |  |  |
| Breast Cancer | Species Blautia sp001304935 | Simple mode |  | 0.175240569 | 0.129578041 | 0.196287034 | 1.191532828 | 0.92428724 | 1.53604899 | 0.96479703 |  |  |  |  |  |  |  |  |  |  |  |  |  |  |  |  |  |  |  |  |  |  |  |  |  |  |  |  |  |  |  |  |  |  |  |  |  |  |  |  |  |  |  |  |  |  |  |  |  |  |  |  |
| Breast Cancer | Species Blautia sp001304935 | Weighted mode |  | 0.137591918 | 0.11530592 | 0.251293575 | 1.147507178 | 0.91538761 | 1.438486504 | 0.83519088 |  |  |  |  |  |  |  |  |  |  |  |  |  |  |  |  |  |  |  |  |  |  |  |  |  |  |  |  |  |  |  |  |  |  |  |  |  |  |  |  |  |  |  |  |  |  |  |  |  |  |  |  |
| Breast Cancer | Species CAG-180 sp000432435 | MR Egger | 17 | 0.19774506 | 0.107520342 | 0.085772559 | 1.218651671 | 0.987089258 | 1.50453658 | 0.94187291 |  |  |  |  |  |  |  |  |  |  |  |  |  |  |  |  |  |  |  |  |  |  |  |  |  |  |  |  |  |  |  |  |  |  |  |  |  |  |  |  |  |  |  |  |  |  |  |  |  |  |  |  |
| Breast Cancer | Species CAG-180 sp000432435 | Weighted median |  | 0.048613959 | 0.032283927 | 0.132111984 | 1.049815001 | 0.985444434 | 1.118390341 | 0.6724934 |  |  |  |  |  |  |  |  |  |  |  |  |  |  |  |  |  |  |  |  |  |  |  |  |  |  |  |  |  |  |  |  |  |  |  |  |  |  |  |  |  |  |  |  |  |  |  |  |  |  |  |  |
| Breast Cancer | Species CAG-180 sp000432435 | Inverse variance weighted |  | 0.060840847 | 0.0233112 | 0.009055794 | 1.062729764 | 1.015266252 | 1.112412187 | 0.30556179 |  |  |  |  |  |  |  |  |  |  |  |  |  |  |  |  |  |  |  |  |  |  |  |  |  |  |  |  |  |  |  |  |  |  |  |  |  |  |  |  |  |  |  |  |  |  |  |  |  |  |  |  |
| Breast Cancer | Species CAG-180 sp000432435 | Simple mode |  | 0.061017546 | 0.055603293 | 0.288719667 | 1.062917564 | 0.953167234 | 1.185304853 | 0.56110808 |  |  |  |  |  |  |  |  |  |  |  |  |  |  |  |  |  |  |  |  |  |  |  |  |  |  |  |  |  |  |  |  |  |  |  |  |  |  |  |  |  |  |  |  |  |  |  |  |  |  |  |  |
| Breast Cancer | Species CAG-180 sp000432435 | Weighted mode |  | 0.038167211 | 0.054690667 | 0.495273609 | 1.038904934 | 0.93330195 | 1.156456881 | 0.90422916 |  |  |  |  |  |  |  |  |  |  |  |  |  |  |  |  |  |  |  |  |  |  |  |  |  |  |  |  |  |  |  |  |  |  |  |  |  |  |  |  |  |  |  |  |  |  |  |  |  |  |  |  |
| Breast Cancer | Species CAG-841 sp002479075 | MR Egger | 14 | -0.249279854 | 0.159634897 | 0.144364725 | 0.779361835 | 0.56997416 | 1.065670889 | 0.24032071 |  |  |  |  |  |  |  |  |  |  |  |  |  |  |  |  |  |  |  |  |  |  |  |  |  |  |  |  |  |  |  |  |  |  |  |  |  |  |  |  |  |  |  |  |  |  |  |  |  |  |  |  |
| Breast Cancer | Species CAG-841 sp002479075 | Weighted median |  | -0.237585535 | 0.106404649 | 0.025558892 | 0.788529441 | 0.640095018 | 0.97138497 | 0.54934357 |  |  |  |  |  |  |  |  |  |  |  |  |  |  |  |  |  |  |  |  |  |  |  |  |  |  |  |  |  |  |  |  |  |  |  |  |  |  |  |  |  |  |  |  |  |  |  |  |  |  |  |  |
| Breast Cancer | Species CAG-841 sp002479075 | Inverse variance weighted |  | -0.174232223 | 0.075587327 | 0.021164029 | 0.840101784 | 0.724420405 | 0.974256111 | 0.93722501 |  |  |  |  |  |  |  |  |  |  |  |  |  |  |  |  |  |  |  |  |  |  |  |  |  |  |  |  |  |  |  |  |  |  |  |  |  |  |  |  |  |  |  |  |  |  |  |  |  |  |  |  |
| Breast Cancer | Species CAG-841 sp002479075 | Simple mode |  | -0.253763158 | 0.187694194 | 0.19942844 | 0.77587554 | 0.537060903 | 1.120883777 | 0.92745819 |  |  |  |  |  |  |  |  |  |  |  |  |  |  |  |  |  |  |  |  |  |  |  |  |  |  |  |  |  |  |  |  |  |  |  |  |  |  |  |  |  |  |  |  |  |  |  |  |  |  |  |  |
| Breast Cancer | Species CAG-841 sp002479075 | Weighted mode |  | -0.256742638 | 0.160987025 | 0.134769353 | 0.773567275 | 0.564239086 | 1.060554548 | 0.88150776 |  |  |  |  |  |  |  |  |  |  |  |  |  |  |  |  |  |  |  |  |  |  |  |  |  |  |  |  |  |  |  |  |  |  |  |  |  |  |  |  |  |  |  |  |  |  |  |  |  |  |  |  |
| Breast Cancer | Species CHKCI006 sp900018345 | MR Egger | 21 | 0.145674403 | 0.11220319 | 0.209720072 | 1.15681947 | 0.928445265 | 1.44136799 | 0.29546462 |  |  |  |  |  |  |  |  |  |  |  |  |  |  |  |  |  |  |  |  |  |  |  |  |  |  |  |  |  |  |  |  |  |  |  |  |  |  |  |  |  |  |  |  |  |  |  |  |  |  |  |  |
| Breast Cancer | Species CHKCI006 sp900018345 | Weighted median |  | 0.107864574 | 0.069077578 | 0.118406044 | 1.113896884 | 0.972847949 | 1.275395883 | 0.81468938 |  |  |  |  |  |  |  |  |  |  |  |  |  |  |  |  |  |  |  |  |  |  |  |  |  |  |  |  |  |  |  |  |  |  |  |  |  |  |  |  |  |  |  |  |  |  |  |  |  |  |  |  |
| Breast Cancer | Species CHKCI006 sp900018345 | Inverse variance weighted |  | 0.095569462 | 0.047899939 | 0.046022199 | 1.100285248 | 1.001687004 | 1.208588733 | 0.81409514 |  |  |  |  |  |  |  |  |  |  |  |  |  |  |  |  |  |  |  |  |  |  |  |  |  |  |  |  |  |  |  |  |  |  |  |  |  |  |  |  |  |  |  |  |  |  |  |  |  |  |  |  |
| Breast Cancer | Species CHKCI006 sp900018345 | Simple mode |  | 0.10550199 | 0.136231672 | 0.447735249 | 1.111268316 | 0.850856247 | 1.4513818 | 0.89427499 |  |  |  |  |  |  |  |  |  |  |  |  |  |  |  |  |  |  |  |  |  |  |  |  |  |  |  |  |  |  |  |  |  |  |  |  |  |  |  |  |  |  |  |  |  |  |  |  |  |  |  |  |
| Breast Cancer | Species CHKCI006 sp900018345 | Weighted mode |  | 0.121967052 | 0.130715855 | 0.361912459 | 1.129716879 | 0.874383665 | 1.459611241 | 0.82831002 |  |  |  |  |  |  |  |  |  |  |  |  |  |  |  |  |  |  |  |  |  |  |  |  |  |  |  |  |  |  |  |  |  |  |  |  |  |  |  |  |  |  |  |  |  |  |  |  |  |  |  |  |
| Breast Cancer | Family Fibrobacteraceae | MR Egger | 16 | -1.151985298 | 0.430865761 | 0.018169695 | 0.316008775 | 0.135812205 | 0.735291393 | 0.44013985 |  |  |  |  |  |  |  |  |  |  |  |  |  |  |  |  |  |  |  |  |  |  |  |  |  |  |  |  |  |  |  |  |  |  |  |  |  |  |  |  |  |  |  |  |  |  |  |  |  |  |  |  |
| Breast Cancer | Family Fibrobacteraceae | Weighted median |  | -0.308541109 | 0.263026149 | 0.240778371 | 0.734517756 | 0.438641705 | 1.229970449 | 0.79073777 |  |  |  |  |  |  |  |  |  |  |  |  |  |  |  |  |  |  |  |  |  |  |  |  |  |  |  |  |  |  |  |  |  |  |  |  |  |  |  |  |  |  |  |  |  |  |  |  |  |  |  |  |
| Breast Cancer | Family Fibrobacteraceae | Inverse variance weighted |  | -0.406922689 | 0.192498081 | 0.034523367 | 0.665695654 | 0.456476103 | 0.970808111 | 0.46071457 |  |  |  |  |  |  |  |  |  |  |  |  |  |  |  |  |  |  |  |  |  |  |  |  |  |  |  |  |  |  |  |  |  |  |  |  |  |  |  |  |  |  |  |  |  |  |  |  |  |  |  |  |
| Breast Cancer | Family Fibrobacteraceae | Simple mode |  | -0.182762676 | 0.44073984 | 0.6842514 | 0.832965815 | 0.351125081 | 1.976025316 | 0.22752966 |  |  |  |  |  |  |  |  |  |  |  |  |  |  |  |  |  |  |  |  |  |  |  |  |  |  |  |  |  |  |  |  |  |  |  |  |  |  |  |  |  |  |  |  |  |  |  |  |  |  |  |  |
| Breast Cancer | Family Fibrobacteraceae | Weighted mode |  | -0.199500722 | 0.437418 | 0.654860997 | 0.819139629 | 0.347552342 | 1.930614909 | 0.61553484 |  |  |  |  |  |  |  |  |  |  |  |  |  |  |  |  |  |  |  |  |  |  |  |  |  |  |  |  |  |  |  |  |  |  |  |  |  |  |  |  |  |  |  |  |  |  |  |  |  |  |  |  |
| Breast Cancer | Order Fibrobacterales | MR Egger | 15 | -1.232067565 | 0.451254515 | 0.017171421 | 0.291688868 | 0.120449293 | 0.706375216 | 0.46073106 |  |  |  |  |  |  |  |  |  |  |  |  |  |  |  |  |  |  |  |  |  |  |  |  |  |  |  |  |  |  |  |  |  |  |  |  |  |  |  |  |  |  |  |  |  |  |  |  |  |  |  |  |
| Breast Cancer | Order Fibrobacterales | Weighted median |  | -0.414812909 | 0.279204393 | 0.137359493 | 0.660463836 | 0.382107415 | 1.141596477 | 0.90944248 |  |  |  |  |  |  |  |  |  |  |  |  |  |  |  |  |  |  |  |  |  |  |  |  |  |  |  |  |  |  |  |  |  |  |  |  |  |  |  |  |  |  |  |  |  |  |  |  |  |  |  |  |
| Breast Cancer | Order Fibrobacterales | Inverse variance weighted |  | -0.588309683 | 0.205706936 | 0.004237199 | 0.555265066 | 0.371021496 | 0.83100116 | 0.48131855 |  |  |  |  |  |  |  |  |  |  |  |  |  |  |  |  |  |  |  |  |  |  |  |  |  |  |  |  |  |  |  |  |  |  |  |  |  |  |  |  |  |  |  |  |  |  |  |  |  |  |  |  |
| Breast Cancer | Order Fibrobacterales | Simple mode |  | -0.297427777 | 0.517868338 | 0.574856706 | 0.742726223 | 0.269159882 | 2.049496524 | 0.34535656 |  |  |  |  |  |  |  |  |  |  |  |  |  |  |  |  |  |  |  |  |  |  |  |  |  |  |  |  |  |  |  |  |  |  |  |  |  |  |  |  |  |  |  |  |  |  |  |  |  |  |  |  |
| Breast Cancer | Order Fibrobacterales | Weighted mode |  | -0.314856556 | 0.476863937 | 0.519800086 | 0.729893566 | 0.286645255 | 1.858550274 | 0.87376112 |  |  |  |  |  |  |  |  |  |  |  |  |  |  |  |  |  |  |  |  |  |  |  |  |  |  |  |  |  |  |  |  |  |  |  |  |  |  |  |  |  |  |  |  |  |  |  |  |  |  |  |  |
| Breast Cancer | Class Fibrobacteria | MR Egger | 20 | -0.856074636 | 0.401853641 | 0.047194718 | 0.424826412 | 0.19326214 | 0.933848089 | 0.55661207 |  |  |  |  |  |  |  |  |  |  |  |  |  |  |  |  |  |  |  |  |  |  |  |  |  |  |  |  |  |  |  |  |  |  |  |  |  |  |  |  |  |  |  |  |  |  |  |  |  |  |  |  |
| Breast Cancer | Class Fibrobacteria | Weighted median |  | -0.322901424 | 0.263882518 | 0.221082029 | 0.724045225 | 0.431662538 | 1.214470661 | 0.62862298 |  |  |  |  |  |  |  |  |  |  |  |  |  |  |  |  |  |  |  |  |  |  |  |  |  |  |  |  |  |  |  |  |  |  |  |  |  |  |  |  |  |  |  |  |  |  |  |  |  |  |  |  |
| Breast Cancer | Class Fibrobacteria | Inverse variance weighted |  | -0.397424973 | 0.186453412 | 0.033048182 | 0.672048362 | 0.466324441 | 0.96852955 | 0.42768464 |  |  |  |  |  |  |  |  |  |  |  |  |  |  |  |  |  |  |  |  |  |  |  |  |  |  |  |  |  |  |  |  |  |  |  |  |  |  |  |  |  |  |  |  |  |  |  |  |  |  |  |  |
| Breast Cancer | Class Fibrobacteria | Simple mode |  | -0.310337574 | 0.481233278 | 0.526715802 | 0.733199405 | 0.285488138 | 1.883025235 | 0.16147245 |  |  |  |  |  |  |  |  |  |  |  |  |  |  |  |  |  |  |  |  |  |  |  |  |  |  |  |  |  |  |  |  |  |  |  |  |  |  |  |  |  |  |  |  |  |  |  |  |  |  |  |  |
| Breast Cancer | Class Fibrobacteria | Weighted mode |  | -0.310337574 | 0.463873394 | 0.511534678 | 0.733199405 | 0.295369127 | 1.820032355 | 0.96366677 |  |  |  |  |  |  |  |  |  |  |  |  |  |  |  |  |  |  |  |  |  |  |  |  |  |  |  |  |  |  |  |  |  |  |  |  |  |  |  |  |  |  |  |  |  |  |  |  |  |  |  |  |
| Breast Cancer | Species Megamonas funiformis | MR Egger | 39 | 0.109227257 | 0.056516774 | 0.060961196 | 1.115415807 | 0.998455573 | 1.246076898 | 0.81304675 |  |  |  |  |  |  |  |  |  |  |  |  |  |  |  |  |  |  |  |  |  |  |  |  |  |  |  |  |  |  |  |  |  |  |  |  |  |  |  |  |  |  |  |  |  |  |  |  |  |  |  |  |
| Breast Cancer | Species Megamonas funiformis | Weighted median |  | 0.063619597 | 0.043036167 | 0.139332279 | 1.065686931 | 0.979482125 | 1.159478674 | 0.64367779 |  |  |  |  |  |  |  |  |  |  |  |  |  |  |  |  |  |  |  |  |  |  |  |  |  |  |  |  |  |  |  |  |  |  |  |  |  |  |  |  |  |  |  |  |  |  |  |  |  |  |  |  |
| Breast Cancer | Species Megamonas funiformis | Inverse variance weighted |  | 0.062925844 | 0.030721253 | 0.040532202 | 1.064947864 | 1.002715869 | 1.13104219 | 0.22563758 |  |  |  |  |  |  |  |  |  |  |  |  |  |  |  |  |  |  |  |  |  |  |  |  |  |  |  |  |  |  |  |  |  |  |  |  |  |  |  |  |  |  |  |  |  |  |  |  |  |  |  |  |
| Breast Cancer | Species Megamonas funiformis | Simple mode |  | 0.061985326 | 0.082350371 | 0.456266593 | 1.063946732 | 0.905361108 | 1.250310664 | 0.96369452 |  |  |  |  |  |  |  |  |  |  |  |  |  |  |  |  |  |  |  |  |  |  |  |  |  |  |  |  |  |  |  |  |  |  |  |  |  |  |  |  |  |  |  |  |  |  |  |  |  |  |  |  |
| Breast Cancer | Species Megamonas funiformis | Weighted mode |  | 0.06589082 | 0.074590062 | 0.382589703 | 1.068110094 | 0.922834191 | 1.236255856 | 0.53308868 |  |  |  |  |  |  |  |  |  |  |  |  |  |  |  |  |  |  |  |  |  |  |  |  |  |  |  |  |  |  |  |  |  |  |  |  |  |  |  |  |  |  |  |  |  |  |  |  |  |  |  |  |
| Breast Cancer | Species Prevotella bivia | MR Egger | 14 | 0.022766218 | 0.093695039 | 0.812122931 | 1.023027346 | 0.851397588 | 1.229255246 | 0.69871046 |  |  |  |  |  |  |  |  |  |  |  |  |  |  |  |  |  |  |  |  |  |  |  |  |  |  |  |  |  |  |  |  |  |  |  |  |  |  |  |  |  |  |  |  |  |  |  |  |  |  |  |  |
| Breast Cancer | Species Prevotella bivia | Weighted median |  | 0.063511028 | 0.062469518 | 0.309309352 | 1.065571236 | 0.94277349 | 1.204363585 | 0.95833689 |  |  |  |  |  |  |  |  |  |  |  |  |  |  |  |  |  |  |  |  |  |  |  |  |  |  |  |  |  |  |  |  |  |  |  |  |  |  |  |  |  |  |  |  |  |  |  |  |  |  |  |  |
| Breast Cancer | Species Prevotella bivia | Inverse variance weighted |  | 0.128775613 | 0.04443443 | 0.003754257 | 1.13743487 | 1.042565111 | 1.240937444 | 0.77443314 |  |  |  |  |  |  |  |  |  |  |  |  |  |  |  |  |  |  |  |  |  |  |  |  |  |  |  |  |  |  |  |  |  |  |  |  |  |  |  |  |  |  |  |  |  |  |  |  |  |  |  |  |
| Breast Cancer | Species Prevotella bivia | Simple mode |  | 0.031811286 | 0.118324604 | 0.792266365 | 1.032322673 | 0.818644842 | 1.301773427 | 0.54211815 |  |  |  |  |  |  |  |  |  |  |  |  |  |  |  |  |  |  |  |  |  |  |  |  |  |  |  |  |  |  |  |  |  |  |  |  |  |  |  |  |  |  |  |  |  |  |  |  |  |  |  |  |
| Breast Cancer | Species Prevotella bivia | Weighted mode |  | 0.01106745 | 0.102733036 | 0.915854636 | 1.011128921 | 0.826719929 | 1.236672371 | 0.90090444 |  |  |  |  |  |  |  |  |  |  |  |  |  |  |  |  |  |  |  |  |  |  |  |  |  |  |  |  |  |  |  |  |  |  |  |  |  |  |  |  |  |  |  |  |  |  |  |  |  |  |  |  |
| Breast Cancer | Species Prevotellamassilia | MR Egger | 14 | 0.21216969 | 0.087495405 | 0.032028153 | 1.236357665 | 1.041517408 | 1.467647363 | 0.87539442 |  |  |  |  |  |  |  |  |  |  |  |  |  |  |  |  |  |  |  |  |  |  |  |  |  |  |  |  |  |  |  |  |  |  |  |  |  |  |  |  |  |  |  |  |  |  |  |  |  |  |  |  |
| Breast Cancer | Species Prevotellamassilia | Weighted median |  | 0.069687228 | 0.047242681 | 0.140188223 | 1.072172783 | 0.97735198 | 1.176192918 | 0.74832836 |  |  |  |  |  |  |  |  |  |  |  |  |  |  |  |  |  |  |  |  |  |  |  |  |  |  |  |  |  |  |  |  |  |  |  |  |  |  |  |  |  |  |  |  |  |  |  |  |  |  |  |  |
| Breast Cancer | Species Prevotellamassilia | Inverse variance weighted |  | 0.071892381 | 0.035839387 | 0.044860503 | 1.074539697 | 1.00164854 | 1.152735231 | 0.57617527 |  |  |  |  |  |  |  |  |  |  |  |  |  |  |  |  |  |  |  |  |  |  |  |  |  |  |  |  |  |  |  |  |  |  |  |  |  |  |  |  |  |  |  |  |  |  |  |  |  |  |  |  |
| Breast Cancer | Species Prevotellamassilia | Simple mode |  | 0.056723842 | 0.074640911 | 0.460841152 | 1.058363494 | 0.914322117 | 1.225097004 | 0.14688259 |  |  |  |  |  |  |  |  |  |  |  |  |  |  |  |  |  |  |  |  |  |  |  |  |  |  |  |  |  |  |  |  |  |  |  |  |  |  |  |  |  |  |  |  |  |  |  |  |  |  |  |  |
| Breast Cancer | Species Prevotellamassilia | Weighted mode |  | 0.083307281 | 0.056988017 | 0.167534894 | 1.086875734 | 0.972009956 | 1.215315597 | 0.84341453 |  |  |  |  |  |  |  |  |  |  |  |  |  |  |  |  |  |  |  |  |  |  |  |  |  |  |  |  |  |  |  |  |  |  |  |  |  |  |  |  |  |  |  |  |  |  |  |  |  |  |  |  |
| Breast Cancer | Genus RUG147 | MR Egger | 6 | 0.790897923 | 0.528686752 | 0.208993557 | 2.205375796 | 0.782447767 | 6.215983491 | 0.87638204 |  |  |  |  |  |  |  |  |  |  |  |  |  |  |  |  |  |  |  |  |  |  |  |  |  |  |  |  |  |  |  |  |  |  |  |  |  |  |  |  |  |  |  |  |  |  |  |  |  |  |  |  |
| Breast Cancer | Genus RUG147 | Weighted median |  | 0.473911186 | 0.220440073 | 0.031567765 | 1.606264322 | 1.042736642 | 2.474340085 | 0.45792384 |  |  |  |  |  |  |  |  |  |  |  |  |  |  |  |  |  |  |  |  |  |  |  |  |  |  |  |  |  |  |  |  |  |  |  |  |  |  |  |  |  |  |  |  |  |  |  |  |  |  |  |  |
| Breast Cancer | Genus RUG147 | Inverse variance weighted |  | 0.435606966 | 0.193308983 | 0.024232472 | 1.545901084 | 1.058360866 | 2.258029598 | 0.91692949 |  |  |  |  |  |  |  |  |  |  |  |  |  |  |  |  |  |  |  |  |  |  |  |  |  |  |  |  |  |  |  |  |  |  |  |  |  |  |  |  |  |  |  |  |  |  |  |  |  |  |  |  |
| Breast Cancer | Genus RUG147 | Simple mode |  | 0.502875408 | 0.328490014 | 0.186361858 | 1.653468839 | 0.868519692 | 3.147837898 | 0.67915187 |  |  |  |  |  |  |  |  |  |  |  |  |  |  |  |  |  |  |  |  |  |  |  |  |  |  |  |  |  |  |  |  |  |  |  |  |  |  |  |  |  |  |  |  |  |  |  |  |  |  |  |  |
| Breast Cancer | Genus RUG147 | Weighted mode |  | 0.495936559 | 0.279424991 | 0.13609525 | 1.642035382 | 0.949579121 | 2.83944764 | 0.5210739 |  |  |  |  |  |  |  |  |  |  |  |  |  |  |  |  |  |  |  |  |  |  |  |  |  |  |  |  |  |  |  |  |  |  |  |  |  |  |  |  |  |  |  |  |  |  |  |  |  |  |  |  |
| Breast Cancer | Species UBA7177 sp002491225 | MR Egger | 20 | 0.099047336 | 0.2033349 | 0.63205639 | 1.104118562 | 0.741196825 | 1.644742339 | 0.86873703 |  |  |  |  |  |  |  |  |  |  |  |  |  |  |  |  |  |  |  |  |  |  |  |  |  |  |  |  |  |  |  |  |  |  |  |  |  |  |  |  |  |  |  |  |  |  |  |  |  |  |  |  |
| Breast Cancer | Species UBA7177 sp002491225 | Weighted median |  | 0.366943048 | 0.117950263 | 0.001864561 | 1.443315718 | 1.145407574 | 1.818706553 | 0.52987266 |  |  |  |  |  |  |  |  |  |  |  |  |  |  |  |  |  |  |  |  |  |  |  |  |  |  |  |  |  |  |  |  |  |  |  |  |  |  |  |  |  |  |  |  |  |  |  |  |  |  |  |  |
| Breast Cancer | Species UBA7177 sp002491225 | Inverse variance weighted |  | 0.26487761 | 0.083095141 | 0.001434377 | 1.303271459 | 1.107395801 | 1.533793513 | 0.57753737 |  |  |  |  |  |  |  |  |  |  |  |  |  |  |  |  |  |  |  |  |  |  |  |  |  |  |  |  |  |  |  |  |  |  |  |  |  |  |  |  |  |  |  |  |  |  |  |  |  |  |  |  |
| Breast Cancer | Species UBA7177 sp002491225 | Simple mode |  | 0.428449043 | 0.206963632 | 0.052307377 | 1.534875151 | 1.023062238 | 2.302735494 | 0.51880583 |  |  |  |  |  |  |  |  |  |  |  |  |  |  |  |  |  |  |  |  |  |  |  |  |  |  |  |  |  |  |  |  |  |  |  |  |  |  |  |  |  |  |  |  |  |  |  |  |  |  |  |  |
| Breast Cancer | Species UBA7177 sp002491225 | Weighted mode |  | 0.399343074 | 0.182136367 | 0.040991834 | 1.490845002 | 1.043265602 | 2.130443882 | 0.22815299 |  |  |  |  |  |  |  |  |  |  |  |  |  |  |  |  |  |  |  |  |  |  |  |  |  |  |  |  |  |  |  |  |  |  |  |  |  |  |  |  |  |  |  |  |  |  |  |  |  |  |  |  |
| Breast Cancer | Genus Veillonella | MR Egger | 8 | 0.039116702 | 0.13093472 | 0.775207918 | 1.039891834 | 0.804515256 | 1.344132406 | 0.4975281 |  |  |  |  |  |  |  |  |  |  |  |  |  |  |  |  |  |  |  |  |  |  |  |  |  |  |  |  |  |  |  |  |  |  |  |  |  |  |  |  |  |  |  |  |  |  |  |  |  |  |  |  |
| Breast Cancer | Genus Veillonella | Weighted median |  | 0.103782523 | 0.062236117 | 0.095402902 | 1.109359169 | 0.981964358 | 1.253281502 | 0.32026699 |  |  |  |  |  |  |  |  |  |  |  |  |  |  |  |  |  |  |  |  |  |  |  |  |  |  |  |  |  |  |  |  |  |  |  |  |  |  |  |  |  |  |  |  |  |  |  |  |  |  |  |  |
| Breast Cancer | Genus Veillonella | Inverse variance weighted |  | 0.103504328 | 0.048510008 | 0.03286984 | 1.109050593 | 1.008460299 | 1.219674408 | 0.21953714 |  |  |  |  |  |  |  |  |  |  |  |  |  |  |  |  |  |  |  |  |  |  |  |  |  |  |  |  |  |  |  |  |  |  |  |  |  |  |  |  |  |  |  |  |  |  |  |  |  |  |  |  |
| Breast Cancer | Genus Veillonella | Simple mode |  | 0.102937975 | 0.083740857 | 0.258693315 | 1.108422657 | 0.940640639 | 1.306131943 | 0.75731971 |  |  |  |  |  |  |  |  |  |  |  |  |  |  |  |  |  |  |  |  |  |  |  |  |  |  |  |  |  |  |  |  |  |  |  |  |  |  |  |  |  |  |  |  |  |  |  |  |  |  |  |  |
| Breast Cancer | Genus Veillonella | Weighted mode |  | 0.108766664 | 0.073960257 | 0.184863143 | 1.114902173 | 0.964451783 | 1.288822187 | 0.54595142 |  |  |  |  |  |  |  |  |  |  |  |  |  |  |  |  |  |  |  |  |  |  |  |  |  |  |  |  |  |  |  |  |  |  |  |  |  |  |  |  |  |  |  |  |  |  |  |  |  |  |  |  |
| Breast Cancer | Species Phascolarctobacterium sp003150755 | MR Egger | 18 | -0.198582328 | 0.117974608 | 0.111730992 | 0.819892267 | 0.650631038 | 1.033186693 | 0.98563595 |  |  |  |  |  |  |  |  |  |  |  |  |  |  |  |  |  |  |  |  |  |  |  |  |  |  |  |  |  |  |  |  |  |  |  |  |  |  |  |  |  |  |  |  |  |  |  |  |  |  |  |  |
| Breast Cancer | Species Phascolarctobacterium sp003150755 | Weighted median |  | -0.070093293 | 0.06719145 | 0.296861374 | 0.932306838 | 0.817267733 | 1.063538917 | 0.77817189 |  |  |  |  |  |  |  |  |  |  |  |  |  |  |  |  |  |  |  |  |  |  |  |  |  |  |  |  |  |  |  |  |  |  |  |  |  |  |  |  |  |  |  |  |  |  |  |  |  |  |  |  |
| Breast Cancer | Species Phascolarctobacterium sp003150755 | Inverse variance weighted |  | -0.134280105 | 0.054102327 | 0.013065995 | 0.874345122 | 0.786375657 | 0.972155465 | 0.33250073 |  |  |  |  |  |  |  |  |  |  |  |  |  |  |  |  |  |  |  |  |  |  |  |  |  |  |  |  |  |  |  |  |  |  |  |  |  |  |  |  |  |  |  |  |  |  |  |  |  |  |  |  |
| Breast Cancer | Species Phascolarctobacterium sp003150755 | Simple mode |  | -0.149436324 | 0.113130711 | 0.204034747 | 0.861193273 | 0.689924906 | 1.074977649 | 0.56645029 |  |  |  |  |  |  |  |  |  |  |  |  |  |  |  |  |  |  |  |  |  |  |  |  |  |  |  |  |  |  |  |  |  |  |  |  |  |  |  |  |  |  |  |  |  |  |  |  |  |  |  |  |
| Breast Cancer | Species Phascolarctobacterium sp003150755 | Weighted mode |  | -0.088639235 | 0.078486156 | 0.274430098 | 0.915175677 | 0.784685639 | 1.067365679 | 0.7009118 |  |  |  |  |  |  |  |  |  |  |  |  |  |  |  |  |  |  |  |  |  |  |  |  |  |  |  |  |  |  |  |  |  |  |  |  |  |  |  |  |  |  |  |  |  |  |  |  |  |  |  |  |
|  |  |  |  |  |  |  |  |  |  |  |  |  |  |  |  |  |  |  |  |  |  |  |  |  |  |  |  |  |  |  |  |  |  |  |  |  |  |  |  |  |  |  |  |  |  |  |  |  |  |  |  |  |  |  |  |  |  |  |  |  |  |  |
| **A Reverse MR analysis of Gut micrbiota and Breast cancer identified in MR methods** | | | | | | | | |  |  |  |  |  |  |  |  |  |  |  |  |  |  |  |  |  |  |  |  |  |  |  |  |  |  |  |  |  |  |  |  |  |  |  |  |  |  |  |  |  |  |  |  |  |  |  |  |  |  |  |  |  |  |
| Outcome | Exposure | Method | Beta | Se | Pvalue | or | or_lci95 | or_uci95 |  |  |  |  |  |  |  |  |  |  |  |  |  |  |  |  |  |  |  |  |  |  |  |  |  |  |  |  |  |  |  |  |  |  |  |  |  |  |  |  |  |  |  |  |  |  |  |  |  |  |  |  |  |  |
| Phylum Actinobacteriota | Breast Cancer | MR Egger | -0.008 | 0.0191945601110686 | 0.689654823377265 | 0.99230763665092 | 0.955669210432096 | 1.03035070608848 | |  |  |  |  |  |  |  |  |  |  |  |  |  |  |  |  |  |  |  |  |  |  |  |  |  |  |  |  |  |  |  |  |  |  |  |  |  |  |  |  |  |  |  |  |  |  |  |  |  |  |  |  |  |
| Phylum Actinobacteriota | Breast Cancer | Weighted median | -0.011 | 0.0128504477557201 | 0.406827190372058 | 0.989397291242921 | 0.964788670842899 | 1.01463359749405 | |  |  |  |  |  |  |  |  |  |  |  |  |  |  |  |  |  |  |  |  |  |  |  |  |  |  |  |  |  |  |  |  |  |  |  |  |  |  |  |  |  |  |  |  |  |  |  |  |  |  |  |  |  |
| Phylum Actinobacteriota | Breast Cancer | Inverse variance weighted | -0.005 | 0.00862821291395119 | 0.548997653550015 | 0.994842777382143 | 0.978160155375575 | 1.01180992322204 | |  |  |  |  |  |  |  |  |  |  |  |  |  |  |  |  |  |  |  |  |  |  |  |  |  |  |  |  |  |  |  |  |  |  |  |  |  |  |  |  |  |  |  |  |  |  |  |  |  |  |  |  |  |
| Phylum Actinobacteriota | Breast Cancer | Simple mode | -0.005 | 0.0227377714589132 | 0.827154427478736 | 0.995014987188852 | 0.951644715838391 | 1.04036181597268 | |  |  |  |  |  |  |  |  |  |  |  |  |  |  |  |  |  |  |  |  |  |  |  |  |  |  |  |  |  |  |  |  |  |  |  |  |  |  |  |  |  |  |  |  |  |  |  |  |  |  |  |  |  |
| Phylum Actinobacteriota | Breast Cancer | Weighted mode | -0.013 | 0.0151182403430774 | 0.383383900007092 | 0.98676363970941 | 0.957953067068945 | 1.01644069435657 | |  |  |  |  |  |  |  |  |  |  |  |  |  |  |  |  |  |  |  |  |  |  |  |  |  |  |  |  |  |  |  |  |  |  |  |  |  |  |  |  |  |  |  |  |  |  |  |  |  |  |  |  |  |
| Genus An7 | Breast Cancer | MR Egger | -0.034 | 0.0308715668518327 | 0.276017295814111 | 0.966468279445176 | 0.90972304501834 | 1.02675307643206 | |  |  |  |  |  |  |  |  |  |  |  |  |  |  |  |  |  |  |  |  |  |  |  |  |  |  |  |  |  |  |  |  |  |  |  |  |  |  |  |  |  |  |  |  |  |  |  |  |  |  |  |  |  |
| Genus An7 | Breast Cancer | Weighted median | -0.036 | 0.0190866250284314 | 0.0566375019987827 | 0.964272858347013 | 0.928866028136726 | 1.00102933811662 | |  |  |  |  |  |  |  |  |  |  |  |  |  |  |  |  |  |  |  |  |  |  |  |  |  |  |  |  |  |  |  |  |  |  |  |  |  |  |  |  |  |  |  |  |  |  |  |  |  |  |  |  |  |
| Genus An7 | Breast Cancer | Inverse variance weighted | -0.019 | 0.0137545604588854 | 0.164783255221614 | 0.981074064270799 | 0.954978681278574 | 1.00788251973978 | |  |  |  |  |  |  |  |  |  |  |  |  |  |  |  |  |  |  |  |  |  |  |  |  |  |  |  |  |  |  |  |  |  |  |  |  |  |  |  |  |  |  |  |  |  |  |  |  |  |  |  |  |  |
| Genus An7 | Breast Cancer | Simple mode | -0.05 | 0.0339615519255709 | 0.149813317788437 | 0.951353558980721 | 0.890088706233421 | 1.01683527478432 | |  |  |  |  |  |  |  |  |  |  |  |  |  |  |  |  |  |  |  |  |  |  |  |  |  |  |  |  |  |  |  |  |  |  |  |  |  |  |  |  |  |  |  |  |  |  |  |  |  |  |  |  |  |
| Genus An7 | Breast Cancer | Weighted mode | -0.039 | 0.0198835213948104 | 0.0574647220032957 | 0.96185296637326 | 0.925088950361958 | 1.00007802337176 | |  |  |  |  |  |  |  |  |  |  |  |  |  |  |  |  |  |  |  |  |  |  |  |  |  |  |  |  |  |  |  |  |  |  |  |  |  |  |  |  |  |  |  |  |  |  |  |  |  |  |  |  |  |
| Species Bacteroides A plebeius A | Breast Cancer | MR Egger | -0.031 | 0.0785440578644006 | 0.698259791805565 | 0.969790819558235 | 0.831419137442745 | 1.13119146690823 | |  |  |  |  |  |  |  |  |  |  |  |  |  |  |  |  |  |  |  |  |  |  |  |  |  |  |  |  |  |  |  |  |  |  |  |  |  |  |  |  |  |  |  |  |  |  |  |  |  |  |  |  |  |
| Species Bacteroides A plebeius A | Breast Cancer | Weighted median | 0.0028872277835011 | 0.0465245271031108 | 0.950516495058338 | 1.0028913998399 | 0.915485415769944 | 1.09864247157551 | |  |  |  |  |  |  |  |  |  |  |  |  |  |  |  |  |  |  |  |  |  |  |  |  |  |  |  |  |  |  |  |  |  |  |  |  |  |  |  |  |  |  |  |  |  |  |  |  |  |  |  |  |  |
| Species Bacteroides A plebeius A | Breast Cancer | Inverse variance weighted | -0.004 | 0.0349258857317352 | 0.903227471143231 | 0.995762529893193 | 0.929878627272218 | 1.06631444885228 | |  |  |  |  |  |  |  |  |  |  |  |  |  |  |  |  |  |  |  |  |  |  |  |  |  |  |  |  |  |  |  |  |  |  |  |  |  |  |  |  |  |  |  |  |  |  |  |  |  |  |  |  |  |
| Species Bacteroides A plebeius A | Breast Cancer | Simple mode | -0.019 | 0.0877544896256476 | 0.830379282223687 | 0.981256221464459 | 0.826198293728177 | 1.16541486404896 | |  |  |  |  |  |  |  |  |  |  |  |  |  |  |  |  |  |  |  |  |  |  |  |  |  |  |  |  |  |  |  |  |  |  |  |  |  |  |  |  |  |  |  |  |  |  |  |  |  |  |  |  |  |
| Species Bacteroides A plebeius A | Breast Cancer | Weighted mode | 0.00219918727721768 | 0.0584166885799897 | 0.970156748096841 | 1.00220160726323 | 0.893778298927464 | 1.12377763345373 | |  |  |  |  |  |  |  |  |  |  |  |  |  |  |  |  |  |  |  |  |  |  |  |  |  |  |  |  |  |  |  |  |  |  |  |  |  |  |  |  |  |  |  |  |  |  |  |  |  |  |  |  |  |
| Species Bifidobacterium adolescentis | Breast Cancer | MR Egger | 0.007451380674702 | 0.0833337980247644 | 0.929208875597342 | 1.00747921129425 | 0.855659413489398 | 1.1862364220956 | |  |  |  |  |  |  |  |  |  |  |  |  |  |  |  |  |  |  |  |  |  |  |  |  |  |  |  |  |  |  |  |  |  |  |  |  |  |  |  |  |  |  |  |  |  |  |  |  |  |  |  |  |  |
| Species Bifidobacterium adolescentis | Breast Cancer | Weighted median | -0.041 | 0.0547958459855576 | 0.45861125090442 | 0.960202432120651 | 0.862421621275932 | 1.06906956864828 | |  |  |  |  |  |  |  |  |  |  |  |  |  |  |  |  |  |  |  |  |  |  |  |  |  |  |  |  |  |  |  |  |  |  |  |  |  |  |  |  |  |  |  |  |  |  |  |  |  |  |  |  |  |
| Species Bifidobacterium adolescentis | Breast Cancer | Inverse variance weighted | -0.059 | 0.0374593380954056 | 0.112866045264461 | 0.9423395702787 | 0.875631539073161 | 1.01412960370635 | |  |  |  |  |  |  |  |  |  |  |  |  |  |  |  |  |  |  |  |  |  |  |  |  |  |  |  |  |  |  |  |  |  |  |  |  |  |  |  |  |  |  |  |  |  |  |  |  |  |  |  |  |  |
| Species Bifidobacterium adolescentis | Breast Cancer | Simple mode | -0.024 | 0.0980774732203008 | 0.810868015065238 | 0.976650669925433 | 0.805849650110008 | 1.18365321736578 | |  |  |  |  |  |  |  |  |  |  |  |  |  |  |  |  |  |  |  |  |  |  |  |  |  |  |  |  |  |  |  |  |  |  |  |  |  |  |  |  |  |  |  |  |  |  |  |  |  |  |  |  |  |
| Species Bifidobacterium adolescentis | Breast Cancer | Weighted mode | -0.037 | 0.0649593975896394 | 0.570807508058788 | 0.963551620227257 | 0.848360482613721 | 1.094383513 |  |  |  |  |  |  |  |  |  |  |  |  |  |  |  |  |  |  |  |  |  |  |  |  |  |  |  |  |  |  |  |  |  |  |  |  |  |  |  |  |  |  |  |  |  |  |  |  |  |  |  |  |  |  |
| Species Blautia sp001304935 | Breast Cancer | MR Egger | 0.0522966235034435 | 0.0434243536569288 | 0.235725847830378 | 1.05368824486203 | 0.967717464656674 | 1.14729655907814 | |  |  |  |  |  |  |  |  |  |  |  |  |  |  |  |  |  |  |  |  |  |  |  |  |  |  |  |  |  |  |  |  |  |  |  |  |  |  |  |  |  |  |  |  |  |  |  |  |  |  |  |  |  |
| Species Blautia sp001304935 | Breast Cancer | Weighted median | 0.018268335945379 | 0.0265870121663659 | 0.492010236864587 | 1.01843622277389 | 0.966724034017998 | 1.07291460991921 | |  |  |  |  |  |  |  |  |  |  |  |  |  |  |  |  |  |  |  |  |  |  |  |  |  |  |  |  |  |  |  |  |  |  |  |  |  |  |  |  |  |  |  |  |  |  |  |  |  |  |  |  |  |
| Species Blautia sp001304935 | Breast Cancer | Inverse variance weighted | 0.0309156074226185 | 0.0193493550604478 | 0.110096608355126 | 1.03139845783939 | 0.993015378155094 | 1.07126516087783 | |  |  |  |  |  |  |  |  |  |  |  |  |  |  |  |  |  |  |  |  |  |  |  |  |  |  |  |  |  |  |  |  |  |  |  |  |  |  |  |  |  |  |  |  |  |  |  |  |  |  |  |  |  |
| Species Blautia sp001304935 | Breast Cancer | Simple mode | 0.00271554372081995 | 0.0611444781751546 | 0.964797034969781 | 1.00271923414942 | 0.889471659534952 | 1.13038549542869 | |  |  |  |  |  |  |  |  |  |  |  |  |  |  |  |  |  |  |  |  |  |  |  |  |  |  |  |  |  |  |  |  |  |  |  |  |  |  |  |  |  |  |  |  |  |  |  |  |  |  |  |  |  |
| Species Blautia sp001304935 | Breast Cancer | Weighted mode | 0.008593070963821 | 0.0410346219738571 | 0.835190878059985 | 1.008630097 | 0.930684643284022 | 1.0931035347795 | |  |  |  |  |  |  |  |  |  |  |  |  |  |  |  |  |  |  |  |  |  |  |  |  |  |  |  |  |  |  |  |  |  |  |  |  |  |  |  |  |  |  |  |  |  |  |  |  |  |  |  |  |  |
| Species CAG-180 sp000432435 | Breast Cancer | MR Egger | -0.007 | 0.0952995726747343 | 0.941872909833301 | 0.993030606688985 | 0.823838341839102 | 1.19697000702801 | |  |  |  |  |  |  |  |  |  |  |  |  |  |  |  |  |  |  |  |  |  |  |  |  |  |  |  |  |  |  |  |  |  |  |  |  |  |  |  |  |  |  |  |  |  |  |  |  |  |  |  |  |  |
| Species CAG-180 sp000432435 | Breast Cancer | Weighted median | -0.027 | 0.0632763233530133 | 0.67249339856804 | 0.973605876128802 | 0.860045232116084 | 1.10216110343437 | |  |  |  |  |  |  |  |  |  |  |  |  |  |  |  |  |  |  |  |  |  |  |  |  |  |  |  |  |  |  |  |  |  |  |  |  |  |  |  |  |  |  |  |  |  |  |  |  |  |  |  |  |  |
| Species CAG-180 sp000432435 | Breast Cancer | Inverse variance weighted | -0.044 | 0.0428389308876503 | 0.305561786729399 | 0.957057431605648 | 0.879979927224215 | 1.04088616007512 | |  |  |  |  |  |  |  |  |  |  |  |  |  |  |  |  |  |  |  |  |  |  |  |  |  |  |  |  |  |  |  |  |  |  |  |  |  |  |  |  |  |  |  |  |  |  |  |  |  |  |  |  |  |
| Species CAG-180 sp000432435 | Breast Cancer | Simple mode | -0.067 | 0.114973821887123 | 0.561108078502132 | 0.934835245451074 | 0.746220859568356 | 1.17112370276419 | |  |  |  |  |  |  |  |  |  |  |  |  |  |  |  |  |  |  |  |  |  |  |  |  |  |  |  |  |  |  |  |  |  |  |  |  |  |  |  |  |  |  |  |  |  |  |  |  |  |  |  |  |  |
| Species CAG-180 sp000432435 | Breast Cancer | Weighted mode | -0.009 | 0.0705598733271112 | 0.904229161440679 | 0.991492583107188 | 0.863431111118129 | 1.13854774248697 | |  |  |  |  |  |  |  |  |  |  |  |  |  |  |  |  |  |  |  |  |  |  |  |  |  |  |  |  |  |  |  |  |  |  |  |  |  |  |  |  |  |  |  |  |  |  |  |  |  |  |  |  |  |
| Species CAG-841 sp002479075 | Breast Cancer | MR Egger | -0.036 | 0.0305005583926984 | 0.240320708747444 | 0.964285407609703 | 0.90832861355286 | 1.023689371 |  |  |  |  |  |  |  |  |  |  |  |  |  |  |  |  |  |  |  |  |  |  |  |  |  |  |  |  |  |  |  |  |  |  |  |  |  |  |  |  |  |  |  |  |  |  |  |  |  |  |  |  |  |  |
| Species CAG-841 sp002479075 | Breast Cancer | Weighted median | -0.012 | 0.0203520592668656 | 0.549343571302214 | 0.987888270088519 | 0.949256995165922 | 1.02809169608269 | |  |  |  |  |  |  |  |  |  |  |  |  |  |  |  |  |  |  |  |  |  |  |  |  |  |  |  |  |  |  |  |  |  |  |  |  |  |  |  |  |  |  |  |  |  |  |  |  |  |  |  |  |  |
| Species CAG-841 sp002479075 | Breast Cancer | Inverse variance weighted | 0.00109178090883379 | 0.0138624540102914 | 0.937225010623101 | 1.00109237711857 | 0.974258481674242 | 1.02866535562787 | |  |  |  |  |  |  |  |  |  |  |  |  |  |  |  |  |  |  |  |  |  |  |  |  |  |  |  |  |  |  |  |  |  |  |  |  |  |  |  |  |  |  |  |  |  |  |  |  |  |  |  |  |  |
| Species CAG-841 sp002479075 | Breast Cancer | Simple mode | 0.00344874105851672 | 0.0376422746735178 | 0.927458194641226 | 1.00345469480831 | 0.932086071373465 | 1.08028792131728 | |  |  |  |  |  |  |  |  |  |  |  |  |  |  |  |  |  |  |  |  |  |  |  |  |  |  |  |  |  |  |  |  |  |  |  |  |  |  |  |  |  |  |  |  |  |  |  |  |  |  |  |  |  |
| Species CAG-841 sp002479075 | Breast Cancer | Weighted mode | -0.004 | 0.0279533078327703 | 0.88150775643983 | 0.995815402987099 | 0.94272386973639 | 1.05189690073685 | |  |  |  |  |  |  |  |  |  |  |  |  |  |  |  |  |  |  |  |  |  |  |  |  |  |  |  |  |  |  |  |  |  |  |  |  |  |  |  |  |  |  |  |  |  |  |  |  |  |  |  |  |  |
| Species CHKCI006 sp900018345 | Breast Cancer | MR Egger | 0.0376108931660158 | 0.0354670122094532 | 0.295464619981065 | 1.03832713408101 | 0.968599126563887 | 1.11307475693638 | |  |  |  |  |  |  |  |  |  |  |  |  |  |  |  |  |  |  |  |  |  |  |  |  |  |  |  |  |  |  |  |  |  |  |  |  |  |  |  |  |  |  |  |  |  |  |  |  |  |  |  |  |  |
| Species CHKCI006 sp900018346 | Breast Cancer | Weighted median | 0.00584623911902874 | 0.0249433382331027 | 0.814689376968471 | 1.0058633617263 | 0.957870484466929 | 1.05626086080563 | |  |  |  |  |  |  |  |  |  |  |  |  |  |  |  |  |  |  |  |  |  |  |  |  |  |  |  |  |  |  |  |  |  |  |  |  |  |  |  |  |  |  |  |  |  |  |  |  |  |  |  |  |  |
| Species CHKCI006 sp900018347 | Breast Cancer | Inverse variance weighted | 0.00374897644479274 | 0.0159431618009299 | 0.814095138175122 | 1.00375601264709 | 0.972875051579455 | 1.03561719594872 | |  |  |  |  |  |  |  |  |  |  |  |  |  |  |  |  |  |  |  |  |  |  |  |  |  |  |  |  |  |  |  |  |  |  |  |  |  |  |  |  |  |  |  |  |  |  |  |  |  |  |  |  |  |
| Species CHKCI006 sp900018348 | Breast Cancer | Simple mode | 0.00644956507951655 | 0.0482229074856296 | 0.89427499424944 | 1.0064704083102 | 0.91569921732969 | 1.10623954201699 | |  |  |  |  |  |  |  |  |  |  |  |  |  |  |  |  |  |  |  |  |  |  |  |  |  |  |  |  |  |  |  |  |  |  |  |  |  |  |  |  |  |  |  |  |  |  |  |  |  |  |  |  |  |
| Species CHKCI006 sp900018349 | Breast Cancer | Weighted mode | 0.0077872123957623 | 0.0356730631708406 | 0.828310018779716 | 1.00781761159131 | 0.939758838501922 | 1.08080530517036 | |  |  |  |  |  |  |  |  |  |  |  |  |  |  |  |  |  |  |  |  |  |  |  |  |  |  |  |  |  |  |  |  |  |  |  |  |  |  |  |  |  |  |  |  |  |  |  |  |  |  |  |  |  |
| Family Fibrobacteraceae | Breast Cancer | MR Egger | 0.00982117702823631 | 0.0125923807805632 | 0.440139851696055 | 1.00986956306022 | 0.985249973994678 | 1.03510435048328 | |  |  |  |  |  |  |  |  |  |  |  |  |  |  |  |  |  |  |  |  |  |  |  |  |  |  |  |  |  |  |  |  |  |  |  |  |  |  |  |  |  |  |  |  |  |  |  |  |  |  |  |  |  |
| Family Fibrobacteraceae | Breast Cancer | Weighted median | 0.00196020768757412 | 0.00738717830090906 | 0.790737772395493 | 1.0019621301506 | 0.987559370704022 | 1.01657494226422 | |  |  |  |  |  |  |  |  |  |  |  |  |  |  |  |  |  |  |  |  |  |  |  |  |  |  |  |  |  |  |  |  |  |  |  |  |  |  |  |  |  |  |  |  |  |  |  |  |  |  |  |  |  |
| Family Fibrobacteraceae | Breast Cancer | Inverse variance weighted | -0.004 | 0.00569965640697754 | 0.460714573920276 | 0.995804356808404 | 0.98474180787322 | 1.00699118196296 | |  |  |  |  |  |  |  |  |  |  |  |  |  |  |  |  |  |  |  |  |  |  |  |  |  |  |  |  |  |  |  |  |  |  |  |  |  |  |  |  |  |  |  |  |  |  |  |  |  |  |  |  |  |
| Family Fibrobacteraceae | Breast Cancer | Simple mode | 0.016551139348768 | 0.0135049049122702 | 0.227529656692919 | 1.01668886826326 | 0.990130551565656 | 1.04395955989435 | |  |  |  |  |  |  |  |  |  |  |  |  |  |  |  |  |  |  |  |  |  |  |  |  |  |  |  |  |  |  |  |  |  |  |  |  |  |  |  |  |  |  |  |  |  |  |  |  |  |  |  |  |  |
| Family Fibrobacteraceae | Breast Cancer | Weighted mode | 0.00417058559201067 | 0.00823993999522093 | 0.615534835946269 | 1.00417929458709 | 0.988091774084545 | 1.0205287424963 | |  |  |  |  |  |  |  |  |  |  |  |  |  |  |  |  |  |  |  |  |  |  |  |  |  |  |  |  |  |  |  |  |  |  |  |  |  |  |  |  |  |  |  |  |  |  |  |  |  |  |  |  |  |
| Order Fibrobacterales | Breast Cancer | MR Egger | 0.0088971985628751 | 0.0119423632038247 | 0.460731057109539 | 1.00893689627949 | 0.985594927017754 | 1.03283167635029 | |  |  |  |  |  |  |  |  |  |  |  |  |  |  |  |  |  |  |  |  |  |  |  |  |  |  |  |  |  |  |  |  |  |  |  |  |  |  |  |  |  |  |  |  |  |  |  |  |  |  |  |  |  |
| Order Fibrobacterales | Breast Cancer | Weighted median | 0.000826736923282543 | 0.00726854084861465 | 0.909442475497516 | 1.00082707876445 | 0.986670038302004 | 1.01518724873005 | |  |  |  |  |  |  |  |  |  |  |  |  |  |  |  |  |  |  |  |  |  |  |  |  |  |  |  |  |  |  |  |  |  |  |  |  |  |  |  |  |  |  |  |  |  |  |  |  |  |  |  |  |  |
| Order Fibrobacterales | Breast Cancer | Inverse variance weighted | -0.004 | 0.00539624043030545 | 0.48131854567288 | 0.996207267525882 | 0.985726275115775 | 1.00679970182881 | |  |  |  |  |  |  |  |  |  |  |  |  |  |  |  |  |  |  |  |  |  |  |  |  |  |  |  |  |  |  |  |  |  |  |  |  |  |  |  |  |  |  |  |  |  |  |  |  |  |  |  |  |  |
| Order Fibrobacterales | Breast Cancer | Simple mode | 0.012024348618352 | 0.01259206717851 | 0.345356556656373 | 1.01209693172799 | 0.987423648617706 | 1.03738673936683 | |  |  |  |  |  |  |  |  |  |  |  |  |  |  |  |  |  |  |  |  |  |  |  |  |  |  |  |  |  |  |  |  |  |  |  |  |  |  |  |  |  |  |  |  |  |  |  |  |  |  |  |  |  |
| Order Fibrobacterales | Breast Cancer | Weighted mode | 0.00126103383289181 | 0.00788617181116816 | 0.873761119362167 | 1.00126182927038 | 0.98590442323464 | 1.01685845719679 | |  |  |  |  |  |  |  |  |  |  |  |  |  |  |  |  |  |  |  |  |  |  |  |  |  |  |  |  |  |  |  |  |  |  |  |  |  |  |  |  |  |  |  |  |  |  |  |  |  |  |  |  |  |
| Class Fibrobacteria | Breast Cancer | MR Egger | 0.00666797157784161 | 0.0112446879915058 | 0.556612073307521 | 1.00669025199452 | 0.984745923385305 | 1.02912359360361 | |  |  |  |  |  |  |  |  |  |  |  |  |  |  |  |  |  |  |  |  |  |  |  |  |  |  |  |  |  |  |  |  |  |  |  |  |  |  |  |  |  |  |  |  |  |  |  |  |  |  |  |  |  |
| Class Fibrobacteria | Breast Cancer | Weighted median | -0.003 | 0.00673598696247703 | 0.628622979938345 | 0.996747334053227 | 0.983674231975022 | 1.00999417860876 | |  |  |  |  |  |  |  |  |  |  |  |  |  |  |  |  |  |  |  |  |  |  |  |  |  |  |  |  |  |  |  |  |  |  |  |  |  |  |  |  |  |  |  |  |  |  |  |  |  |  |  |  |  |
| Class Fibrobacteria | Breast Cancer | Inverse variance weighted | -0.004 | 0.0050630246282995 | 0.427684639569854 | 0.995992264146036 | 0.986157385809725 | 1.00592522503315 | |  |  |  |  |  |  |  |  |  |  |  |  |  |  |  |  |  |  |  |  |  |  |  |  |  |  |  |  |  |  |  |  |  |  |  |  |  |  |  |  |  |  |  |  |  |  |  |  |  |  |  |  |  |
| Class Fibrobacteria | Breast Cancer | Simple mode | 0.0165501600790101 | 0.0116016312717628 | 0.161472447744005 | 1.01668787265109 | 0.993830075427228 | 1.04007139243743 | |  |  |  |  |  |  |  |  |  |  |  |  |  |  |  |  |  |  |  |  |  |  |  |  |  |  |  |  |  |  |  |  |  |  |  |  |  |  |  |  |  |  |  |  |  |  |  |  |  |  |  |  |  |
| Class Fibrobacteria | Breast Cancer | Weighted mode | -3E-04 | 0.00760073966361359 | 0.963666774249746 | 0.999651651435779 | 0.984869770745147 | 1.01465539292795 | |  |  |  |  |  |  |  |  |  |  |  |  |  |  |  |  |  |  |  |  |  |  |  |  |  |  |  |  |  |  |  |  |  |  |  |  |  |  |  |  |  |  |  |  |  |  |  |  |  |  |  |  |  |
| Species Megamonas funiformis | Breast Cancer | MR Egger | -0.01 | 0.0408723154402492 | 0.813046751218341 | 0.990315325208213 | 0.914075950165904 | 1.07291351792403 | |  |  |  |  |  |  |  |  |  |  |  |  |  |  |  |  |  |  |  |  |  |  |  |  |  |  |  |  |  |  |  |  |  |  |  |  |  |  |  |  |  |  |  |  |  |  |  |  |  |  |  |  |  |
| Species Megamonas funiformis | Breast Cancer | Weighted median | -0.013 | 0.027176294190424 | 0.64367779017444 | 0.987507939455152 | 0.93628414121549 | 1.04153417489373 | |  |  |  |  |  |  |  |  |  |  |  |  |  |  |  |  |  |  |  |  |  |  |  |  |  |  |  |  |  |  |  |  |  |  |  |  |  |  |  |  |  |  |  |  |  |  |  |  |  |  |  |  |  |
| Species Megamonas funiformis | Breast Cancer | Inverse variance weighted | -0.022 | 0.0183730718063683 | 0.225637583792989 | 0.977983818311238 | 0.943392011901171 | 1.01384401904267 | |  |  |  |  |  |  |  |  |  |  |  |  |  |  |  |  |  |  |  |  |  |  |  |  |  |  |  |  |  |  |  |  |  |  |  |  |  |  |  |  |  |  |  |  |  |  |  |  |  |  |  |  |  |
| Species Megamonas funiformis | Breast Cancer | Simple mode | 0.00206757686447112 | 0.0451398222304795 | 0.963694519108621 | 1.00206971577538 | 0.917221328166588 | 1.09476708013465 | |  |  |  |  |  |  |  |  |  |  |  |  |  |  |  |  |  |  |  |  |  |  |  |  |  |  |  |  |  |  |  |  |  |  |  |  |  |  |  |  |  |  |  |  |  |  |  |  |  |  |  |  |  |
| Species Megamonas funiformis | Breast Cancer | Weighted mode | -0.02 | 0.0316288851589203 | 0.533088680711663 | 0.980309853535868 | 0.921383259562845 | 1.04300506761483 | |  |  |  |  |  |  |  |  |  |  |  |  |  |  |  |  |  |  |  |  |  |  |  |  |  |  |  |  |  |  |  |  |  |  |  |  |  |  |  |  |  |  |  |  |  |  |  |  |  |  |  |  |  |
| Species Phascolarctobacterium sp003150755 | Breast Cancer | MR Egger | 0.000798043491418387 | 0.0440434891385225 | 0.985635950344246 | 1.00079836201285 | 0.918028176736367 | 1.09103117615445 | |  |  |  |  |  |  |  |  |  |  |  |  |  |  |  |  |  |  |  |  |  |  |  |  |  |  |  |  |  |  |  |  |  |  |  |  |  |  |  |  |  |  |  |  |  |  |  |  |  |  |  |  |  |
| Species Phascolarctobacterium sp003150755 | Breast Cancer | Weighted median | 0.00804008861537479 | 0.0285410954196881 | 0.778171887705577 | 1.00807249692485 | 0.953228668255042 | 1.06607175476221 | |  |  |  |  |  |  |  |  |  |  |  |  |  |  |  |  |  |  |  |  |  |  |  |  |  |  |  |  |  |  |  |  |  |  |  |  |  |  |  |  |  |  |  |  |  |  |  |  |  |  |  |  |  |
| Species Phascolarctobacterium sp003150755 | Breast Cancer | Inverse variance weighted | -0.019 | 0.0197985108852276 | 0.332500732885643 | 0.980996368552635 | 0.943657871732871 | 1.01981226876882 | |  |  |  |  |  |  |  |  |  |  |  |  |  |  |  |  |  |  |  |  |  |  |  |  |  |  |  |  |  |  |  |  |  |  |  |  |  |  |  |  |  |  |  |  |  |  |  |  |  |  |  |  |  |
| Species Phascolarctobacterium sp003150755 | Breast Cancer | Simple mode | 0.0318681367107243 | 0.0551275108732058 | 0.566450285210546 | 1.03238136312691 | 0.926647740957986 | 1.15017954700879 | |  |  |  |  |  |  |  |  |  |  |  |  |  |  |  |  |  |  |  |  |  |  |  |  |  |  |  |  |  |  |  |  |  |  |  |  |  |  |  |  |  |  |  |  |  |  |  |  |  |  |  |  |  |
| Species Phascolarctobacterium sp003150755 | Breast Cancer | Weighted mode | 0.0131483216613911 | 0.0339874526503545 | 0.700911803686045 | 1.0132351409346 | 0.947937141335798 | 1.08303114843465 | |  |  |  |  |  |  |  |  |  |  |  |  |  |  |  |  |  |  |  |  |  |  |  |  |  |  |  |  |  |  |  |  |  |  |  |  |  |  |  |  |  |  |  |  |  |  |  |  |  |  |  |  |  |
| Species Prevotella bivia | Breast Cancer | MR Egger | -0.018 | 0.0454249172884412 | 0.698710455900012 | 0.982443447751309 | 0.898754525779857 | 1.07392519352486 | |  |  |  |  |  |  |  |  |  |  |  |  |  |  |  |  |  |  |  |  |  |  |  |  |  |  |  |  |  |  |  |  |  |  |  |  |  |  |  |  |  |  |  |  |  |  |  |  |  |  |  |  |  |
| Species Prevotella bivia | Breast Cancer | Weighted median | -0.002 | 0.0305739650609688 | 0.958336885890786 | 0.998404068850841 | 0.940332091271276 | 1.06006239067124 | |  |  |  |  |  |  |  |  |  |  |  |  |  |  |  |  |  |  |  |  |  |  |  |  |  |  |  |  |  |  |  |  |  |  |  |  |  |  |  |  |  |  |  |  |  |  |  |  |  |  |  |  |  |
| Species Prevotella bivia | Breast Cancer | Inverse variance weighted | 0.00585181872389513 | 0.0204194208300535 | 0.774433138665084 | 1.00586897406207 | 0.966406963612193 | 1.04694236597688 | |  |  |  |  |  |  |  |  |  |  |  |  |  |  |  |  |  |  |  |  |  |  |  |  |  |  |  |  |  |  |  |  |  |  |  |  |  |  |  |  |  |  |  |  |  |  |  |  |  |  |  |  |  |
| Species Prevotella bivia | Breast Cancer | Simple mode | 0.0317255172353467 | 0.0515965110539626 | 0.542118152184164 | 1.03223413593749 | 0.932950023984707 | 1.14208401736649 | |  |  |  |  |  |  |  |  |  |  |  |  |  |  |  |  |  |  |  |  |  |  |  |  |  |  |  |  |  |  |  |  |  |  |  |  |  |  |  |  |  |  |  |  |  |  |  |  |  |  |  |  |  |
| Species Prevotella bivia | Breast Cancer | Weighted mode | 0.00437949659245473 | 0.0349488078932692 | 0.900904444459388 | 1.00438910060278 | 0.937892289170218 | 1.07560055355842 | |  |  |  |  |  |  |  |  |  |  |  |  |  |  |  |  |  |  |  |  |  |  |  |  |  |  |  |  |  |  |  |  |  |  |  |  |  |  |  |  |  |  |  |  |  |  |  |  |  |  |  |  |  |
| Species Prevotellamassilia | Breast Cancer | MR Egger | 0.0106681911512728 | 0.0675870657026396 | 0.875394416347414 | 1.01072529920169 | 0.885323240084476 | 1.15388999654959 | |  |  |  |  |  |  |  |  |  |  |  |  |  |  |  |  |  |  |  |  |  |  |  |  |  |  |  |  |  |  |  |  |  |  |  |  |  |  |  |  |  |  |  |  |  |  |  |  |  |  |  |  |  |
| Species Prevotellamassilia | Breast Cancer | Weighted median | -0.015 | 0.0464985826118204 | 0.748328364130657 | 0.985191927497911 | 0.899374256111173 | 1.07919826191586 | |  |  |  |  |  |  |  |  |  |  |  |  |  |  |  |  |  |  |  |  |  |  |  |  |  |  |  |  |  |  |  |  |  |  |  |  |  |  |  |  |  |  |  |  |  |  |  |  |  |  |  |  |  |
| Species Prevotellamassilia | Breast Cancer | Inverse variance weighted | -0.017 | 0.0303356494927144 | 0.576175274542969 | 0.983185935568241 | 0.926431751582583 | 1.04341694058726 | |  |  |  |  |  |  |  |  |  |  |  |  |  |  |  |  |  |  |  |  |  |  |  |  |  |  |  |  |  |  |  |  |  |  |  |  |  |  |  |  |  |  |  |  |  |  |  |  |  |  |  |  |  |
| Species Prevotellamassilia | Breast Cancer | Simple mode | -0.124 | 0.0835682115827928 | 0.146882591226019 | 0.883710484529884 | 0.750196977043536 | 1.04098556028002 | |  |  |  |  |  |  |  |  |  |  |  |  |  |  |  |  |  |  |  |  |  |  |  |  |  |  |  |  |  |  |  |  |  |  |  |  |  |  |  |  |  |  |  |  |  |  |  |  |  |  |  |  |  |
| Species Prevotellamassilia | Breast Cancer | Weighted mode | -0.011 | 0.0539634056182593 | 0.843414530449432 | 0.98932860937398 | 0.890032755253392 | 1.0997023329183 | |  |  |  |  |  |  |  |  |  |  |  |  |  |  |  |  |  |  |  |  |  |  |  |  |  |  |  |  |  |  |  |  |  |  |  |  |  |  |  |  |  |  |  |  |  |  |  |  |  |  |  |  |  |
| Genus RUG147 | Breast Cancer | MR Egger | 0.00416448651940233 | 0.0250337275410897 | 0.868737034046742 | 1.00417317004334 | 0.95609153800709 | 1.05467281672293 | |  |  |  |  |  |  |  |  |  |  |  |  |  |  |  |  |  |  |  |  |  |  |  |  |  |  |  |  |  |  |  |  |  |  |  |  |  |  |  |  |  |  |  |  |  |  |  |  |  |  |  |  |  |
| Genus RUG147 | Breast Cancer | Weighted median | -0.009 | 0.014916800544712 | 0.52987266303654 | 0.990673028375633 | 0.962128107544767 | 1.02006483487469 | |  |  |  |  |  |  |  |  |  |  |  |  |  |  |  |  |  |  |  |  |  |  |  |  |  |  |  |  |  |  |  |  |  |  |  |  |  |  |  |  |  |  |  |  |  |  |  |  |  |  |  |  |  |
| Genus RUG147 | Breast Cancer | Inverse variance weighted | 0.00618968250296884 | 0.0111128262715062 | 0.577537372680904 | 1.00620887817231 | 0.984529460533895 | 1.02836567832489 | |  |  |  |  |  |  |  |  |  |  |  |  |  |  |  |  |  |  |  |  |  |  |  |  |  |  |  |  |  |  |  |  |  |  |  |  |  |  |  |  |  |  |  |  |  |  |  |  |  |  |  |  |  |
| Genus RUG147 | Breast Cancer | Simple mode | 0.0202789752497978 | 0.0311532578644835 | 0.518805826892846 | 1.02048599065383 | 0.960038961772053 | 1.08473895184265 | |  |  |  |  |  |  |  |  |  |  |  |  |  |  |  |  |  |  |  |  |  |  |  |  |  |  |  |  |  |  |  |  |  |  |  |  |  |  |  |  |  |  |  |  |  |  |  |  |  |  |  |  |  |
| Genus RUG147 | Breast Cancer | Weighted mode | -0.029 | 0.0240632666599058 | 0.228152986173806 | 0.970978586158988 | 0.926246513041304 | 1.01787094634629 | |  |  |  |  |  |  |  |  |  |  |  |  |  |  |  |  |  |  |  |  |  |  |  |  |  |  |  |  |  |  |  |  |  |  |  |  |  |  |  |  |  |  |  |  |  |  |  |  |  |  |  |  |  |
| Species UBA7177 sp002491225 | Breast Cancer | MR Egger | -0.003 | 0.0212739616590241 | 0.876382044391582 | 0.996674421988941 | 0.955970633128234 | 1.03911131683659 | |  |  |  |  |  |  |  |  |  |  |  |  |  |  |  |  |  |  |  |  |  |  |  |  |  |  |  |  |  |  |  |  |  |  |  |  |  |  |  |  |  |  |  |  |  |  |  |  |  |  |  |  |  |
| Species UBA7177 sp002491225 | Breast Cancer | Weighted median | 0.0102964276167774 | 0.0138715419307095 | 0.457923843517113 | 1.01034961822862 | 0.983250071878336 | 1.03819606044335 | |  |  |  |  |  |  |  |  |  |  |  |  |  |  |  |  |  |  |  |  |  |  |  |  |  |  |  |  |  |  |  |  |  |  |  |  |  |  |  |  |  |  |  |  |  |  |  |  |  |  |  |  |  |
| Species UBA7177 sp002491225 | Breast Cancer | Inverse variance weighted | -1E-03 | 0.00944469336821156 | 0.916929487334482 | 0.999015382225199 | 0.980692129671715 | 1.01768098644439 | |  |  |  |  |  |  |  |  |  |  |  |  |  |  |  |  |  |  |  |  |  |  |  |  |  |  |  |  |  |  |  |  |  |  |  |  |  |  |  |  |  |  |  |  |  |  |  |  |  |  |  |  |  |
| Species UBA7177 sp002491225 | Breast Cancer | Simple mode | 0.0109832652804469 | 0.0263600175043542 | 0.67915186628455 | 1.01104380276865 | 0.96013404983065 | 1.06465297350635 | |  |  |  |  |  |  |  |  |  |  |  |  |  |  |  |  |  |  |  |  |  |  |  |  |  |  |  |  |  |  |  |  |  |  |  |  |  |  |  |  |  |  |  |  |  |  |  |  |  |  |  |  |  |
| Species UBA7177 sp002491225 | Breast Cancer | Weighted mode | 0.0109832652804469 | 0.0169652873531186 | 0.52107390148473 | 1.01104380276865 | 0.977977419233994 | 1.04522819342552 | |  |  |  |  |  |  |  |  |  |  |  |  |  |  |  |  |  |  |  |  |  |  |  |  |  |  |  |  |  |  |  |  |  |  |  |  |  |  |  |  |  |  |  |  |  |  |  |  |  |  |  |  |  |
| Genus Veillonella | Breast Cancer | MR Egger | 0.0414552987291502 | 0.0605375000592736 | 0.497528097133275 | 1.04232656748786 | 0.925706359138307 | 1.17363855456534 | |  |  |  |  |  |  |  |  |  |  |  |  |  |  |  |  |  |  |  |  |  |  |  |  |  |  |  |  |  |  |  |  |  |  |  |  |  |  |  |  |  |  |  |  |  |  |  |  |  |  |  |  |  |
| Genus Veillonella | Breast Cancer | Weighted median | -0.046 | 0.0458555458908825 | 0.320266988762902 | 0.955446740242369 | 0.873320097103906 | 1.04529653728003 | |  |  |  |  |  |  |  |  |  |  |  |  |  |  |  |  |  |  |  |  |  |  |  |  |  |  |  |  |  |  |  |  |  |  |  |  |  |  |  |  |  |  |  |  |  |  |  |  |  |  |  |  |  |
| Genus Veillonella | Breast Cancer | Inverse variance weighted | -0.033 | 0.0272129315856082 | 0.219537141368758 | 0.967141037006709 | 0.91690786145472 | 1.02012625781004 | |  |  |  |  |  |  |  |  |  |  |  |  |  |  |  |  |  |  |  |  |  |  |  |  |  |  |  |  |  |  |  |  |  |  |  |  |  |  |  |  |  |  |  |  |  |  |  |  |  |  |  |  |  |
| Genus Veillonella | Breast Cancer | Simple mode | -0.029 | 0.0928604815256373 | 0.757319707433886 | 0.971521954805051 | 0.809856704219062 | 1.16545915314534 | |  |  |  |  |  |  |  |  |  |  |  |  |  |  |  |  |  |  |  |  |  |  |  |  |  |  |  |  |  |  |  |  |  |  |  |  |  |  |  |  |  |  |  |  |  |  |  |  |  |  |  |  |  |
| Genus Veillonella | Breast Cancer | Weighted mode | -0.043 | 0.0701772626945623 | 0.545951418881849 | 0.958160749900566 | 0.835030395850182 | 1.09944742995287 | |  |  |  |  |  |  |  |  |  |  |  |  |  |  |  |  |  |  |  |  |  |  |  |  |  |  |  |  |  |  |  |  |  |  |  |  |  |  |  |  |  |  |  |  |  |  |  |  |  |  |  |  |  |

| **Table S4.** Sensitivity analysis of Gut microbiota and Breast cancer | |  |  |  |  |  |  |  |  |  | |  |  |  |  |  |  |  |  |  |  |  |  |  |  |  |  |  |  |  |  |  |  |  |  |  |  |  |  |  |  |  |  |  |  |  |  |  |  |  |  |  |  |  |  |  |  |  |  |  |  |  |
| --- | --- | --- | --- | --- | --- | --- | --- | --- | --- | --- | --- | --- | --- | --- | --- | --- | --- | --- | --- | --- | --- | --- | --- | --- | --- | --- | --- | --- | --- | --- | --- | --- | --- | --- | --- | --- | --- | --- | --- | --- | --- | --- | --- | --- | --- | --- | --- | --- | --- | --- | --- | --- | --- | --- | --- | --- | --- | --- | --- | --- | --- | --- |
|  |  |  |  |  |  |  |  |  |  |  |  |  |  |  |  |  |  |  |  |  |  |  |  |  |  |  |  |  |  |  |  |  |  |  |  |  |  |  |  |  |  |  |  |  |  |  |  |  |  |  |  |  |  |  |  |  |  |  |  |  |  |  |
| **Heterogeneity and pleiotropy in MR analyses** | | | | | | | | |  |  |  |  |  |  |  |  |  |  |  |  |  |  |  |  |  |  |  |  |  |  |  |  |  |  |  |  |  |  |  |  |  |  |  |  |  |  |  |  |  |  |  |  |  |  |  |  |  |  |  |  |  |  |
| Outcome | Exposure | Method | NO.of SNPs | Heterogeneity | | | Pleiotropy | | |  |  |  |  |  |  |  |  |  |  |  |  |  |  |  |  |  |  |  |  |  |  |  |  |  |  |  |  |  |  |  |  |  |  |  |  |  |  |  |  |  |  |  |  |  |  |  |  |  |  |  |  |  |
|  |  |  |  | Q | Q df | Q P-value | MR-egger regression | | |  |  |  |  |  |  |  |  |  |  |  |  |  |  |  |  |  |  |  |  |  |  |  |  |  |  |  |  |  |  |  |  |  |  |  |  |  |  |  |  |  |  |  |  |  |  |  |  |  |  |  |  |  |
|  |  |  |  |  |  |  | Egger intercept | Se | P-value |  |  |  |  |  |  |  |  |  |  |  |  |  |  |  |  |  |  |  |  |  |  |  |  |  |  |  |  |  |  |  |  |  |  |  |  |  |  |  |  |  |  |  |  |  |  |  |  |  |  |  |  |  |
| Breast Cancer | Phylum Actinobacteriota | MR Egger | 13 | 8.22817 | 11 | 0.69273094 | -0.007624552 | 0.012100456 | 0.54149 |  |  |  |  |  |  |  |  |  |  |  |  |  |  |  |  |  |  |  |  |  |  |  |  |  |  |  |  |  |  |  |  |  |  |  |  |  |  |  |  |  |  |  |  |  |  |  |  |  |  |  |  |  |
| Breast Cancer | Phylum Actinobacteriota | Inverse variance weighted | 13 | 8.6252 | 12 | 0.7345661 |  |  |  |  |  |  |  |  |  |  |  |  |  |  |  |  |  |  |  |  |  |  |  |  |  |  |  |  |  |  |  |  |  |  |  |  |  |  |  |  |  |  |  |  |  |  |  |  |  |  |  |  |  |  |  |  |
| Breast Cancer | Genus An7 | MR Egger | 15 | 15.682 | 13 | 0.2667301 | -0.013466867 | 0.011830968 | 0.27555 |  |  |  |  |  |  |  |  |  |  |  |  |  |  |  |  |  |  |  |  |  |  |  |  |  |  |  |  |  |  |  |  |  |  |  |  |  |  |  |  |  |  |  |  |  |  |  |  |  |  |  |  |  |
| Breast Cancer | Genus An7 | Inverse variance weighted | 15 | 17.2449 | 14 | 0.24335946 |  |  |  |  |  |  |  |  |  |  |  |  |  |  |  |  |  |  |  |  |  |  |  |  |  |  |  |  |  |  |  |  |  |  |  |  |  |  |  |  |  |  |  |  |  |  |  |  |  |  |  |  |  |  |  |  |
| Breast Cancer | Species Bacteroides A plebeius A | MR Egger | 21 | 12.9086 | 19 | 0.84321851 | 0.015592897 | 0.009158457 | 0.10495 |  |  |  |  |  |  |  |  |  |  |  |  |  |  |  |  |  |  |  |  |  |  |  |  |  |  |  |  |  |  |  |  |  |  |  |  |  |  |  |  |  |  |  |  |  |  |  |  |  |  |  |  |  |
| Breast Cancer | Species Bacteroides A plebeius A | Inverse variance weighted | 21 | 15.8073 | 20 | 0.72850431 |  |  |  |  |  |  |  |  |  |  |  |  |  |  |  |  |  |  |  |  |  |  |  |  |  |  |  |  |  |  |  |  |  |  |  |  |  |  |  |  |  |  |  |  |  |  |  |  |  |  |  |  |  |  |  |  |
| Breast Cancer | Species Bifidobacterium adolescentis | MR Egger | 22 | 18.9081 | 20 | 0.52780425 | -0.008526597 | 0.008623169 | 0.33457 |  |  |  |  |  |  |  |  |  |  |  |  |  |  |  |  |  |  |  |  |  |  |  |  |  |  |  |  |  |  |  |  |  |  |  |  |  |  |  |  |  |  |  |  |  |  |  |  |  |  |  |  |  |
| Breast Cancer | Species Bifidobacterium adolescentis | Inverse variance weighted | 22 | 19.8859 | 21 | 0.52850074 |  |  |  |  |  |  |  |  |  |  |  |  |  |  |  |  |  |  |  |  |  |  |  |  |  |  |  |  |  |  |  |  |  |  |  |  |  |  |  |  |  |  |  |  |  |  |  |  |  |  |  |  |  |  |  |  |
| Breast Cancer | Species Blautia sp001304935 | MR Egger | 16 | 8.99115 | 14 | 0.83161722 | -0.010761957 | 0.009981743 | 0.29919 |  |  |  |  |  |  |  |  |  |  |  |  |  |  |  |  |  |  |  |  |  |  |  |  |  |  |  |  |  |  |  |  |  |  |  |  |  |  |  |  |  |  |  |  |  |  |  |  |  |  |  |  |  |
| Breast Cancer | Species Blautia sp001304935 | Inverse variance weighted | 16 | 10.1536 | 15 | 0.80996929 |  |  |  |  |  |  |  |  |  |  |  |  |  |  |  |  |  |  |  |  |  |  |  |  |  |  |  |  |  |  |  |  |  |  |  |  |  |  |  |  |  |  |  |  |  |  |  |  |  |  |  |  |  |  |  |  |
| Breast Cancer | Species CAG-180 sp000432435 | MR Egger | 17 | 16.8285 | 15 | 0.32921941 | -0.020450856 | 0.015695589 | 0.21223 |  |  |  |  |  |  |  |  |  |  |  |  |  |  |  |  |  |  |  |  |  |  |  |  |  |  |  |  |  |  |  |  |  |  |  |  |  |  |  |  |  |  |  |  |  |  |  |  |  |  |  |  |  |
| Breast Cancer | Species CAG-180 sp000432435 | Inverse variance weighted | 17 | 18.7332 | 16 | 0.28274189 |  |  |  |  |  |  |  |  |  |  |  |  |  |  |  |  |  |  |  |  |  |  |  |  |  |  |  |  |  |  |  |  |  |  |  |  |  |  |  |  |  |  |  |  |  |  |  |  |  |  |  |  |  |  |  |  |
| Breast Cancer | Species CAG-841 sp002479075 | MR Egger | 14 | 10.8666 | 12 | 0.54038223 | 0.005194178 | 0.00973154 | 0.60326 |  |  |  |  |  |  |  |  |  |  |  |  |  |  |  |  |  |  |  |  |  |  |  |  |  |  |  |  |  |  |  |  |  |  |  |  |  |  |  |  |  |  |  |  |  |  |  |  |  |  |  |  |  |
| Breast Cancer | Species CAG-841 sp002479075 | Inverse variance weighted | 14 | 11.1515 | 13 | 0.59812665 |  |  |  |  |  |  |  |  |  |  |  |  |  |  |  |  |  |  |  |  |  |  |  |  |  |  |  |  |  |  |  |  |  |  |  |  |  |  |  |  |  |  |  |  |  |  |  |  |  |  |  |  |  |  |  |  |
| Breast Cancer | Species CHKCI006 sp900018345 | MR Egger | 21 | 17.1539 | 19 | 0.57944004 | -0.003609355 | 0.007309125 | 0.6271 |  |  |  |  |  |  |  |  |  |  |  |  |  |  |  |  |  |  |  |  |  |  |  |  |  |  |  |  |  |  |  |  |  |  |  |  |  |  |  |  |  |  |  |  |  |  |  |  |  |  |  |  |  |
| Breast Cancer | Species CHKCI006 sp900018345 | Inverse variance weighted | 21 | 17.3978 | 20 | 0.62701324 |  |  |  |  |  |  |  |  |  |  |  |  |  |  |  |  |  |  |  |  |  |  |  |  |  |  |  |  |  |  |  |  |  |  |  |  |  |  |  |  |  |  |  |  |  |  |  |  |  |  |  |  |  |  |  |  |
| Breast Cancer | Family Fibrobacteraceae | MR Egger | 16 | 7.29763 | 14 | 0.92262839 | 0.015899146 | 0.00822575 | 0.07374 |  |  |  |  |  |  |  |  |  |  |  |  |  |  |  |  |  |  |  |  |  |  |  |  |  |  |  |  |  |  |  |  |  |  |  |  |  |  |  |  |  |  |  |  |  |  |  |  |  |  |  |  |  |
| Breast Cancer | Family Fibrobacteraceae | Inverse variance weighted | 16 | 11.0335 | 15 | 0.75021291 |  |  |  |  |  |  |  |  |  |  |  |  |  |  |  |  |  |  |  |  |  |  |  |  |  |  |  |  |  |  |  |  |  |  |  |  |  |  |  |  |  |  |  |  |  |  |  |  |  |  |  |  |  |  |  |  |
| Breast Cancer | Order Fibrobacterales | MR Egger | 15 | 7.29763 | 14 | 0.92262839 | -0.003609355 | 0.007309125 | 0.6271 |  |  |  |  |  |  |  |  |  |  |  |  |  |  |  |  |  |  |  |  |  |  |  |  |  |  |  |  |  |  |  |  |  |  |  |  |  |  |  |  |  |  |  |  |  |  |  |  |  |  |  |  |  |
| Breast Cancer | Order Fibrobacterales | Inverse variance weighted | 15 | 11.0335 | 15 | 0.75021291 |  |  |  |  |  |  |  |  |  |  |  |  |  |  |  |  |  |  |  |  |  |  |  |  |  |  |  |  |  |  |  |  |  |  |  |  |  |  |  |  |  |  |  |  |  |  |  |  |  |  |  |  |  |  |  |  |
| Breast Cancer | Class Fibrobacteria | MR Egger | 20 | 14.3748 | 18 | 0.70435463 | 0.009354856 | 0.007260744 | 0.21392 |  |  |  |  |  |  |  |  |  |  |  |  |  |  |  |  |  |  |  |  |  |  |  |  |  |  |  |  |  |  |  |  |  |  |  |  |  |  |  |  |  |  |  |  |  |  |  |  |  |  |  |  |  |
| Breast Cancer | Class Fibrobacteria | Inverse variance weighted | 20 | 16.0348 | 19 | 0.65495645 |  |  |  |  |  |  |  |  |  |  |  |  |  |  |  |  |  |  |  |  |  |  |  |  |  |  |  |  |  |  |  |  |  |  |  |  |  |  |  |  |  |  |  |  |  |  |  |  |  |  |  |  |  |  |  |  |
| Breast Cancer | Species Megamonas funiformis | MR Egger | 39 | 24.2391 | 37 | 0.94731948 | -0.005412813 | 0.005545668 | 0.33538 |  |  |  |  |  |  |  |  |  |  |  |  |  |  |  |  |  |  |  |  |  |  |  |  |  |  |  |  |  |  |  |  |  |  |  |  |  |  |  |  |  |  |  |  |  |  |  |  |  |  |  |  |  |
| Breast Cancer | Species Megamonas funiformis | Inverse variance weighted | 39 | 25.1918 | 38 | 0.94498409 |  |  |  |  |  |  |  |  |  |  |  |  |  |  |  |  |  |  |  |  |  |  |  |  |  |  |  |  |  |  |  |  |  |  |  |  |  |  |  |  |  |  |  |  |  |  |  |  |  |  |  |  |  |  |  |  |
| Breast Cancer | Species Prevotella bivia | MR Egger | 14 | 10.0538 | 12 | 0.61124046 | 0.011558709 | 0.008994107 | 0.22299 |  |  |  |  |  |  |  |  |  |  |  |  |  |  |  |  |  |  |  |  |  |  |  |  |  |  |  |  |  |  |  |  |  |  |  |  |  |  |  |  |  |  |  |  |  |  |  |  |  |  |  |  |  |
| Breast Cancer | Species Prevotella bivia | Inverse variance weighted | 14 | 11.7054 | 13 | 0.55193688 |  |  |  |  |  |  |  |  |  |  |  |  |  |  |  |  |  |  |  |  |  |  |  |  |  |  |  |  |  |  |  |  |  |  |  |  |  |  |  |  |  |  |  |  |  |  |  |  |  |  |  |  |  |  |  |  |
| Breast Cancer | Species Prevotellamassilia | MR Egger | 14 | 10.6914 | 12 | 0.55553626 | -0.021465667 | 0.012280752 | 0.10599 |  |  |  |  |  |  |  |  |  |  |  |  |  |  |  |  |  |  |  |  |  |  |  |  |  |  |  |  |  |  |  |  |  |  |  |  |  |  |  |  |  |  |  |  |  |  |  |  |  |  |  |  |  |
| Breast Cancer | Species Prevotellamassilia | Inverse variance weighted | 14 | 13.7466 | 13 | 0.39193062 |  |  |  |  |  |  |  |  |  |  |  |  |  |  |  |  |  |  |  |  |  |  |  |  |  |  |  |  |  |  |  |  |  |  |  |  |  |  |  |  |  |  |  |  |  |  |  |  |  |  |  |  |  |  |  |  |
| Breast Cancer | Genus RUG147 | MR Egger | 6 | 6.8237 | 4 | 0.14550323 | -0.017002468 | 0.023359072 | 0.50701 |  |  |  |  |  |  |  |  |  |  |  |  |  |  |  |  |  |  |  |  |  |  |  |  |  |  |  |  |  |  |  |  |  |  |  |  |  |  |  |  |  |  |  |  |  |  |  |  |  |  |  |  |  |
| Breast Cancer | Genus RUG147 | Inverse variance weighted | 6 | 7.7275 | 5 | 0.17190666 |  |  |  |  |  |  |  |  |  |  |  |  |  |  |  |  |  |  |  |  |  |  |  |  |  |  |  |  |  |  |  |  |  |  |  |  |  |  |  |  |  |  |  |  |  |  |  |  |  |  |  |  |  |  |  |  |
| Breast Cancer | Species UBA7177 sp002491225 | MR Egger | 20 | 17.8987 | 18 | 0.46234537 | 0.008017555 | 0.008972458 | 0.38334 |  |  |  |  |  |  |  |  |  |  |  |  |  |  |  |  |  |  |  |  |  |  |  |  |  |  |  |  |  |  |  |  |  |  |  |  |  |  |  |  |  |  |  |  |  |  |  |  |  |  |  |  |  |
| Breast Cancer | Species UBA7177 sp002491225 | Inverse variance weighted | 20 | 18.6972 | 19 | 0.4764123 |  |  |  |  |  |  |  |  |  |  |  |  |  |  |  |  |  |  |  |  |  |  |  |  |  |  |  |  |  |  |  |  |  |  |  |  |  |  |  |  |  |  |  |  |  |  |  |  |  |  |  |  |  |  |  |  |
| Breast Cancer | Genus Veillonella | MR Egger | 8 | 5.51354 | 6 | 0.47982225 | 0.009765616 | 0.018445537 | 0.61551 |  |  |  |  |  |  |  |  |  |  |  |  |  |  |  |  |  |  |  |  |  |  |  |  |  |  |  |  |  |  |  |  |  |  |  |  |  |  |  |  |  |  |  |  |  |  |  |  |  |  |  |  |  |
| Breast Cancer | Genus Veillonella | Inverse variance weighted | 8 | 5.79383 | 7 | 0.56401844 |  |  |  |  |  |  |  |  |  |  |  |  |  |  |  |  |  |  |  |  |  |  |  |  |  |  |  |  |  |  |  |  |  |  |  |  |  |  |  |  |  |  |  |  |  |  |  |  |  |  |  |  |  |  |  |  |
| Breast Cancer | Species Phascolarctobacterium sp003150755 | MR Egger | 18 | 27.9259 | 16 | 0.03227037 | 0.007456265 | 0.012095197 | 0.54626 |  |  |  |  |  |  |  |  |  |  |  |  |  |  |  |  |  |  |  |  |  |  |  |  |  |  |  |  |  |  |  |  |  |  |  |  |  |  |  |  |  |  |  |  |  |  |  |  |  |  |  |  |  |
| Breast Cancer | Species Phascolarctobacterium sp003150755 | Inverse variance weighted | 18 | 28.5892 | 17 | 0.03850764 |  |  |  |  |  |  |  |  |  |  |  |  |  |  |  |  |  |  |  |  |  |  |  |  |  |  |  |  |  |  |  |  |  |  |  |  |  |  |  |  |  |  |  |  |  |  |  |  |  |  |  |  |  |  |  |  |

| **Causal estimations of Immune cell traits on Breast cancer identified in MR methods** | | | | | | | | |  |
| --- | --- | --- | --- | --- | --- | --- | --- | --- | --- |
| Outcome | Exposure | Method | NO.of SNPs | Beta | Se | Pvalue | or | or_lci95 | or_uci95 |
| Breast Cancer | CD24+ CD27+ AC | MR Egger | 22 | -0.068970014 | 0.042459305 | 0.119951078 | 0.933354668 | 0.858824872 | 1.014352243 |
| Breast Cancer | CD24+ CD27+ AC | Weighted median | 22 | -0.053530182 | 0.027566775 | 0.052156843 | 0.947877332 | 0.898021687 | 1.000500822 |
| Breast Cancer | CD24+ CD27+ AC | Inverse variance weighted | 22 | -0.044089552 | 0.019544897 | 0.024082605 | 0.956868264 | 0.920905779 | 0.994235128 |
| Breast Cancer | CD24+ CD27+ AC | Simple mode | 22 | -0.045914488 | 0.047208199 | 0.341824814 | 0.955123633 | 0.870713264 | 1.047717075 |
| Breast Cancer | CD24+ CD27+ AC | Weighted mode | 22 | -0.05477972 | 0.041757939 | 0.203735015 | 0.946693663 | 0.872297032 | 1.027435447 |
| Breast Cancer | IgD- CD38br AC | MR Egger | 15 | 0.005899029 | 0.052207093 | 0.91176245 | 1.005916463 | 0.9080763 | 1.114298358 |
| Breast Cancer | IgD- CD38br AC | Weighted median | 15 | 0.051579143 | 0.030621248 | 0.092099896 | 1.052932515 | 0.991597001 | 1.118061956 |
| Breast Cancer | IgD- CD38br AC | Inverse variance weighted | 15 | 0.042680722 | 0.021605995 | 0.04822152 | 1.043604641 | 1.000333026 | 1.088748065 |
| Breast Cancer | IgD- CD38br AC | Simple mode | 15 | 0.042997219 | 0.042415931 | 0.327923578 | 1.043934992 | 0.960656854 | 1.134432407 |
| Breast Cancer | IgD- CD38br AC | Weighted mode | 15 | 0.05133516 | 0.033777256 | 0.150816108 | 1.052675649 | 0.985241724 | 1.124725023 |
| Breast Cancer | CD62L- myeloid DC AC | MR Egger | 15 | -0.005083913 | 0.026333237 | 0.849895022 | 0.994928989 | 0.94488027 | 1.047628703 |
| Breast Cancer | CD62L- myeloid DC AC | Weighted median | 15 | -0.012284407 | 0.020184962 | 0.542794907 | 0.987790739 | 0.949474189 | 1.027653573 |
| Breast Cancer | CD62L- myeloid DC AC | Inverse variance weighted | 15 | -0.035214326 | 0.015680789 | 0.024723319 | 0.965398484 | 0.936178915 | 0.995530041 |
| Breast Cancer | CD62L- myeloid DC AC | Simple mode | 15 | -0.039816373 | 0.041000485 | 0.347968822 | 0.960965882 | 0.886763179 | 1.04137773 |
| Breast Cancer | CD62L- myeloid DC AC | Weighted mode | 15 | -0.008741892 | 0.018901628 | 0.650833266 | 0.991296207 | 0.955243418 | 1.028709701 |
| Breast Cancer | Activated & secreting Treg AC | MR Egger | 11 | 0.117410785 | 0.052880242 | 0.053536533 | 1.124581296 | 1.013860691 | 1.247393356 |
| Breast Cancer | Activated & secreting Treg AC | Weighted median | 11 | 0.082904393 | 0.033997152 | 0.014745554 | 1.086437932 | 1.016403051 | 1.161298541 |
| Breast Cancer | Activated & secreting Treg AC | Inverse variance weighted | 11 | 0.077225777 | 0.0247383 | 0.001798045 | 1.080285953 | 1.02915565 | 1.133956501 |
| Breast Cancer | Activated & secreting Treg AC | Simple mode | 11 | 0.025456391 | 0.047866051 | 0.606458857 | 1.025783172 | 0.933923201 | 1.126678419 |
| Breast Cancer | Activated & secreting Treg AC | Weighted mode | 11 | 0.099124018 | 0.042021678 | 0.0400298 | 1.104203232 | 1.016902791 | 1.198998359 |
| Breast Cancer | EM DN (CD4-CD8-) %T cell | MR Egger | 21 | -0.108356675 | 0.061370446 | 0.093526058 | 0.897307492 | 0.795612715 | 1.012000839 |
| Breast Cancer | EM DN (CD4-CD8-) %T cell | Weighted median | 21 | -0.045673187 | 0.028829699 | 0.11313892 | 0.955354133 | 0.902867564 | 1.010891913 |
| Breast Cancer | EM DN (CD4-CD8-) %T cell | Inverse variance weighted | 21 | -0.068907089 | 0.021862189 | 0.001622206 | 0.9334134 | 0.894261554 | 0.974279361 |
| Breast Cancer | EM DN (CD4-CD8-) %T cell | Simple mode | 21 | -0.036710268 | 0.044770277 | 0.421898576 | 0.963955384 | 0.882973571 | 1.052364434 |
| Breast Cancer | EM DN (CD4-CD8-) %T cell | Weighted mode | 21 | -0.036710268 | 0.033848737 | 0.291027067 | 0.963955384 | 0.902078447 | 1.030076692 |
| Breast Cancer | CD4+ AC | MR Egger | 20 | -0.035065852 | 0.09255084 | 0.709207568 | 0.965541832 | 0.805360322 | 1.157582518 |
| Breast Cancer | CD4+ AC | Weighted median | 20 | -0.073514762 | 0.029283554 | 0.012057898 | 0.92912243 | 0.877296264 | 0.98401022 |
| Breast Cancer | CD4+ AC | Inverse variance weighted | 20 | -0.057237511 | 0.024004366 | 0.017104092 | 0.944369745 | 0.900967522 | 0.989862779 |
| Breast Cancer | CD4+ AC | Simple mode | 20 | -0.070359184 | 0.047055569 | 0.151277674 | 0.932058979 | 0.849941207 | 1.022110628 |
| Breast Cancer | CD4+ AC | Weighted mode | 20 | -0.07574286 | 0.042446295 | 0.090330159 | 0.927054559 | 0.85304959 | 1.007479712 |
| Breast Cancer | HLA DR+ CD4+ AC | MR Egger | 20 | -0.070110771 | 0.038727353 | 0.086965086 | 0.932290543 | 0.864143545 | 1.005811664 |
| Breast Cancer | HLA DR+ CD4+ AC | Weighted median | 20 | -0.029971879 | 0.023503035 | 0.202226291 | 0.970472824 | 0.926781148 | 1.016224277 |
| Breast Cancer | HLA DR+ CD4+ AC | Inverse variance weighted | 20 | -0.043322802 | 0.015543834 | 0.005317591 | 0.957602224 | 0.928867928 | 0.987225409 |
| Breast Cancer | HLA DR+ CD4+ AC | Simple mode | 20 | -0.074835826 | 0.045180817 | 0.114070456 | 0.92789581 | 0.849259719 | 1.013813106 |
| Breast Cancer | HLA DR+ CD4+ AC | Weighted mode | 20 | -0.016690513 | 0.035238647 | 0.641151894 | 0.983448002 | 0.917816076 | 1.053773188 |
| Breast Cancer | B cell %lymphocyte | MR Egger | 33 | -0.014763444 | 0.026161574 | 0.576598434 | 0.985345001 | 0.936093299 | 1.037188037 |
| Breast Cancer | B cell %lymphocyte | Weighted median | 33 | -0.030717564 | 0.017844902 | 0.085184871 | 0.969749427 | 0.936417766 | 1.004267524 |
| Breast Cancer | B cell %lymphocyte | Inverse variance weighted | 33 | -0.025875816 | 0.012733836 | 0.042148406 | 0.974456094 | 0.950436302 | 0.999082924 |
| Breast Cancer | B cell %lymphocyte | Simple mode | 33 | -0.04250083 | 0.031571569 | 0.187705839 | 0.95838967 | 0.900881902 | 1.019568445 |
| Breast Cancer | B cell %lymphocyte | Weighted mode | 33 | -0.035503956 | 0.026125639 | 0.183658022 | 0.965118917 | 0.916942779 | 1.015826227 |
| Breast Cancer | CD28+ CD45RA- CD8dim AC | MR Egger | 16 | 0.047484087 | 0.04226772 | 0.280157907 | 1.048629514 | 0.965257239 | 1.139202914 |
| Breast Cancer | CD28+ CD45RA- CD8dim AC | Weighted median | 16 | 0.019248801 | 0.029236918 | 0.510298394 | 1.019435254 | 0.962659456 | 1.079559578 |
| Breast Cancer | CD28+ CD45RA- CD8dim AC | Inverse variance weighted | 16 | 0.043897777 | 0.020537267 | 0.032559848 | 1.044875539 | 1.003651383 | 1.087792943 |
| Breast Cancer | CD28+ CD45RA- CD8dim AC | Simple mode | 16 | 0.022236591 | 0.046411841 | 0.638766897 | 1.022485667 | 0.933578135 | 1.119860137 |
| Breast Cancer | CD28+ CD45RA- CD8dim AC | Weighted mode | 16 | 0.020702887 | 0.034942976 | 0.562357437 | 1.020918679 | 0.953338403 | 1.093289586 |
| Breast Cancer | CD28- CD8br %CD8br | MR Egger | 24 | -0.002431489 | 0.046043174 | 0.958360779 | 0.997571465 | 0.911488675 | 1.091784083 |
| Breast Cancer | CD28- CD8br %CD8br | Weighted median | 24 | -0.048760887 | 0.027151287 | 0.072511062 | 0.952408836 | 0.903049952 | 1.004465576 |
| Breast Cancer | CD28- CD8br %CD8br | Inverse variance weighted | 24 | -0.04680271 | 0.019312567 | 0.015374594 | 0.954275648 | 0.918828912 | 0.991089854 |
| Breast Cancer | CD28- CD8br %CD8br | Simple mode | 24 | -0.04224813 | 0.040011861 | 0.301983897 | 0.958631885 | 0.886325178 | 1.036837398 |
| Breast Cancer | CD28- CD8br %CD8br | Weighted mode | 24 | -0.04224813 | 0.034000272 | 0.226541814 | 0.958631885 | 0.896830262 | 1.02469233 |
| Breast Cancer | CD28- DN (CD4-CD8-) %T cell | MR Egger | 24 | -0.061471122 | 0.066393112 | 0.36456143 | 0.940380102 | 0.825635719 | 1.071071317 |
| Breast Cancer | CD28- DN (CD4-CD8-) %T cell | Weighted median | 24 | -0.034520999 | 0.026297547 | 0.189281385 | 0.966068053 | 0.917535331 | 1.017167897 |
| Breast Cancer | CD28- DN (CD4-CD8-) %T cell | Inverse variance weighted | 24 | -0.037086169 | 0.01887319 | 0.049412083 | 0.9635931 | 0.928599612 | 0.999905287 |
| Breast Cancer | CD28- DN (CD4-CD8-) %T cell | Simple mode | 24 | -0.038051974 | 0.048510529 | 0.440806241 | 0.962662906 | 0.875349004 | 1.058686154 |
| Breast Cancer | CD28- DN (CD4-CD8-) %T cell | Weighted mode | 24 | -0.016346672 | 0.041505508 | 0.697324222 | 0.983786209 | 0.906923235 | 1.067163425 |
| Breast Cancer | CD24 on IgD+ CD38br | MR Egger | 22 | -0.035862124 | 0.032335218 | 0.280557222 | 0.964773303 | 0.905526125 | 1.027896933 |
| Breast Cancer | CD24 on IgD+ CD38br | Weighted median | 22 | -0.033152953 | 0.024662948 | 0.178870384 | 0.967390583 | 0.921739778 | 1.015302326 |
| Breast Cancer | CD24 on IgD+ CD38br | Inverse variance weighted | 22 | -0.032921392 | 0.015528249 | 0.033997877 | 0.967614619 | 0.938608556 | 0.997517064 |
| Breast Cancer | CD24 on IgD+ CD38br | Simple mode | 22 | -0.038436952 | 0.041486509 | 0.364716049 | 0.962292373 | 0.887141742 | 1.043809086 |
| Breast Cancer | CD24 on IgD+ CD38br | Weighted mode | 22 | -0.03616181 | 0.024786493 | 0.159380317 | 0.964484218 | 0.918748063 | 1.012497161 |
| Breast Cancer | CD38 on IgD+ CD24- | MR Egger | 15 | 0.067501511 | 0.036540035 | 0.087577442 | 1.069831876 | 0.995891505 | 1.14926198 |
| Breast Cancer | CD38 on IgD+ CD24- | Weighted median | 15 | 0.052524451 | 0.0260531 | 0.043794351 | 1.053928331 | 1.001461442 | 1.109143977 |
| Breast Cancer | CD38 on IgD+ CD24- | Inverse variance weighted | 15 | 0.041192273 | 0.019598544 | 0.035570564 | 1.042052445 | 1.002782992 | 1.082859708 |
| Breast Cancer | CD38 on IgD+ CD24- | Simple mode | 15 | 0.063624408 | 0.041966319 | 0.15174898 | 1.065692058 | 0.981542881 | 1.157055474 |
| Breast Cancer | CD38 on IgD+ CD24- | Weighted mode | 15 | 0.057999575 | 0.03581516 | 0.127657715 | 1.059714546 | 0.987875957 | 1.136777255 |
| Breast Cancer | IgD on unsw mem | MR Egger | 20 | -0.00173019 | 0.03985684 | 0.965852549 | 0.998271306 | 0.923255197 | 1.079382606 |
| Breast Cancer | IgD on unsw mem | Weighted median | 20 | -0.046224789 | 0.024888579 | 0.063272876 | 0.954827303 | 0.90936711 | 1.002560097 |
| Breast Cancer | IgD on unsw mem | Inverse variance weighted | 20 | -0.043355021 | 0.018736287 | 0.020669906 | 0.957571371 | 0.923044214 | 0.993390043 |
| Breast Cancer | IgD on unsw mem | Simple mode | 20 | -0.059539364 | 0.035644988 | 0.111243269 | 0.942198444 | 0.878619335 | 1.010378297 |
| Breast Cancer | IgD on unsw mem | Weighted mode | 20 | -0.034797417 | 0.031079187 | 0.276823117 | 0.965801052 | 0.908725125 | 1.026461849 |
| Breast Cancer | HVEM on EM CD4+ | MR Egger | 19 | -0.01996509 | 0.035484561 | 0.581023636 | 0.980232893 | 0.914374706 | 1.050834541 |
| Breast Cancer | HVEM on EM CD4+ | Weighted median | 19 | -0.01744677 | 0.016052414 | 0.277097485 | 0.982704544 | 0.952267305 | 1.014114645 |
| Breast Cancer | HVEM on EM CD4+ | Inverse variance weighted | 19 | -0.024111189 | 0.011508641 | 0.036166469 | 0.976177163 | 0.954404087 | 0.998446955 |
| Breast Cancer | HVEM on EM CD4+ | Simple mode | 19 | -0.015505967 | 0.027992539 | 0.586441659 | 0.984613632 | 0.932047645 | 1.040144255 |
| Breast Cancer | HVEM on EM CD4+ | Weighted mode | 19 | -0.0148421 | 0.023039593 | 0.527570387 | 0.985267501 | 0.941764813 | 1.030779697 |
| Breast Cancer | CD127 on CD45RA- CD4 not Treg | MR Egger | 15 | 0.00895567 | 0.0633547 | 0.889754463 | 1.008995892 | 0.891170473 | 1.142399509 |
| Breast Cancer | CD127 on CD45RA- CD4 not Treg | Weighted median | 15 | 0.027828006 | 0.030036047 | 0.354192954 | 1.028218822 | 0.969434231 | 1.090567996 |
| Breast Cancer | CD127 on CD45RA- CD4 not Treg | Inverse variance weighted | 15 | 0.048509165 | 0.022584756 | 0.031723927 | 1.049704993 | 1.004252057 | 1.09721515 |
| Breast Cancer | CD127 on CD45RA- CD4 not Treg | Simple mode | 15 | 0.037431481 | 0.051107436 | 0.476003561 | 1.038140863 | 0.939188483 | 1.14751881 |
| Breast Cancer | CD127 on CD45RA- CD4 not Treg | Weighted mode | 15 | 0.025247818 | 0.036611099 | 0.501703517 | 1.025569243 | 0.954555076 | 1.101866513 |
| Breast Cancer | CD127 on CD28+ CD4+ | MR Egger | 18 | 0.050129968 | 0.037289652 | 0.197589844 | 1.051407737 | 0.977303775 | 1.131130625 |
| Breast Cancer | CD127 on CD28+ CD4+ | Weighted median | 18 | 0.025903401 | 0.023249899 | 0.265223593 | 1.026241809 | 0.980525721 | 1.074089367 |
| Breast Cancer | CD127 on CD28+ CD4+ | Inverse variance weighted | 18 | 0.033320774 | 0.016315776 | 0.04112746 | 1.033882129 | 1.001342755 | 1.067478894 |
| Breast Cancer | CD127 on CD28+ CD4+ | Simple mode | 18 | 0.020532982 | 0.031244758 | 0.519873212 | 1.020745234 | 0.960110646 | 1.085209123 |
| Breast Cancer | CD127 on CD28+ CD4+ | Weighted mode | 18 | 0.022576637 | 0.025249828 | 0.383736456 | 1.022833418 | 0.973445903 | 1.074726595 |
| Breast Cancer | FSC-A on NK | MR Egger | 16 | 0.083097643 | 0.062348705 | 0.203886695 | 1.086647906 | 0.961648942 | 1.227894734 |
| Breast Cancer | FSC-A on NK | Weighted median | 16 | 0.037557531 | 0.029349752 | 0.20066681 | 1.038271728 | 0.980230057 | 1.099750181 |
| Breast Cancer | FSC-A on NK | Inverse variance weighted | 16 | 0.044516658 | 0.020376721 | 0.028912166 | 1.045522393 | 1.00458878 | 1.088123912 |
| Breast Cancer | FSC-A on NK | Simple mode | 16 | 0.038561961 | 0.04245039 | 0.37803063 | 1.039315124 | 0.956340934 | 1.129488332 |
| Breast Cancer | FSC-A on NK | Weighted mode | 16 | 0.03420469 | 0.035929616 | 0.356190576 | 1.034796397 | 0.964430644 | 1.110296102 |
| Breast Cancer | HLA DR on CD14+ CD16- monocyte | MR Egger | 19 | -0.026919094 | 0.02477086 | 0.29232779 | 0.973439995 | 0.927307569 | 1.021867454 |
| Breast Cancer | HLA DR on CD14+ CD16- monocyte | Weighted median | 19 | -0.026177995 | 0.015675235 | 0.094914903 | 0.974161678 | 0.944687159 | 1.004555811 |
| Breast Cancer | HLA DR on CD14+ CD16- monocyte | Inverse variance weighted | 19 | -0.025967019 | 0.012643181 | 0.039991583 | 0.974367225 | 0.9505185 | 0.99881432 |
| Breast Cancer | HLA DR on CD14+ CD16- monocyte | Simple mode | 19 | -0.025708972 | 0.032001807 | 0.432247948 | 0.974618689 | 0.915364883 | 1.03770814 |
| Breast Cancer | HLA DR on CD14+ CD16- monocyte | Weighted mode | 19 | -0.028137331 | 0.015322 | 0.082871121 | 0.972254837 | 0.943491001 | 1.001895585 |
| Breast Cancer | CD11c on myeloid DC | MR Egger | 28 | -0.024031272 | 0.041042285 | 0.563243195 | 0.97625518 | 0.900798081 | 1.058033088 |
| Breast Cancer | CD11c on myeloid DC | Weighted median | 28 | -0.027544838 | 0.022736797 | 0.225717171 | 0.972831062 | 0.93042951 | 1.017164937 |
| Breast Cancer | CD11c on myeloid DC | Inverse variance weighted | 28 | -0.034383639 | 0.015962963 | 0.031243031 | 0.966200761 | 0.936438859 | 0.996908556 |
| Breast Cancer | CD11c on myeloid DC | Simple mode | 28 | -0.02699986 | 0.046154502 | 0.563412662 | 0.973361378 | 0.889173691 | 1.065520023 |
| Breast Cancer | CD11c on myeloid DC | Weighted mode | 28 | -0.018475459 | 0.046676774 | 0.695350541 | 0.981694166 | 0.895868236 | 1.075742389 |
| Breast Cancer | CD45RA on naive CD4+ | MR Egger | 29 | -0.030864261 | 0.023731093 | 0.204393039 | 0.969607178 | 0.925540672 | 1.015771762 |
| Breast Cancer | CD45RA on naive CD4+ | Weighted median | 29 | -0.037802177 | 0.018724152 | 0.043497693 | 0.962903407 | 0.928206067 | 0.99889777 |
| Breast Cancer | CD45RA on naive CD4+ | Inverse variance weighted | 29 | -0.031188728 | 0.013104706 | 0.017314336 | 0.969292624 | 0.944713141 | 0.994511613 |
| Breast Cancer | CD45RA on naive CD4+ | Simple mode | 29 | -0.047989956 | 0.030844784 | 0.130974952 | 0.953143361 | 0.897227584 | 1.012543844 |
| Breast Cancer | CD45RA on naive CD4+ | Weighted mode | 29 | -0.041399719 | 0.021366077 | 0.062813223 | 0.959445545 | 0.920096034 | 1.000477906 |
| Breast Cancer | CD4RA on TD CD4+ | MR Egger | 24 | -0.059274358 | 0.029361112 | 0.055859089 | 0.942448165 | 0.889743431 | 0.998274911 |
| Breast Cancer | CD4RA on TD CD4+ | Weighted median | 24 | -0.044065898 | 0.020559724 | 0.032088072 | 0.956890898 | 0.919097603 | 0.996238254 |
| Breast Cancer | CD4RA on TD CD4+ | Inverse variance weighted | 24 | -0.029509872 | 0.013545755 | 0.029366397 | 0.970921293 | 0.945482828 | 0.997044186 |
| Breast Cancer | CD4RA on TD CD4+ | Simple mode | 24 | -0.048047282 | 0.034194954 | 0.173355369 | 0.953088723 | 0.891304292 | 1.019155995 |
| Breast Cancer | CD4RA on TD CD4+ | Weighted mode | 24 | -0.049852113 | 0.025995142 | 0.067643585 | 0.95137011 | 0.904111494 | 1.00109897 |
| Breast Cancer | HLA DR on CD33br HLA DR+ CD14- | MR Egger | 17 | -0.023155068 | 0.026324168 | 0.392943374 | 0.977110953 | 0.927975045 | 1.028848588 |
| Breast Cancer | HLA DR on CD33br HLA DR+ CD14- | Weighted median | 17 | -0.0254227 | 0.014537712 | 0.080335716 | 0.974897736 | 0.947511112 | 1.003075936 |
| Breast Cancer | HLA DR on CD33br HLA DR+ CD14- | Inverse variance weighted | 17 | -0.021827688 | 0.01058328 | 0.039163099 | 0.978408812 | 0.958322501 | 0.998916129 |
| Breast Cancer | HLA DR on CD33br HLA DR+ CD14- | Simple mode | 17 | -0.00916702 | 0.020783715 | 0.665066064 | 0.990874869 | 0.951321601 | 1.032072651 |
| Breast Cancer | HLA DR on CD33br HLA DR+ CD14- | Weighted mode | 17 | -0.023625782 | 0.015312707 | 0.14240364 | 0.976651121 | 0.947774486 | 1.006407565 |

| **Heterogeneity and pleiotropy in MR analyses** | | | | | | | | |  |
| --- | --- | --- | --- | --- | --- | --- | --- | --- | --- |
| Outcome | Exposure | Method | NO.of SNPs | Heterogeneity | | | Pleiotropy | | |
|  |  |  |  | Q | Q df | Q P-value | MR-egger regression | | |
|  |  |  |  |  |  |  | Egger intercept | Se | P-value |
| Breast Cancer | CD24+ CD27+ AC | MR Egger | 22 | 18.97615272 | 20 | 0.523376608 | 0.004997931 | 0.007571757 | 0.516735908 |
| Breast Cancer | CD24+ CD27+ AC | Inverse variance weighted | 22 | 19.41185225 | 21 | 0.558738382 |  |  |  |
| Breast Cancer | IgD- CD38br AC | MR Egger | 15 | 11.14870748 | 13 | 0.598361401 | 0.007860458 | 0.01015667 | 0.452822768 |
| Breast Cancer | IgD- CD38br AC | Inverse variance weighted | 15 | 11.74766087 | 14 | 0.626559481 |  |  |  |
| Breast Cancer | CD62L- myeloid DC AC | MR Egger | 15 | 10.46719158 | 13 | 0.655356604 | -0.010446944 | 0.0073351 | 0.177936804 |
| Breast Cancer | CD62L- myeloid DC AC | Inverse variance weighted | 15 | 12.49565148 | 14 | 0.566562677 |  |  |  |
| Breast Cancer | Activated & secreting Treg AC | MR Egger | 11 | 4.033884149 | 9 | 0.90916822 | -0.009597098 | 0.011161836 | 0.412208541 |
| Breast Cancer | Activated & secreting Treg AC | Inverse variance weighted | 11 | 4.773163474 | 10 | 0.905806635 |  |  |  |
| Breast Cancer | EM DN (CD4-CD8-) %T cell | MR Egger | 21 | 23.98926477 | 19 | 0.196564469 | 0.007582055 | 0.010999754 | 0.498971079 |
| Breast Cancer | EM DN (CD4-CD8-) %T cell | Inverse variance weighted | 21 | 24.58915432 | 20 | 0.21760314 |  |  |  |
| Breast Cancer | CD4+ AC | MR Egger | 20 | 27.22081273 | 18 | 0.074946009 | -0.003552547 | 0.014295025 | 0.806548245 |
| Breast Cancer | CD4+ AC | Inverse variance weighted | 20 | 27.31421089 | 19 | 0.097544468 |  |  |  |
| Breast Cancer | HLA DR+ CD4+ AC | MR Egger | 20 | 17.10958359 | 18 | 0.515583282 | 0.005985578 | 0.007925755 | 0.459893869 |
| Breast Cancer | HLA DR+ CD4+ AC | Inverse variance weighted | 20 | 17.67991972 | 19 | 0.543904828 |  |  |  |
| Breast Cancer | B cell %lymphocyte | MR Egger | 33 | 18.50548292 | 31 | 0.962667431 | -0.002487584 | 0.005115895 | 0.630212464 |
| Breast Cancer | B cell %lymphocyte | Inverse variance weighted | 33 | 18.74191823 | 32 | 0.969822825 |  |  |  |
| Breast Cancer | CD28+ CD45RA- CD8dim AC | MR Egger | 16 | 12.55957304 | 14 | 0.561457916 | -0.000781704 | 0.008052407 | 0.924041287 |
| Breast Cancer | CD28+ CD45RA- CD8dim AC | Inverse variance weighted | 16 | 12.56899698 | 15 | 0.635549692 |  |  |  |
| Breast Cancer | CD28- CD8br %CD8br | MR Egger | 24 | 13.52251065 | 22 | 0.91755701 | -0.008212524 | 0.007736091 | 0.299939166 |
| Breast Cancer | CD28- CD8br %CD8br | Inverse variance weighted | 24 | 14.64947508 | 23 | 0.906754936 |  |  |  |
| Breast Cancer | CD28- DN (CD4-CD8-) %T cell | MR Egger | 24 | 24.71905638 | 22 | 0.310697578 | 0.004800048 | 0.012508752 | 0.704858244 |
| Breast Cancer | CD28- DN (CD4-CD8-) %T cell | Inverse variance weighted | 24 | 24.88450852 | 23 | 0.356267521 |  |  |  |
| Breast Cancer | CD24 on IgD+ CD38br | MR Egger | 22 | 18.31510932 | 20 | 0.566658986 | 0.000736139 | 0.007099884 | 0.918453358 |
| Breast Cancer | CD24 on IgD+ CD38br | Inverse variance weighted | 22 | 18.32585954 | 21 | 0.628299927 |  |  |  |
| Breast Cancer | CD38 on IgD+ CD24- | MR Egger | 15 | 3.501055572 | 13 | 0.995434313 | -0.006259739 | 0.007337608 | 0.409045533 |
| Breast Cancer | CD38 on IgD+ CD24- | Inverse variance weighted | 15 | 4.228841204 | 14 | 0.993924697 |  |  |  |
| Breast Cancer | IgD on unsw mem | MR Egger | 20 | 15.57388733 | 18 | 0.622258656 | -0.009769437 | 0.008256444 | 0.252102827 |
| Breast Cancer | IgD on unsw mem | Inverse variance weighted | 20 | 16.97396784 | 19 | 0.591631517 |  |  |  |
| Breast Cancer | HVEM on EM CD4+ | MR Egger | 19 | 18.8210501 | 17 | 0.338899926 | -0.001254944 | 0.010125289 | 0.902815251 |
| Breast Cancer | HVEM on EM CD4+ | Inverse variance weighted | 19 | 18.83805714 | 18 | 0.40185899 |  |  |  |
| Breast Cancer | CD127 on CD45RA- CD4 not Treg | MR Egger | 15 | 14.50174296 | 13 | 0.339480815 | 0.007441514 | 0.011103101 | 0.514448012 |
| Breast Cancer | CD127 on CD45RA- CD4 not Treg | Inverse variance weighted | 15 | 15.00282742 | 14 | 0.377961442 |  |  |  |
| Breast Cancer | CD127 on CD28+ CD4+ | MR Egger | 18 | 15.12413358 | 16 | 0.51556625 | -0.003857428 | 0.007694753 | 0.622982435 |
| Breast Cancer | CD127 on CD28+ CD4+ | Inverse variance weighted | 18 | 15.37544158 | 17 | 0.56845866 |  |  |  |
| Breast Cancer | FSC-A on NK | MR Egger | 16 | 15.00458907 | 14 | 0.377841061 | -0.007506769 | 0.011438056 | 0.522274086 |
| Breast Cancer | FSC-A on NK | Inverse variance weighted | 16 | 15.46622305 | 15 | 0.418384456 |  |  |  |
| Breast Cancer | HLA DR on CD14+ CD16- monocyte | MR Egger | 19 | 19.21799327 | 17 | 0.31616402 | 0.00034488 | 0.007635992 | 0.964502049 |
| Breast Cancer | HLA DR on CD14+ CD16- monocyte | Inverse variance weighted | 19 | 19.2202993 | 18 | 0.378377095 |  |  |  |
| Breast Cancer | CD11c on myeloid DC | MR Egger | 28 | 18.53471448 | 26 | 0.855462078 | -0.002148707 | 0.007847887 | 0.78640613 |
| Breast Cancer | CD11c on myeloid DC | Inverse variance weighted | 28 | 18.60967779 | 27 | 0.883783477 |  |  |  |
| Breast Cancer | CD45RA on naive CD4+ | MR Egger | 29 | 26.86701275 | 27 | 0.470987497 | -0.000106489 | 0.006493252 | 0.987035923 |
| Breast Cancer | CD45RA on naive CD4+ | Inverse variance weighted | 29 | 26.86728171 | 28 | 0.525482982 |  |  |  |
| Breast Cancer | CD4RA on TD CD4+ | MR Egger | 24 | 21.1606497 | 22 | 0.510829459 | 0.00755786 | 0.006614596 | 0.265486237 |
| Breast Cancer | CD4RA on TD CD4+ | Inverse variance weighted | 24 | 22.46619211 | 23 | 0.492277592 |  |  |  |
| Breast Cancer | HLA DR on CD33br HLA DR+ CD14- | MR Egger | 17 | 9.774055667 | 15 | 0.833706553 | 0.000522835 | 0.009493825 | 0.956808661 |
| Breast Cancer | HLA DR on CD33br HLA DR+ CD14- | Inverse variance weighted | 17 | 9.777088491 | 16 | 0.878007033 |  |  |  |

| **Causal estimations of Gut microbiota and Mediator in MR methods** | | | | | | | | | |
| --- | --- | --- | --- | --- | --- | --- | --- | --- | --- |
| Mediator | Exposure | Method | NO.of SNPs | Beta | Se | Pvalue | or | or_lci95 | or_uci95 |
| CD11c on myeloid DC | Genus An7 | MR Egger | 15 | 0.982160761 | 0.402813616 | 0.029864808 | 2.670219719 | 1.212453597 | 5.88069792 |
| CD11c on myeloid DC | Genus An7 | Weighted median | 15 | 0.602675978 | 0.248680743 | 0.01537206 | 1.827001281 | 1.122167095 | 2.974542469 |
| CD11c on myeloid DC | Genus An7 | Inverse variance weighted | 15 | 0.470261692 | 0.186345519 | 0.011615977 | 1.600412953 | 1.110737794 | 2.305964229 |
| CD11c on myeloid DC | Genus An7 | Simple mode | 15 | 0.284950702 | 0.351491442 | 0.431109728 | 1.329696476 | 0.667662466 | 2.648183487 |
| CD11c on myeloid DC | Genus An7 | Weighted mode | 15 | 0.620844619 | 0.266791551 | 0.035478399 | 1.860498791 | 1.102889087 | 3.138534775 |
| CD127 on CD45RA- CD4 not Treg | Species Bacteroides A plebeius A | MR Egger | 20 | 0.116115417 | 0.163262453 | 0.486063559 | 1.123125492 | 0.815561055 | 1.546678651 |
| CD127 on CD45RA- CD4 not Treg | Species Bacteroides A plebeius A | Weighted median | 20 | 0.2862495 | 0.104554949 | 0.006185355 | 1.331424605 | 1.084719904 | 1.634238915 |
| CD127 on CD45RA- CD4 not Treg | Species Bacteroides A plebeius A | Inverse variance weighted | 20 | 0.225701544 | 0.072845849 | 0.00194605 | 1.253201584 | 1.086458887 | 1.445534875 |
| CD127 on CD45RA- CD4 not Treg | Species Bacteroides A plebeius A | Simple mode | 20 | 0.282635059 | 0.145083284 | 0.066331811 | 1.326620936 | 0.998273316 | 1.762967194 |
| CD127 on CD45RA- CD4 not Treg | Species Bacteroides A plebeius A | Weighted mode | 20 | 0.240410046 | 0.125577465 | 0.070746229 | 1.271770528 | 0.994294552 | 1.626681221 |
| CD127 on CD28+ CD4+ | Family Fibrobacteraceae | MR Egger | 16 | 1.305282188 | 1.456946627 | 0.385450502 | 3.688729861 | 0.212177265 | 64.12905747 |
| CD127 on CD28+ CD4+ | Family Fibrobacteraceae | Weighted median | 16 | 1.119034205 | 0.7514857 | 0.136462141 | 3.061895612 | 0.701960766 | 13.35573895 |
| CD127 on CD28+ CD4+ | Family Fibrobacteraceae | Inverse variance weighted | 16 | 1.219803489 | 0.565734013 | 0.031072493 | 3.38652218 | 1.117355602 | 10.26399515 |
| CD127 on CD28+ CD4+ | Family Fibrobacteraceae | Simple mode | 16 | 1.44572977 | 1.203723572 | 0.248355047 | 4.244948847 | 0.401090404 | 44.92650673 |
| CD127 on CD28+ CD4+ | Family Fibrobacteraceae | Weighted mode | 16 | 1.205745079 | 1.11305417 | 0.295792305 | 3.339246152 | 0.376875232 | 29.58688689 |
| CD38 on IgD+ CD24- | Species Megamonas funiformis | MR Egger | 37 | 0.144175437 | 0.137599803 | 0.301918279 | 1.155086736 | 0.882037968 | 1.512662059 |
| CD38 on IgD+ CD24- | Species Megamonas funiformis | Weighted median | 37 | 0.13153842 | 0.095114292 | 0.16667984 | 1.140581729 | 0.94659344 | 1.37432463 |
| CD38 on IgD+ CD24- | Species Megamonas funiformis | Inverse variance weighted | 37 | 0.170523698 | 0.07448256 | 0.022053531 | 1.185925756 | 1.024841412 | 1.372329301 |
| CD38 on IgD+ CD24- | Species Megamonas funiformis | Simple mode | 37 | 0.291383131 | 0.166003862 | 0.087718897 | 1.338277221 | 0.966586545 | 1.85289763 |
| CD38 on IgD+ CD24- | Species Megamonas funiformis | Weighted mode | 37 | 0.134705104 | 0.126347817 | 0.293456301 | 1.144199314 | 0.893207272 | 1.465720344 |
| HVEM on EM CD4+ | Species Megamonas funiformis | MR Egger | 38 | 0.313444147 | 0.199415354 | 0.124742102 | 1.368129047 | 0.925510366 | 2.02242693 |
| HVEM on EM CD4+ | Species Megamonas funiformis | Weighted median | 38 | 0.302925317 | 0.151352811 | 0.04534375 | 1.353813354 | 1.00629353 | 1.82134789 |
| HVEM on EM CD4+ | Species Megamonas funiformis | Inverse variance weighted | 38 | 0.243339006 | 0.109942638 | 0.026875111 | 1.275500953 | 1.028242913 | 1.582216284 |
| HVEM on EM CD4+ | Species Megamonas funiformis | Simple mode | 38 | 0.255264115 | 0.253126207 | 0.31979014 | 1.290802496 | 0.785949097 | 2.119947831 |
| HVEM on EM CD4+ | Species Megamonas funiformis | Weighted mode | 38 | 0.356147474 | 0.218030267 | 0.110850755 | 1.427818099 | 0.931283209 | 2.189091894 |
| HLA DR+ CD4+ AC | Species Prevotella bivia | MR Egger | 14 | 0.09936343 | 0.226610114 | 0.668830094 | 1.104467623 | 0.7083674 | 1.722056564 |
| HLA DR+ CD4+ AC | Species Prevotella bivia | Weighted median | 14 | 0.130826592 | 0.163469763 | 0.423530836 | 1.139770119 | 0.827311375 | 1.570238199 |
| HLA DR+ CD4+ AC | Species Prevotella bivia | Inverse variance weighted | 14 | 0.24071388 | 0.110641728 | 0.029584009 | 1.272156994 | 1.024142926 | 1.580231992 |
| HLA DR+ CD4+ AC | Species Prevotella bivia | Simple mode | 14 | 0.139872936 | 0.243905533 | 0.576112116 | 1.150127649 | 0.713065563 | 1.855079923 |
| HLA DR+ CD4+ AC | Species Prevotella bivia | Weighted mode | 14 | 0.072762476 | 0.248619334 | 0.77439634 | 1.075475055 | 0.660649776 | 1.750771188 |
| HLA DR on CD33br HLA DR+ CD14- | Species Prevotellamassilia | MR Egger | 13 | -0.188962538 | 0.254247168 | 0.472921553 | 0.827817518 | 0.502938698 | 1.362555406 |
| HLA DR on CD33br HLA DR+ CD14- | Species Prevotellamassilia | Weighted median | 13 | -0.288650349 | 0.14564035 | 0.047486022 | 0.749274144 | 0.56320847 | 0.996809836 |
| HLA DR on CD33br HLA DR+ CD14- | Species Prevotellamassilia | Inverse variance weighted | 13 | -0.259954327 | 0.111625412 | 0.019869156 | 0.771086803 | 0.619563468 | 0.959667393 |
| HLA DR on CD33br HLA DR+ CD14- | Species Prevotellamassilia | Simple mode | 13 | -0.358431998 | 0.204929492 | 0.105789023 | 0.698771142 | 0.467622561 | 1.044177826 |
| HLA DR on CD33br HLA DR+ CD14- | Species Prevotellamassilia | Weighted mode | 13 | -0.091710124 | 0.172231594 | 0.604112707 | 0.912369585 | 0.650974994 | 1.278725399 |

| **Table S8.** Sensitivity analysis of Gut microbiota and Mediator | | | | | | | | | | |
| --- | --- | --- | --- | --- | --- | --- | --- | --- | --- | --- |
|  |  |  |  |  |  |  |  |  |  |  |
| **Heterogeneity and pleiotropy in MR analyses** | | | | | | | | |  |  |
| Mediator | Exposure | Method | NO.of SNPs | Heterogeneity | | | Pleiotropy | | | |
|  |  |  |  | Q | Q df | Q P-value | Mr-egger regression | | | Mr-presso |
|  |  |  |  |  |  |  | Egger intercept | Se | P-value | Global test P-value |
| CD11c on myeloid DC | Genus An7 | MR Egger | 15 | 7.840574141 | 13 | 0.853829153 | -0.042013901 | 0.0293 | 0.175351 | 0.806 |
| CD11c on myeloid DC | Genus An7 | Inverse variance weighted | 15 | 9.895244536 | 14 | 0.769801468 |  |  |  |  |
| CD127 on CD45RA- CD4 not Treg | Species Bacteroides A plebeius A | MR Egger | 20 | 17.47619077 | 18 | 0.49062664 | 0.019389277 | 0.0259 | 0.462931 | 0.523 |
| CD127 on CD45RA- CD4 not Treg | Species Bacteroides A plebeius A | Inverse variance weighted | 20 | 18.03872857 | 19 | 0.519852664 |  |  |  |  |
| CD127 on CD28+ CD4+ | Family Fibrobacteraceae | MR Egger | 16 | 6.105080222 | 14 | 0.963772975 | -0.001662115 | 0.0261 | 0.950137 | 0.975 |
| CD127 on CD28+ CD4+ | Family Fibrobacteraceae | Inverse variance weighted | 16 | 6.109133507 | 15 | 0.977855662 |  |  |  |  |
| CD38 on IgD+ CD24- | Species Megamonas funiformis | MR Egger | 37 | 44.30798508 | 35 | 0.134579742 | 0.003534926 | 0.0154 | 0.820187 | 0.188 |
| CD38 on IgD+ CD24- | Species Megamonas funiformis | Inverse variance weighted | 37 | 44.37438358 | 36 | 0.159476109 |  |  |  |  |
| HVEM on EM CD4+ | Species Megamonas funiformis | MR Egger | 38 | 24.23411284 | 36 | 0.932437595 | -0.009007132 | 0.0214 | 0.675983 | 0.944 |
| HVEM on EM CD4+ | Species Megamonas funiformis | Inverse variance weighted | 38 | 24.41167392 | 37 | 0.944395075 |  |  |  |  |
| HLA DR+ CD4+ AC | Species Prevotella bivia | MR Egger | 14 | 9.062858367 | 12 | 0.697552319 | 0.014969289 | 0.0209 | 0.48844 | 0.627 |
| HLA DR+ CD4+ AC | Species Prevotella bivia | Inverse variance weighted | 14 | 9.573716661 | 13 | 0.728365923 |  |  |  |  |
| HLA DR on CD33br HLA DR+ CD14- | Species Prevotellamassilia | MR Egger | 13 | 4.786583977 | 11 | 0.941059787 | -0.012283736 | 0.0395 | 0.761779 | 0.972 |
| HLA DR on CD33br HLA DR+ CD14- | Species Prevotellamassilia | Inverse variance weighted | 13 | 4.883166954 | 12 | 0.96176773 |  |  |  |  |

| **Table S9.** The leave-one-out sensitivity analysis of the MR analysis of 18 genera on Breast cancer | | | | | | | | | | |  |  |  |
| --- | --- | --- | --- | --- | --- | --- | --- | --- | --- | --- | --- | --- | --- |
| 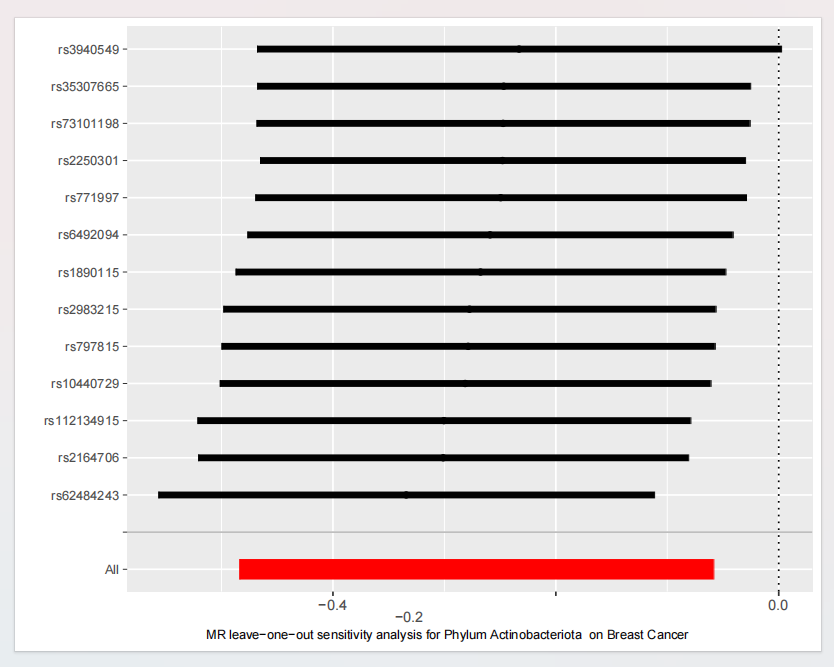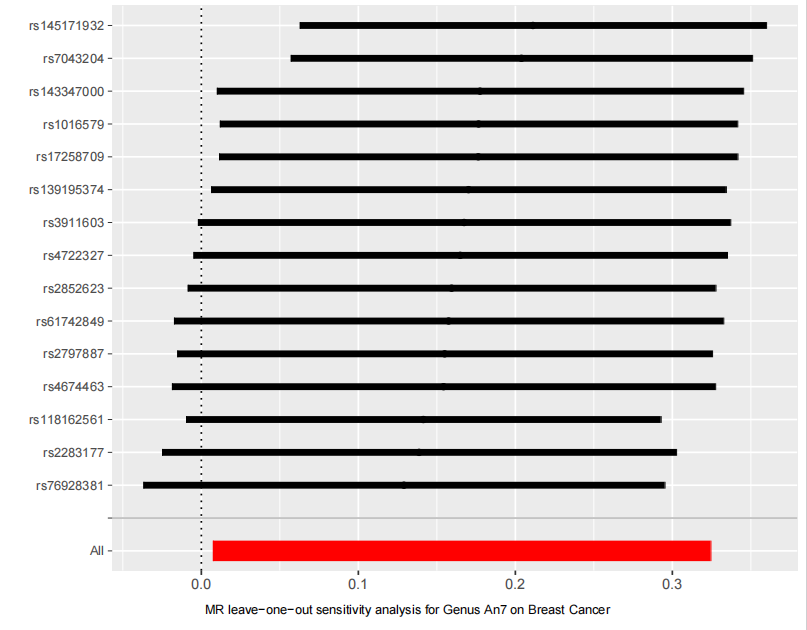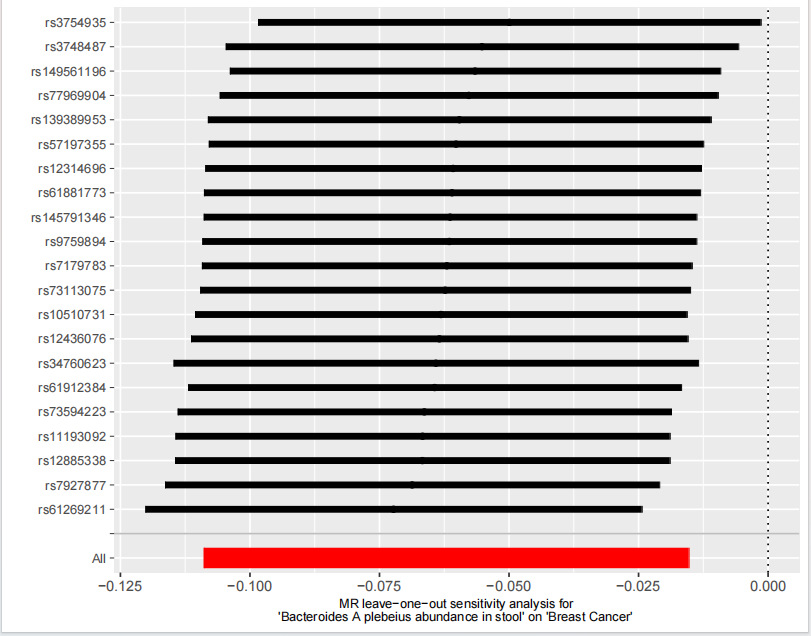   \|  \| \| --- \| |  |  |  |  |  |  |  |  |  |  |  |  |  |
|  |  |  |  |  |  |  |  |  |  |  |  |  |  |
|  |  |  |  |  |  |  |  |  |  |  |  |  |  |
|  |  |  |  |  |  |  |  |  |  |  |  |  |  |
|  |  |  |  |  |  |  |  |  |  |  |  |  |  |
|  |  |  |  |  |  |  |  |  |  |  |  |  |  |
|  |  |  |  |  |  |  |  |  |  |  |  |  |  |
|  |  |  |  |  |  |  |  |  |  |  |  |  |  |
|  |  |  |  |  |  |  |  |  |  |  |  |  |  |
|  |  |  |  |  |  |  |  |  |  |  |  |  |  |
|  |  |  |  |  |  |  |  |  |  |  |  |  |  |
|  |  |  |  |  |  |  |  |  |  |  |  |  |  |
|  |  |  |  |  |  |  |  |  |  |  |  |  |  |
|  |  |  |  |  |  |  |  |  |  |  |  |  |  |
|  |  |  |  |  |  |  |  |  |  |  |  |  |  |
|  |  |  |  |  |  |  |  |  |  |  |  |  |  |
|  |  |  |  |  |  |  |  |  |  |  |  |  |  |
|  |  |  |  |  |  |  |  |  |  |  |  |  |  |
|  |  |  |  |  |  |  |  |  |  |  |  |  |  |
|  |  |  |  |  |  |  |  |  |  |  |  |  |  |
| 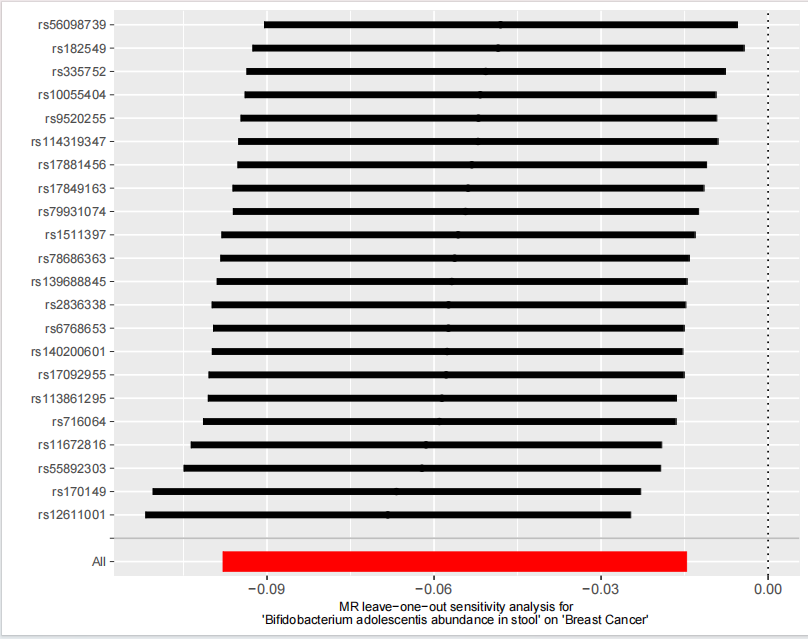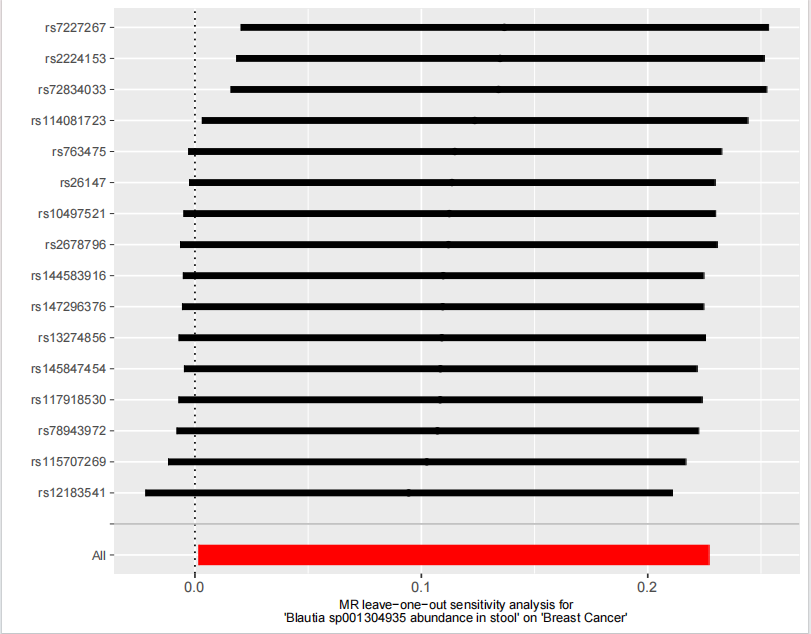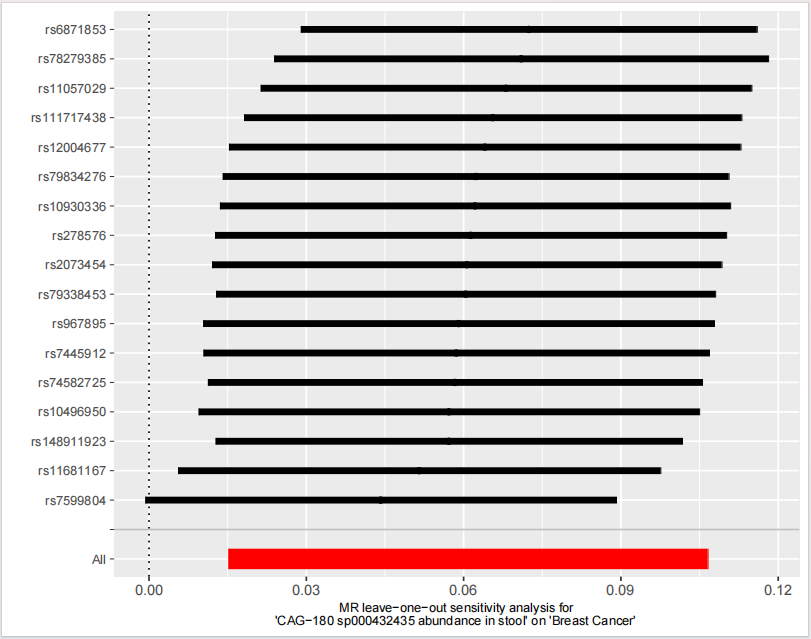   \|  \| \| --- \| |  |  |  |  |  |  |  |  |  |  |  |  |  |
|  |  |  |  |  |  |  |  |  |  |  |  |  |  |
|  |  |  |  |  |  |  |  |  |  |  |  |  |  |
|  |  |  |  |  |  |  |  |  |  |  |  |  |  |
|  |  |  |  |  |  |  |  |  |  |  |  |  |  |
|  |  |  |  |  |  |  |  |  |  |  |  |  |  |
|  |  |  |  |  |  |  |  |  |  |  |  |  |  |
|  |  |  |  |  |  |  |  |  |  |  |  |  |  |
|  |  |  |  |  |  |  |  |  |  |  |  |  |  |
|  |  |  |  |  |  |  |  |  |  |  |  |  |  |
|  |  |  |  |  |  |  |  |  |  |  |  |  |  |
|  |  |  |  |  |  |  |  |  |  |  |  |  |  |
|  |  |  |  |  |  |  |  |  |  |  |  |  |  |
|  |  |  |  |  |  |  |  |  |  |  |  |  |  |
|  |  |  |  |  |  |  |  |  |  |  |  |  |  |
|  |  |  |  |  |  |  |  |  |  |  |  |  |  |
|  |  |  |  |  |  |  |  |  |  |  |  |  |  |
|  |  |  |  |  |  |  |  |  |  |  |  |  |  |
|  |  |  |  |  |  |  |  |  |  |  |  |  |  |
| 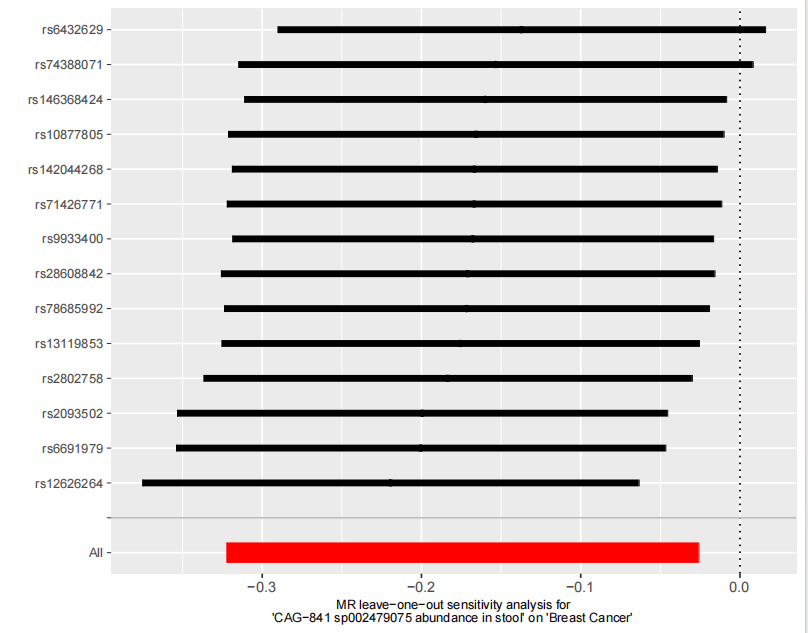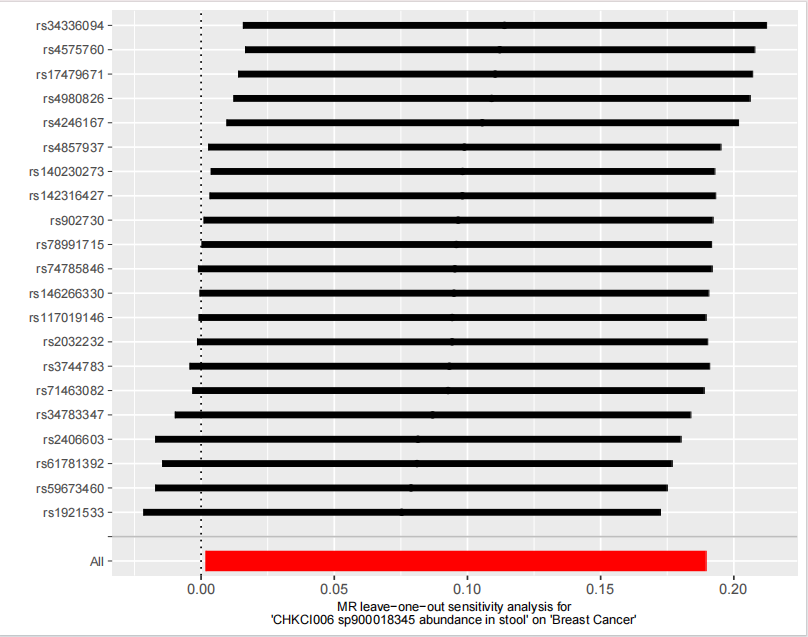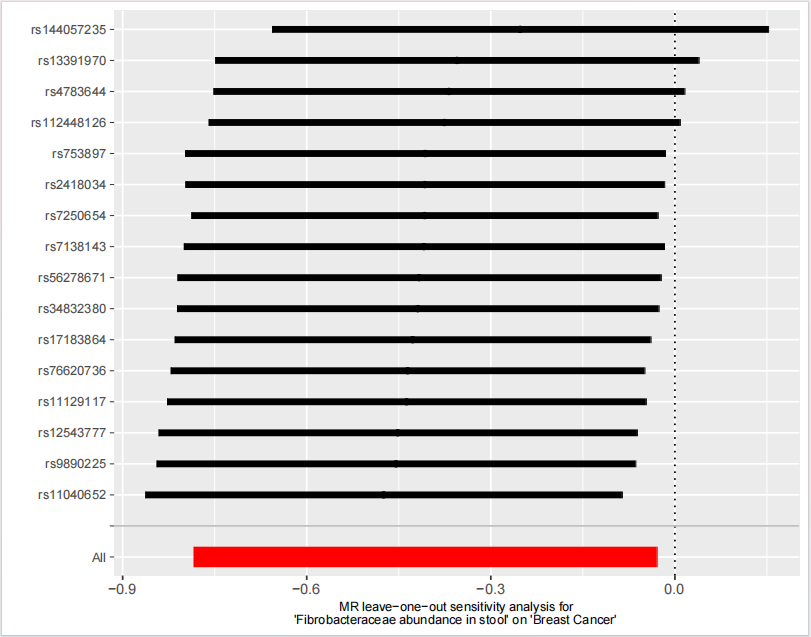   \|  \| \| --- \| |  |  |  |  |  |  |  |  |  |  |  |  |  |
|  |  |  |  |  |  |  |  |  |  |  |  |  |  |
|  |  |  |  |  |  |  |  |  |  |  |  |  |  |
|  |  |  |  |  |  |  |  |  |  |  |  |  |  |
|  |  |  |  |  |  |  |  |  |  |  |  |  |  |
|  |  |  |  |  |  |  |  |  |  |  |  |  |  |
|  |  |  |  |  |  |  |  |  |  |  |  |  |  |
|  |  |  |  |  |  |  |  |  |  |  |  |  |  |
|  |  |  |  |  |  |  |  |  |  |  |  |  |  |
|  |  |  |  |  |  |  |  |  |  |  |  |  |  |
|  |  |  |  |  |  |  |  |  |  |  |  |  |  |
|  |  |  |  |  |  |  |  |  |  |  |  |  |  |
|  |  |  |  |  |  |  |  |  |  |  |  |  |  |
|  |  |  |  |  |  |  |  |  |  |  |  |  |  |
|  |  |  |  |  |  |  |  |  |  |  |  |  |  |
|  |  |  |  |  |  |  |  |  |  |  |  |  |  |
|  |  |  |  |  |  |  |  |  |  |  |  |  |  |
|  |  |  |  |  |  |  |  |  |  |  |  |  |  |
|  |  |  |  |  |  |  |  |  |  |  |  |  |  |
| 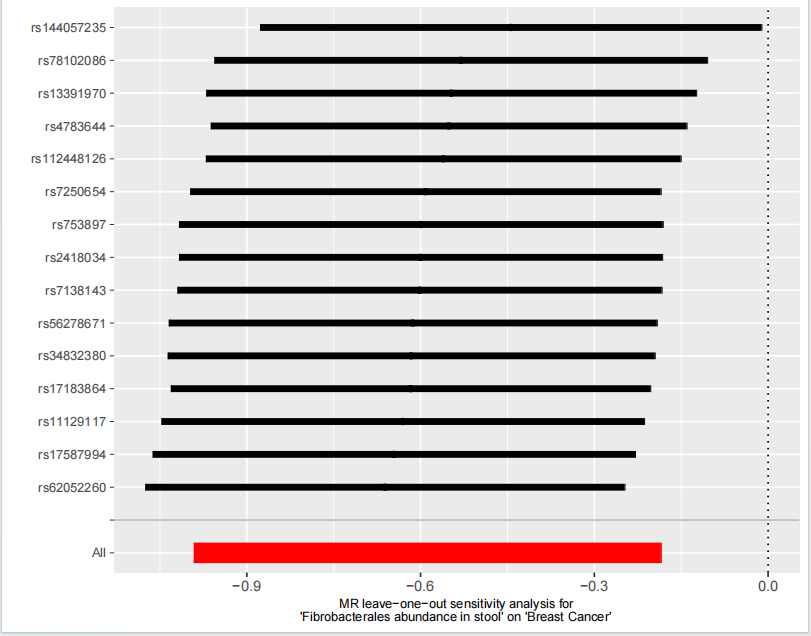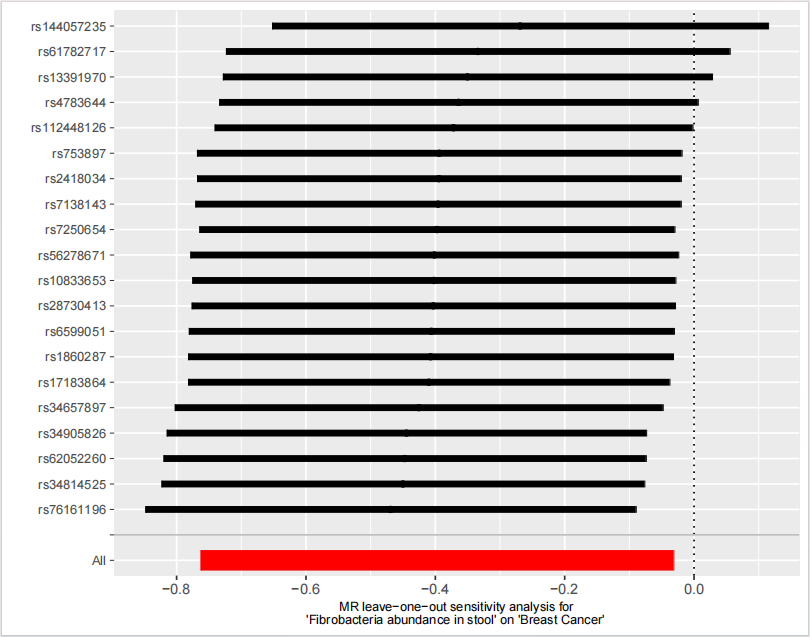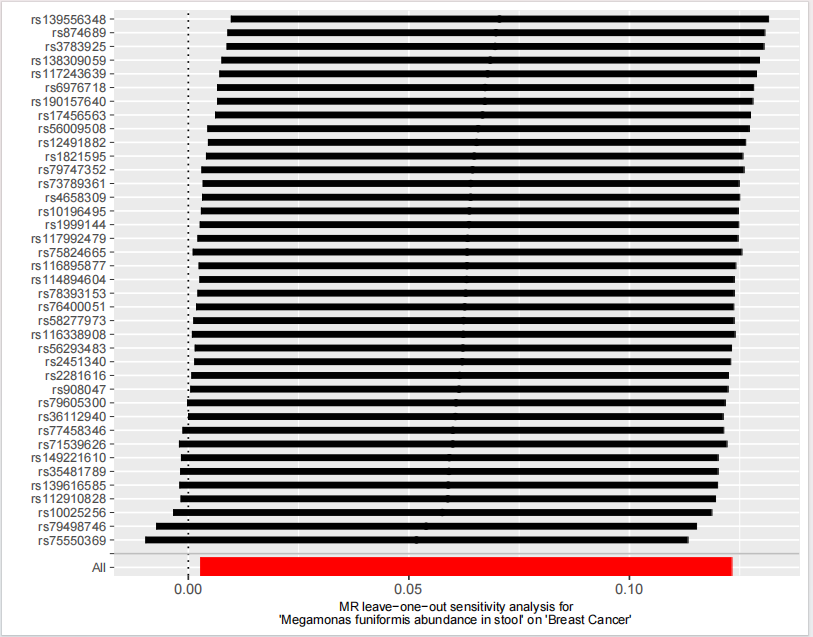   \|  \| \| --- \| |  |  |  |  |  |  |  |  |  |  |  |  |  |
|  |  |  |  |  |  |  |  |  |  |  |  |  |  |
|  |  |  |  |  |  |  |  |  |  |  |  |  |  |
|  |  |  |  |  |  |  |  |  |  |  |  |  |  |
|  |  |  |  |  |  |  |  |  |  |  |  |  |  |
|  |  |  |  |  |  |  |  |  |  |  |  |  |  |
|  |  |  |  |  |  |  |  |  |  |  |  |  |  |
|  |  |  |  |  |  |  |  |  |  |  |  |  |  |
|  |  |  |  |  |  |  |  |  |  |  |  |  |  |
|  |  |  |  |  |  |  |  |  |  |  |  |  |  |
|  |  |  |  |  |  |  |  |  |  |  |  |  |  |
|  |  |  |  |  |  |  |  |  |  |  |  |  |  |
|  |  |  |  |  |  |  |  |  |  |  |  |  |  |
|  |  |  |  |  |  |  |  |  |  |  |  |  |  |
|  |  |  |  |  |  |  |  |  |  |  |  |  |  |
|  |  |  |  |  |  |  |  |  |  |  |  |  |  |
|  |  |  |  |  |  |  |  |  |  |  |  |  |  |
|  |  |  |  |  |  |  |  |  |  |  |  |  |  |
|  |  |  |  |  |  |  |  |  |  |  |  |  |  |
| 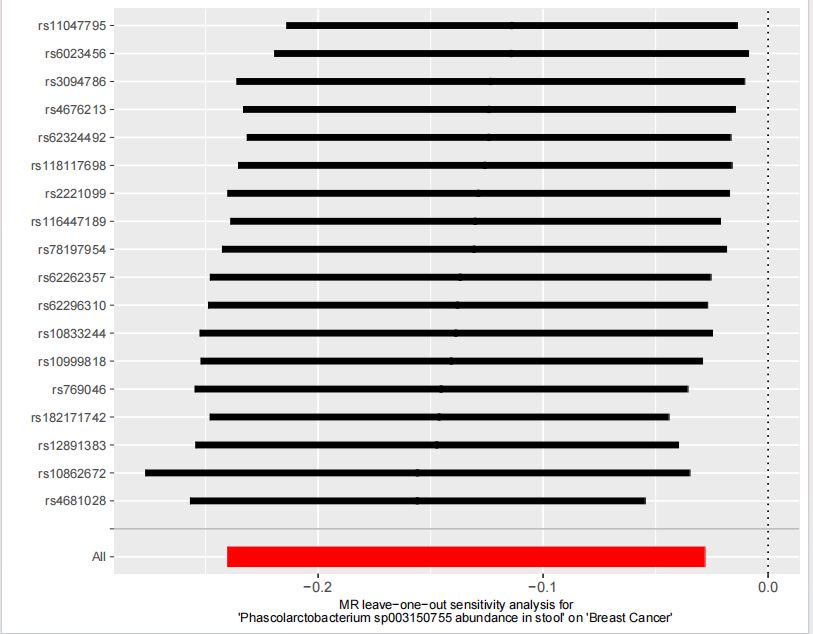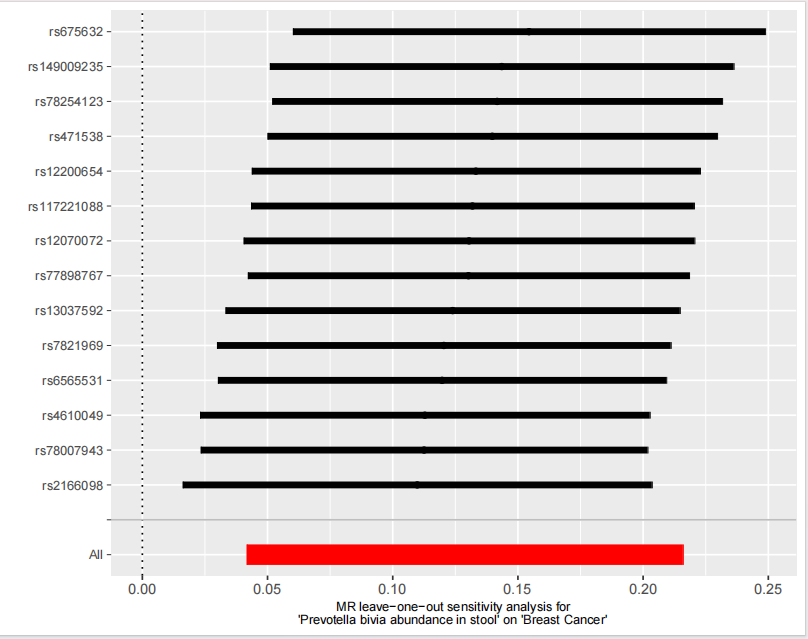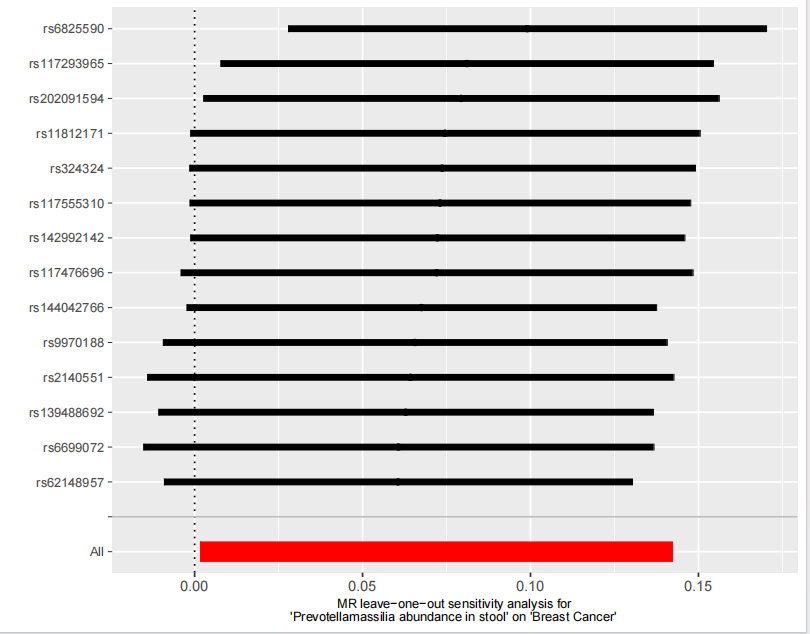   \|  \| \| --- \| |  |  |  |  |  |  |  |  |  |  |  |  |  |
|  |  |  |  |  |  |  |  |  |  |  |  |  |  |
|  |  |  |  |  |  |  |  |  |  |  |  |  |  |
|  |  |  |  |  |  |  |  |  |  |  |  |  |  |
|  |  |  |  |  |  |  |  |  |  |  |  |  |  |
|  |  |  |  |  |  |  |  |  |  |  |  |  |  |
|  |  |  |  |  |  |  |  |  |  |  |  |  |  |
|  |  |  |  |  |  |  |  |  |  |  |  |  |  |
|  |  |  |  |  |  |  |  |  |  |  |  |  |  |
|  |  |  |  |  |  |  |  |  |  |  |  |  |  |
|  |  |  |  |  |  |  |  |  |  |  |  |  |  |
|  |  |  |  |  |  |  |  |  |  |  |  |  |  |
|  |  |  |  |  |  |  |  |  |  |  |  |  |  |
|  |  |  |  |  |  |  |  |  |  |  |  |  |  |
|  |  |  |  |  |  |  |  |  |  |  |  |  |  |
|  |  |  |  |  |  |  |  |  |  |  |  |  |  |
|  |  |  |  |  |  |  |  |  |  |  |  |  |  |
|  |  |  |  |  |  |  |  |  |  |  |  |  |  |
|  |  |  |  |  |  |  |  |  |  |  |  |  |  |
| 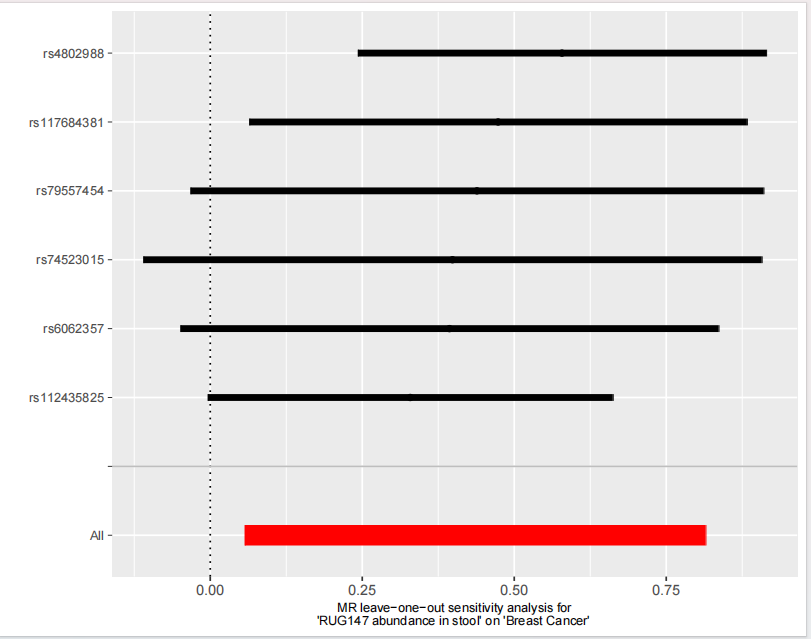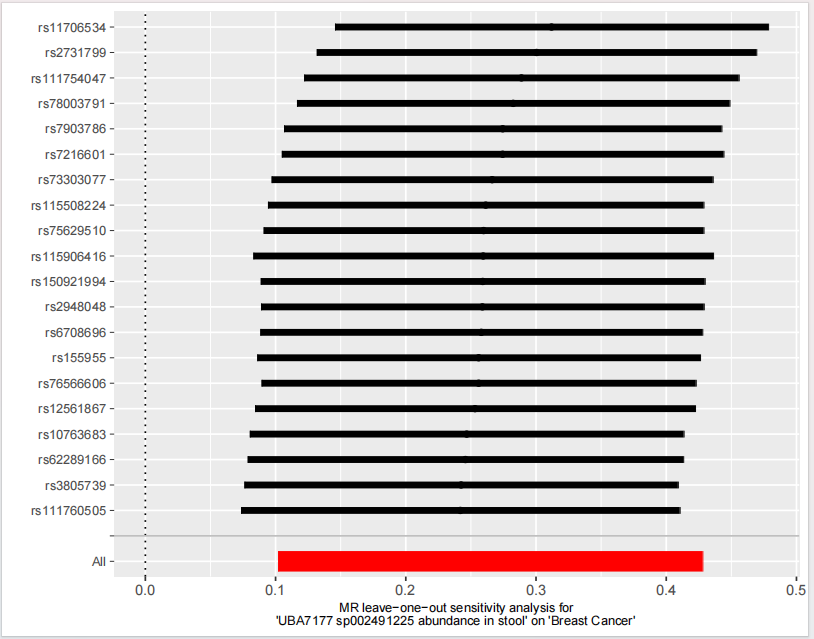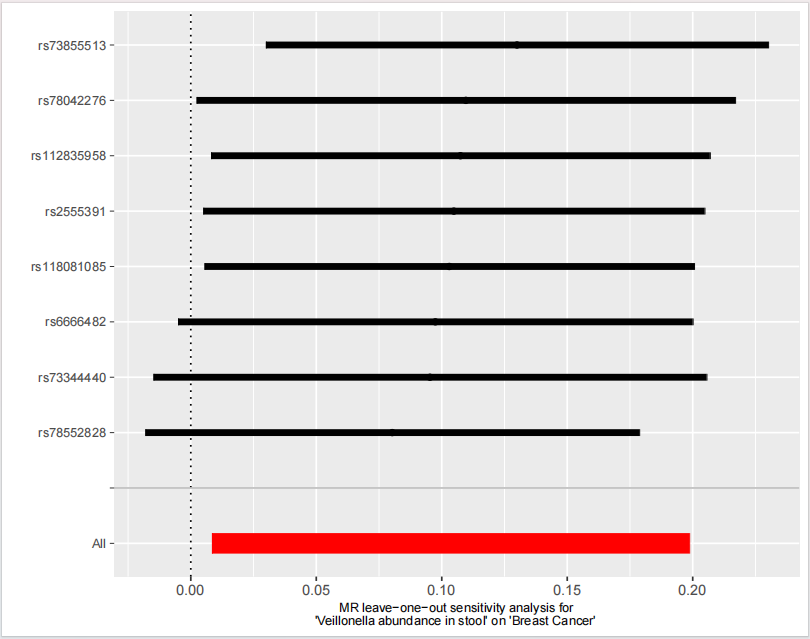   \|  \| \| --- \| |  |  |  |  |  |  |  |  |  |  |  |  |  |
|  |  |  |  |  |  |  |  |  |  |  |  |  |  |
|  |  |  |  |  |  |  |  |  |  |  |  |  |  |
|  |  |  |  |  |  |  |  |  |  |  |  |  |  |
|  |  |  |  |  |  |  |  |  |  |  |  |  |  |
|  |  |  |  |  |  |  |  |  |  |  |  |  |  |
|  |  |  |  |  |  |  |  |  |  |  |  |  |  |
|  |  |  |  |  |  |  |  |  |  |  |  |  |  |
|  |  |  |  |  |  |  |  |  |  |  |  |  |  |
|  |  |  |  |  |  |  |  |  |  |  |  |  |  |
|  |  |  |  |  |  |  |  |  |  |  |  |  |  |
|  |  |  |  |  |  |  |  |  |  |  |  |  |  |
|  |  |  |  |  |  |  |  |  |  |  |  |  |  |
|  |  |  |  |  |  |  |  |  |  |  |  |  |  |
|  |  |  |  |  |  |  |  |  |  |  |  |  |  |
|  |  |  |  |  |  |  |  |  |  |  |  |  |  |
|  |  |  |  |  |  |  |  |  |  |  |  |  |  |
|  |  |  |  |  |  |  |  |  |  |  |  |  |  |
|  |  |  |  |  |  |  |  |  |  |  |  |  |  |
|  |  |  |  |  |  |  |  |  |  |  |  |  |  |
| Bacteroides A plebeius abundance in stool：Species Bacteroides A plebeius A | | | | | | | |  |  |  |  |  |  |
| Bifidobacterium adolescentis abundance in stool：Species Bifidobacterium adolescentis | | | | | | | | |  |  |  |  |  |
| Blautia sp001304935 abundance in stool：Species Blautia sp001304935 | | | | | | |  |  |  |  |  |  |  |
| CAG-180 sp000432435 abundance in stool：Species CAG-180 sp000432435 | | | | | | | |  |  |  |  |  |  |
| CAG-841 sp002479075 abundance in stool：Species CAG-841 sp002479075 | | | | | | | |  |  |  |  |  |  |
| CHKCI006 sp900018345 abundance in stool：Species CHKCI006 sp900018345 | | | | | | | |  |  |  |  |  |  |
| Fibrobacteraceae abundance in stool：Family Fibrobacteraceae | | | | | | |  |  |  |  |  |  |  |
| Fibrobacterales abundance in stool：Order Fibrobacterales | | | | | |  |  |  |  |  |  |  |  |
| Fibrobacteria abundance in stool：Class Fibrobacteria | | | | | |  |  |  |  |  |  |  |  |
| Megamonas funiformis abundance in stool：Species Megamonas funiformis | | | | | | | |  |  |  |  |  |  |
| Phascolarctobacterium sp003150755 abundance in stool：Species Phascolarctobacterium sp003150755 | | | | | | | | | |  |  |  |  |
| Prevotella bivia abundance in stool：Species Prevotella bivia | | | | | |  |  |  |  |  |  |  |  |
| Prevotellamassilia abundance in stool：Species Prevotellamassilia | | | | | | |  |  |  |  |  |  |  |
| RUG147 abundance in stool：Genus RUG147 | | | | |  |  |  |  |  |  |  |  |  |
| UBA7177 sp002491225 abundance in stool：Species UBA7177 sp002491225 | | | | | | | |  |  |  |  |  |  |
| Veillonella abundance in stool:Genus Veillonella | | | | |  |  |  |  |  |  |  |  |  |

OA_Supplemental Digital Content S10


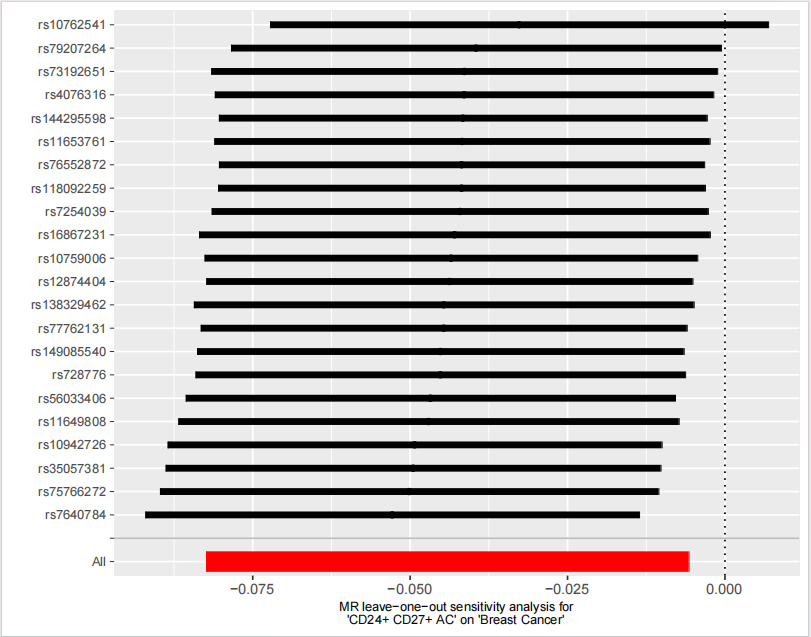

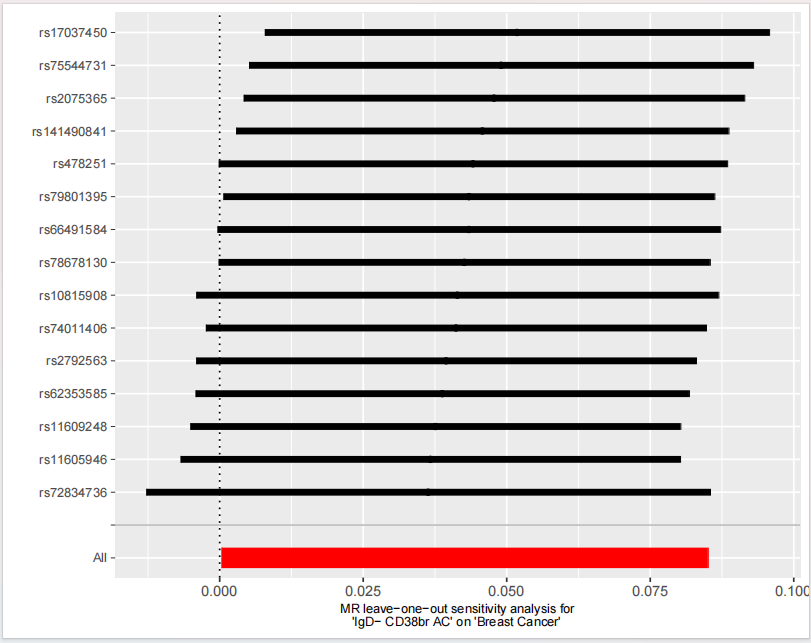

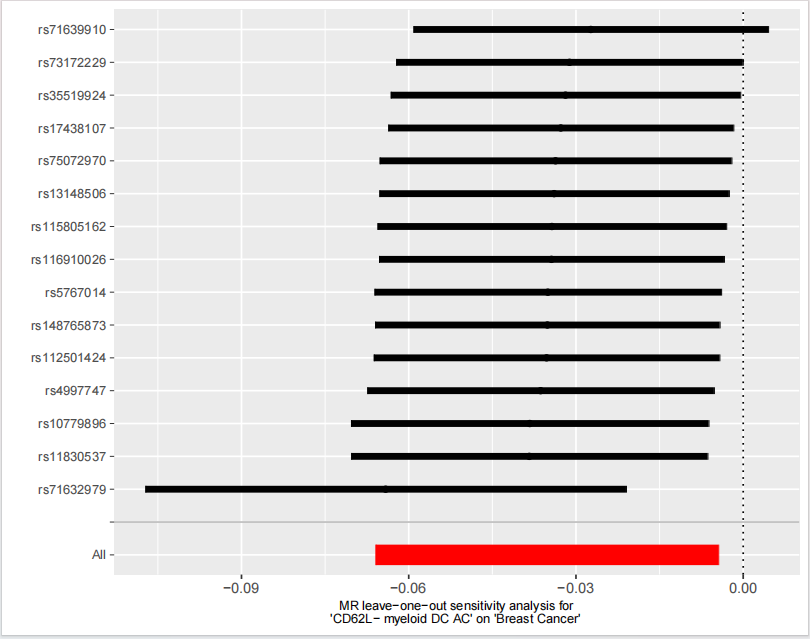

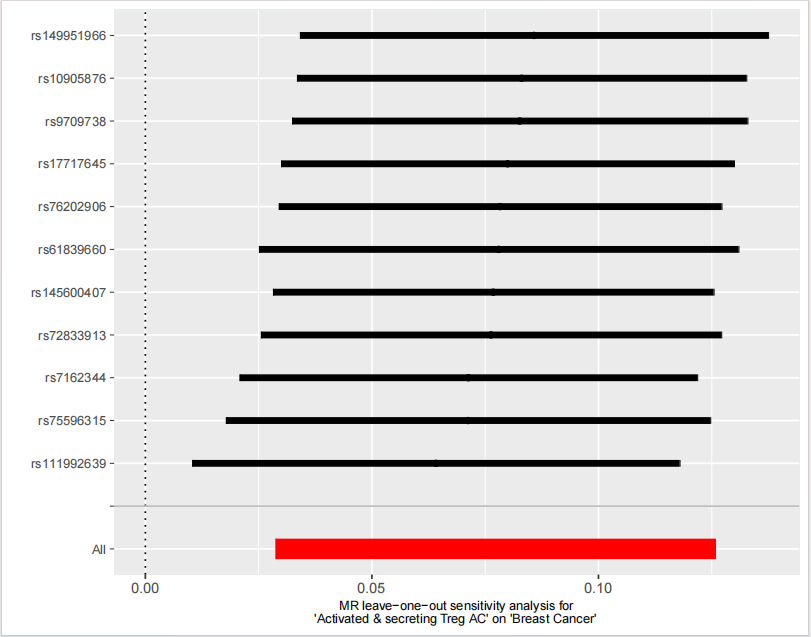

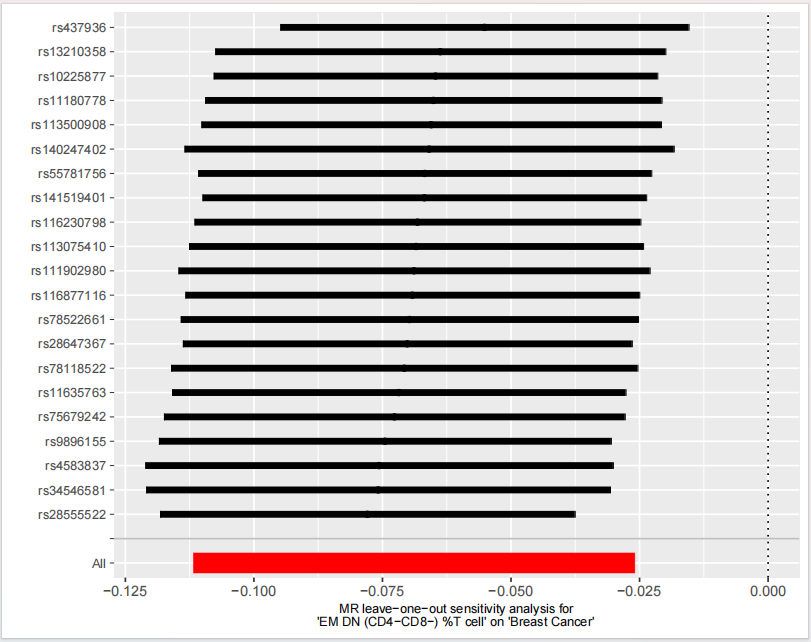

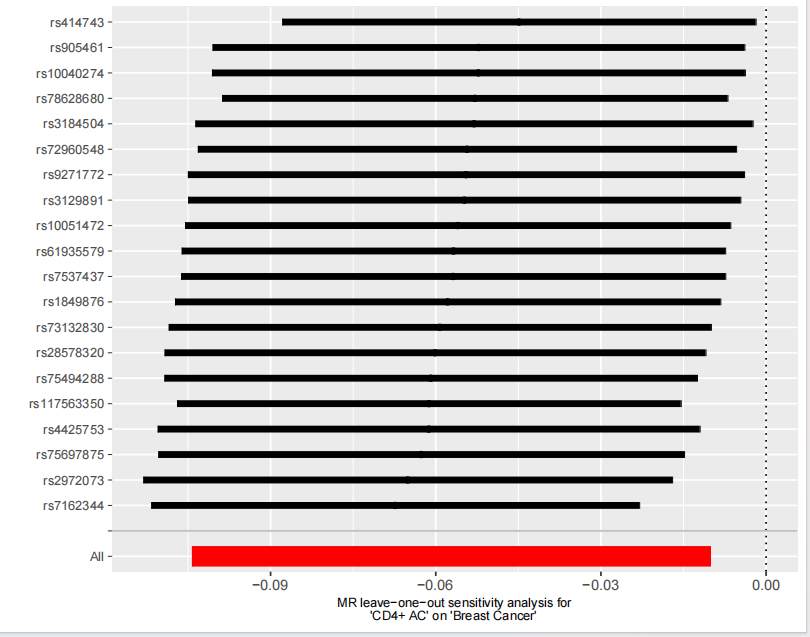

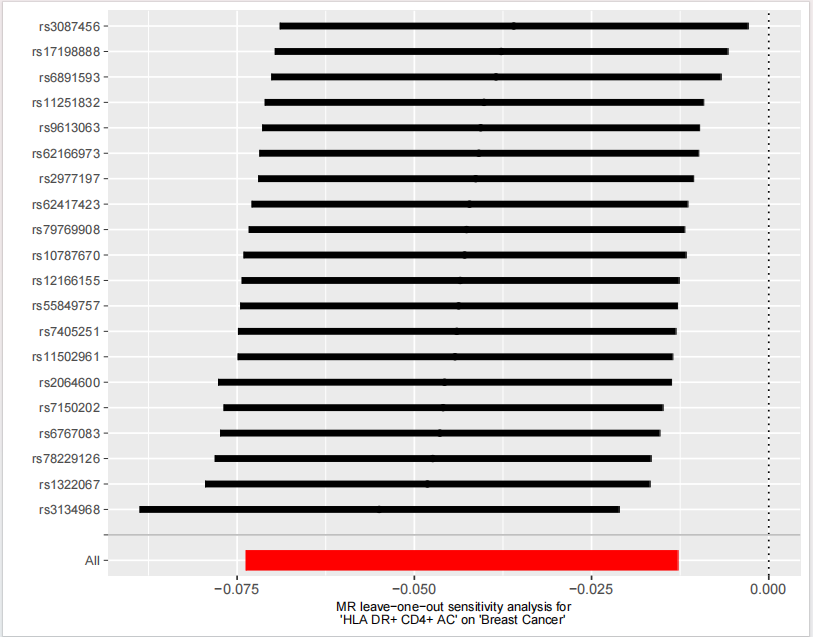

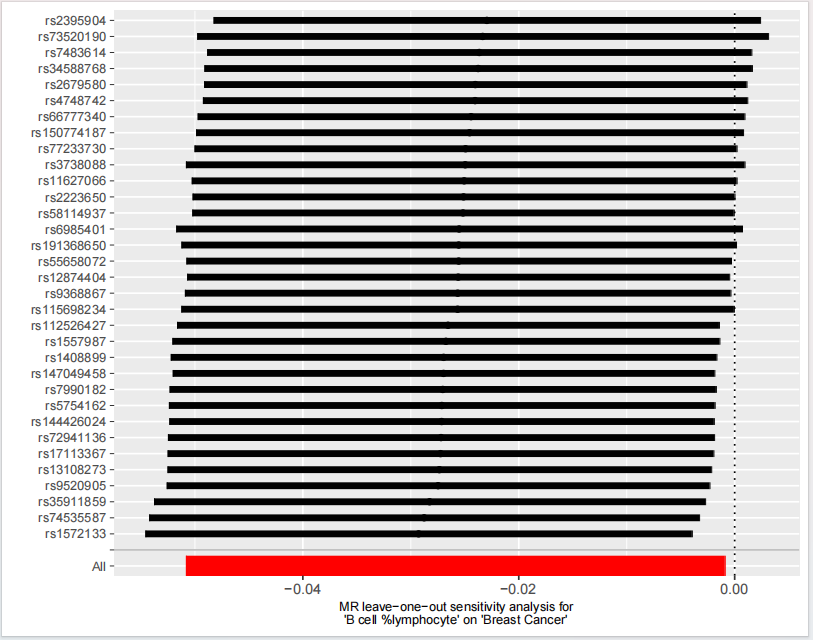

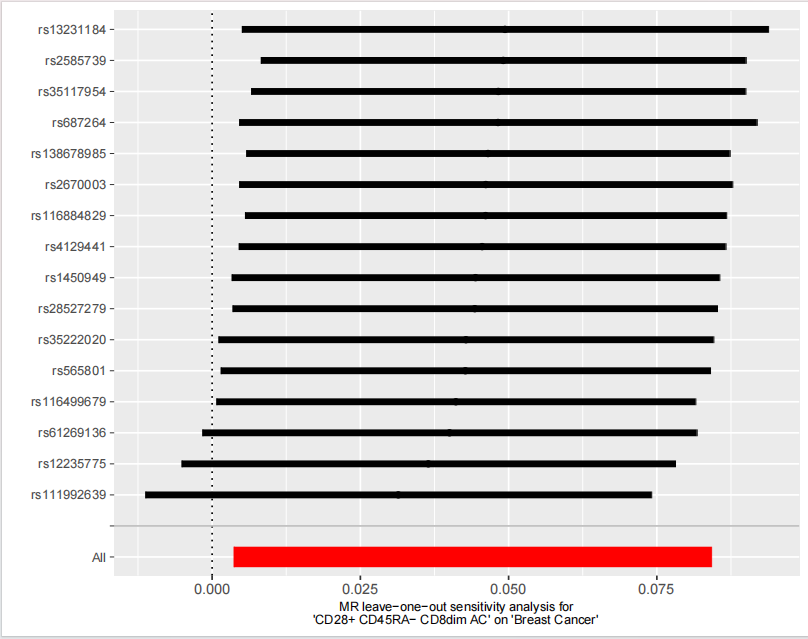

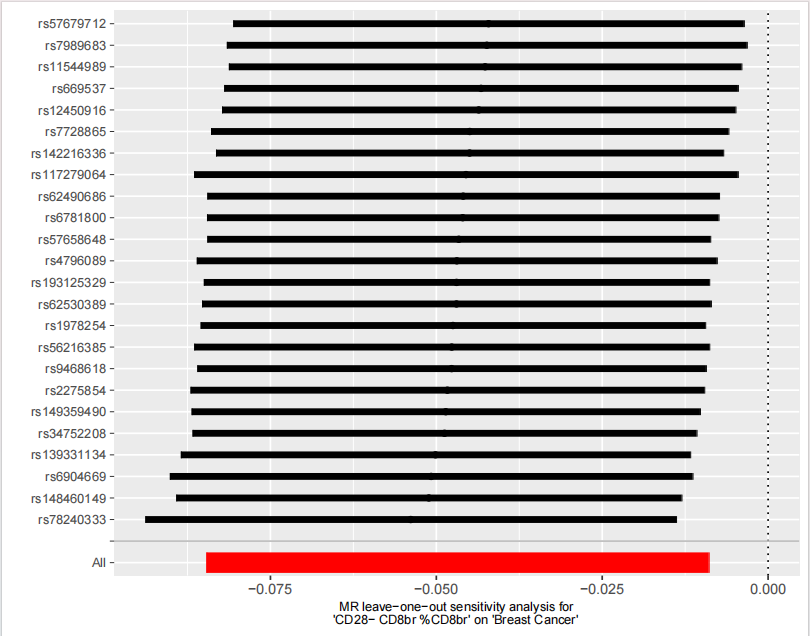

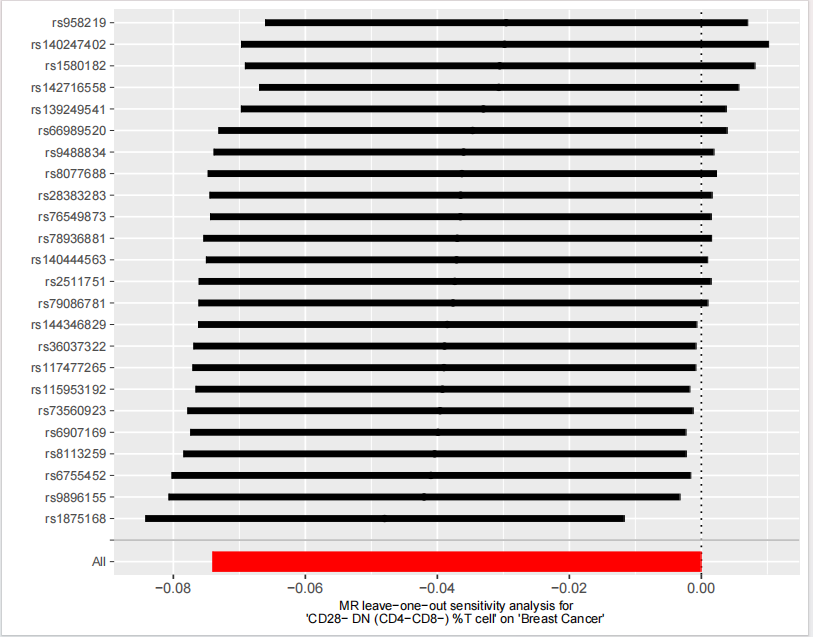

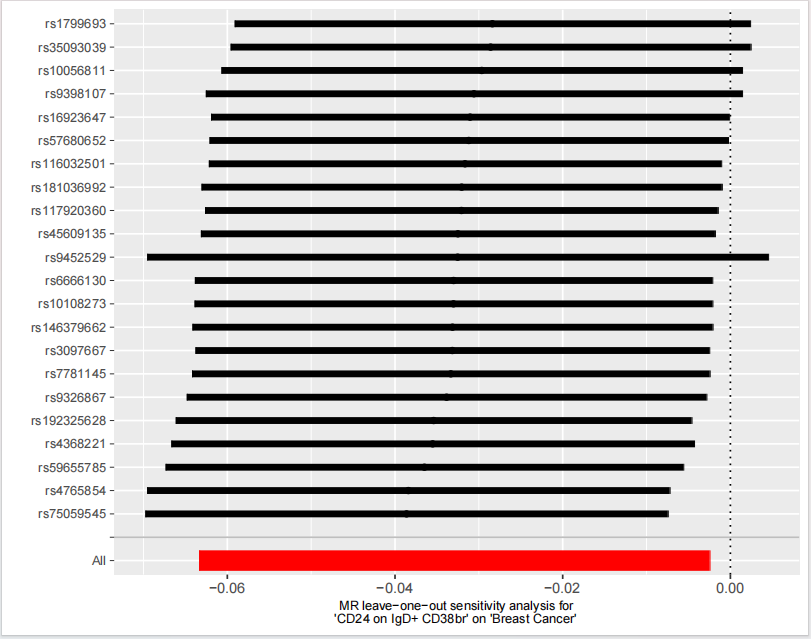

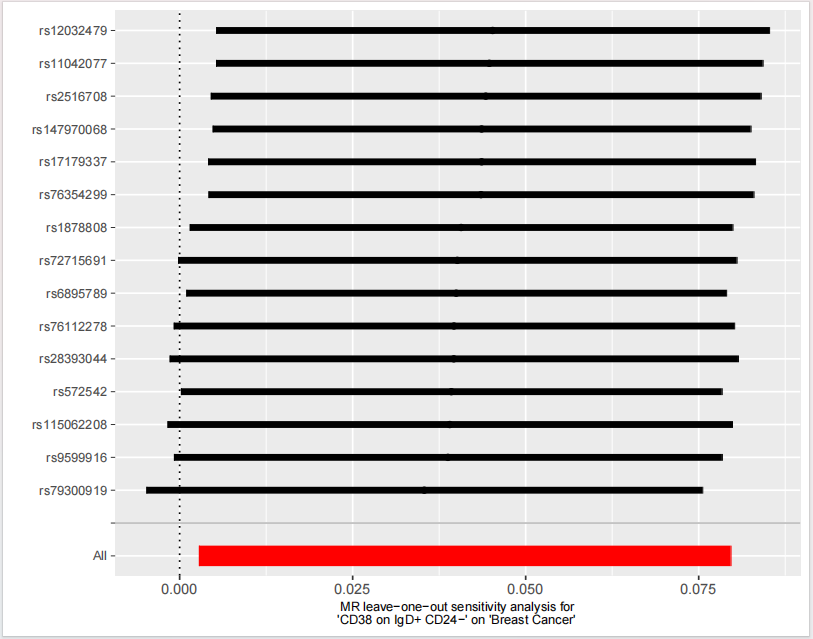

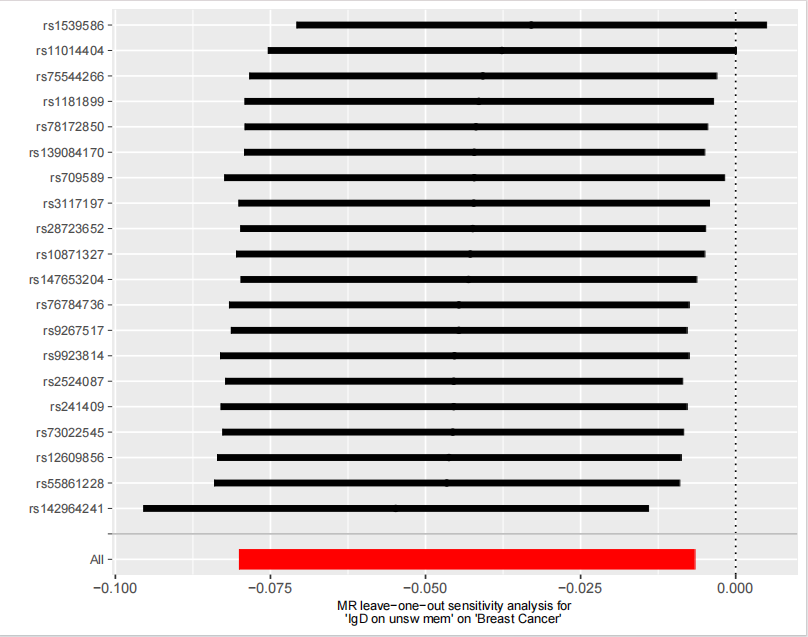

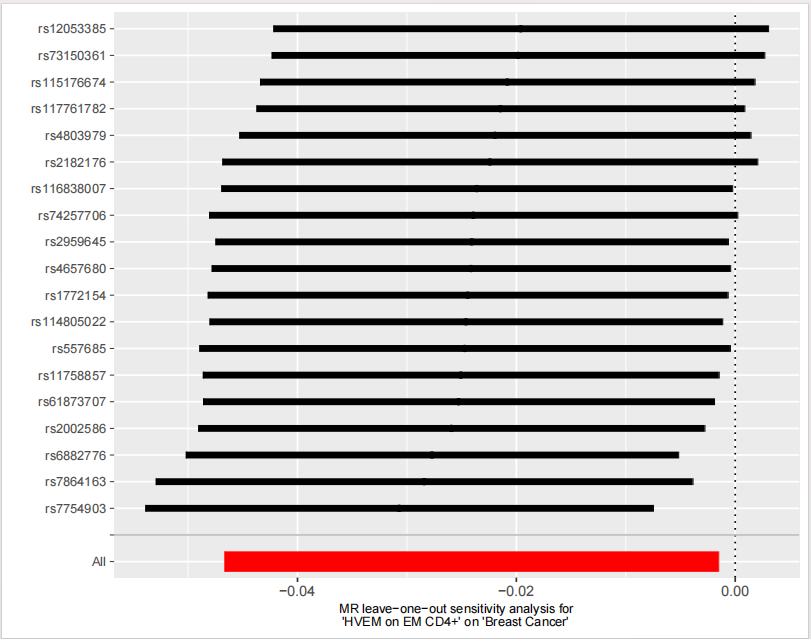

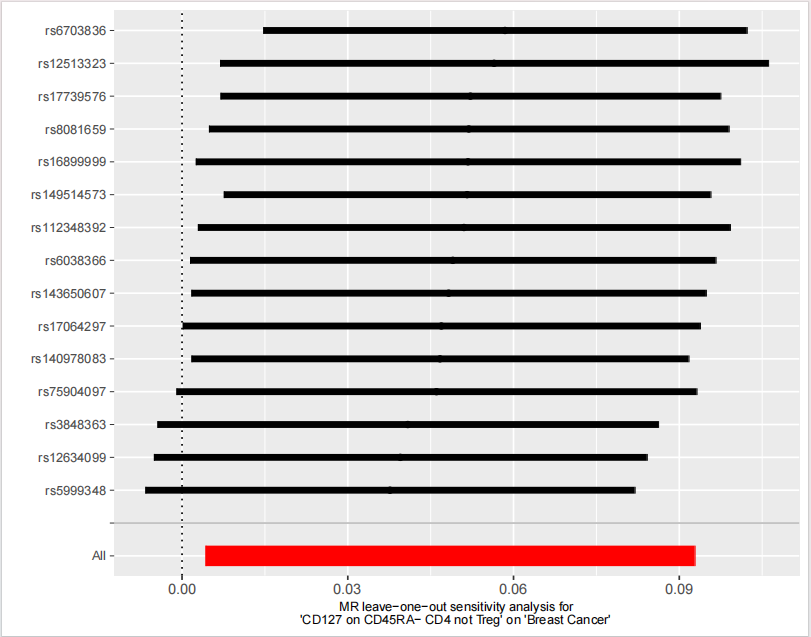

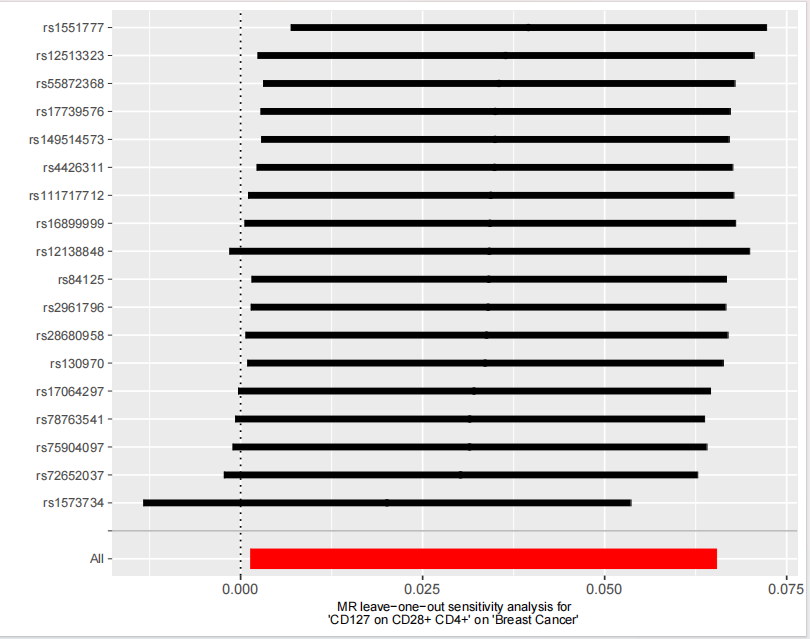

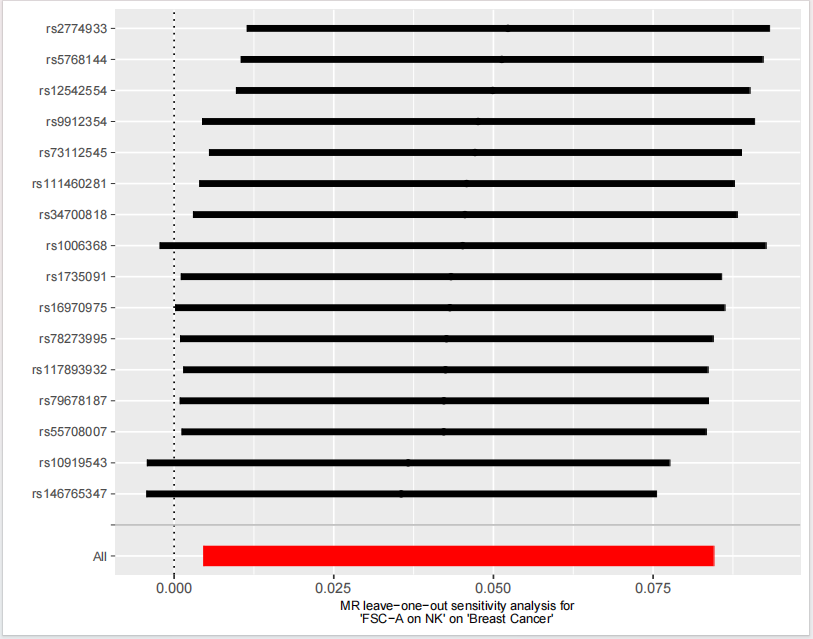

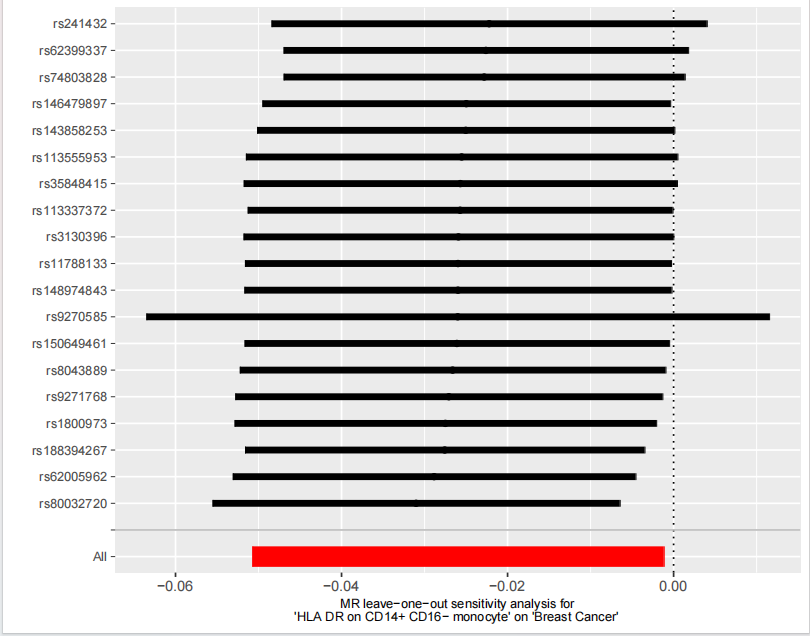

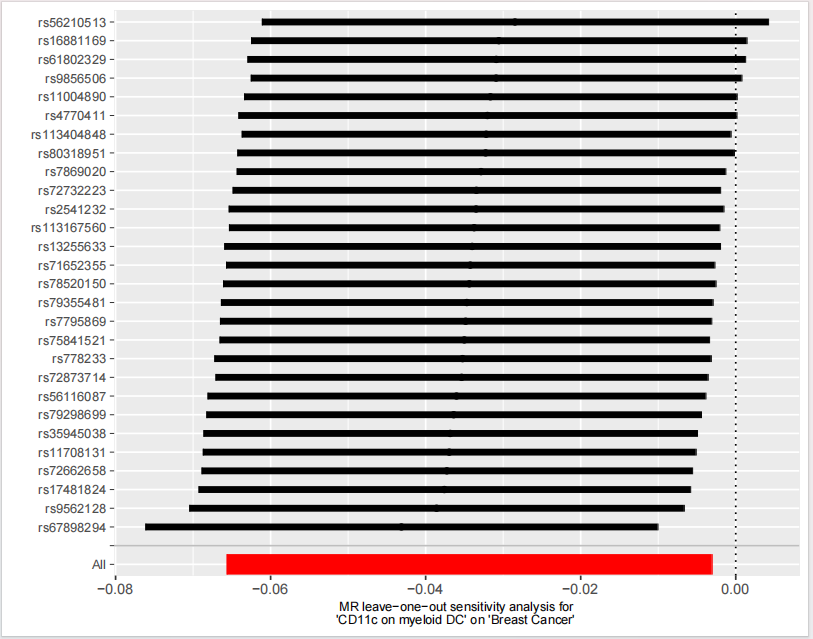

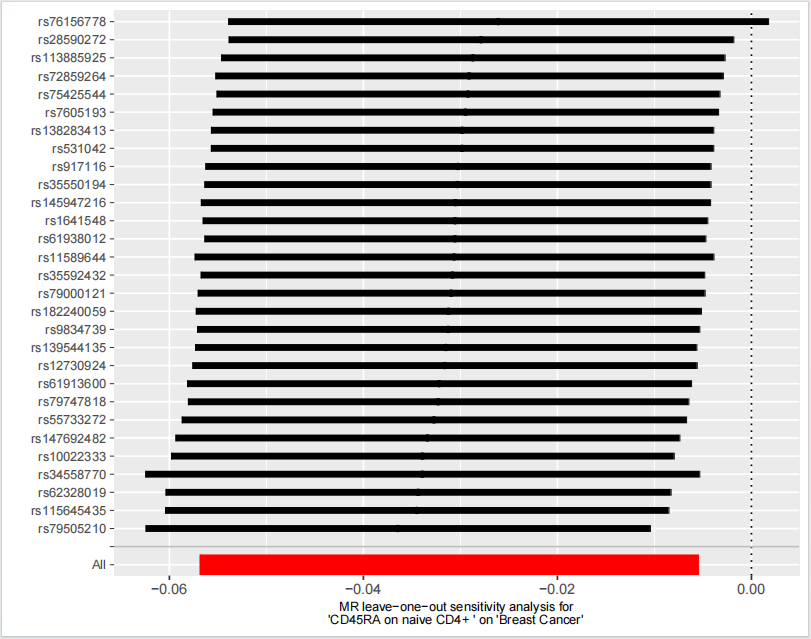

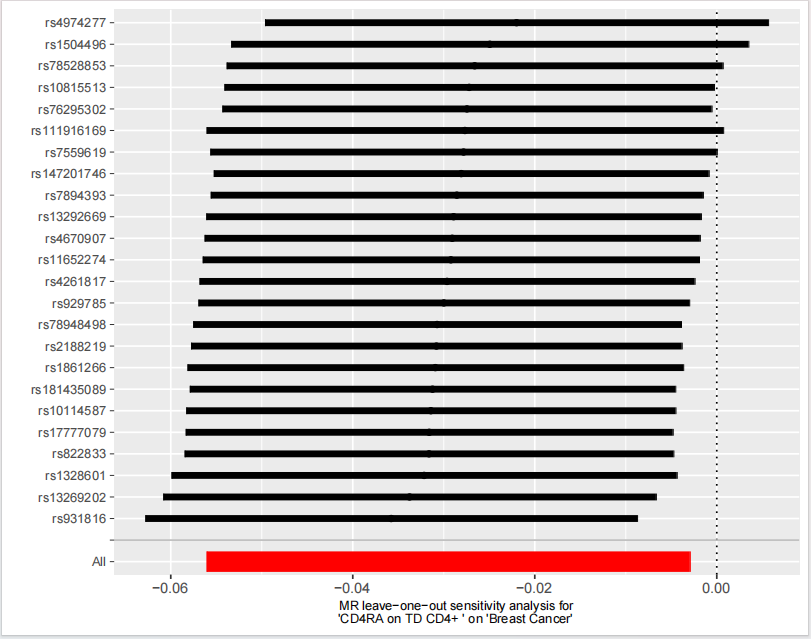

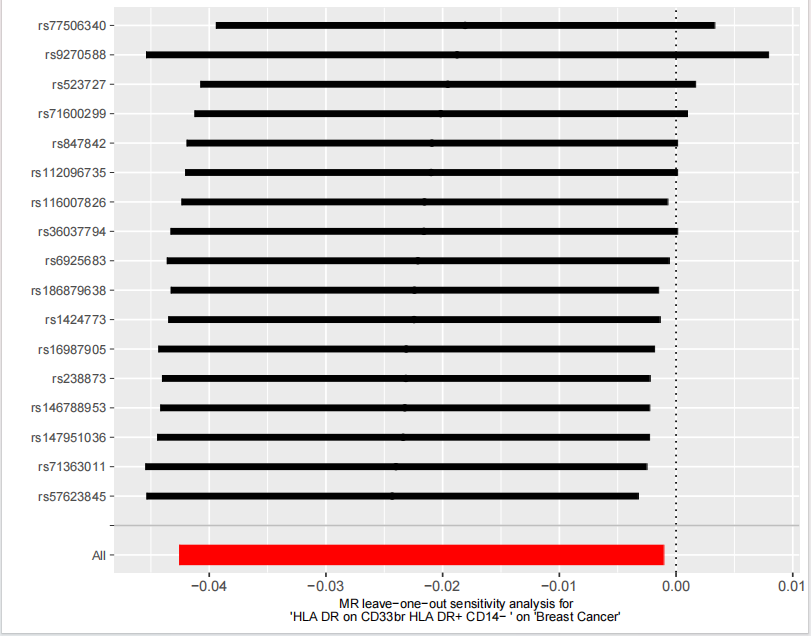

Supplement: Supplementary file 1 [file medi-103-e40815-s001.docx]
